# Supplementary material for: Impact of the SIGN head injury guidelines and NHS 4-hour emergency target on hospital admissions for head injury in Scotland: an interrupted times series
Source: BMJ Open. 2018 Dec 22;8(12):e022279. doi: 10.1136/bmjopen-2018-022279 (PMC6318526; doi:10.1136/bmjopen-2018-022279)
Supplement: Supplementary file 1 [file bmjopen-2018-022279supp001.pdf]

Supplementary Material 1: Summary of Health policies assessed

**Policy**	**Time of Introduction**	**Key Features**
1st SIGN Head Injury Guideline	August 2000	Indication for CT imaging (referenced directly from 2000 SIGN guideline):<br>(1) GCS<13<br>(2) A deteriorating level of consciousness or progressive focal neurological signs<br>(3) Confusion or drowsiness (CGS 13 or 14/15) followed by failure to improve within at most four hours of clinical observation<br>(4) Radiological/clinical evidence of a fracture, whatever the level of consciousness<br>(5) New focal neurological signs which are not getting worse<br>(6) Full consciousness (GCS 15/15) with no fracture but other features, including:<br>– severe and persistent headache<br>– nausea and vomiting<br>– irritability or altered behaviour<br>– a seizure.<br><br>Skull films should be carried out if any of the following apply and if CT is not being performed:<br>If the patient is alert and orientated and obeying commands (GCS 15/15) but:<br>– the mechanism of injury has not been trivial; or<br>– consciousness has been lost; or<br>– the patient has loss of memory or has vomited; or<br>– the scalp has a full thickness laceration or a boggy haematoma; or<br>– the history is inadequate.
4-Hour ED performance target	2004	98% of patients attending the ED to be assessed, treated and either discharged or admitted to hospital within 4 hours of arrival. Financial incentives associated with meeting the target.
2nd Sign Head Injury Guideline	May 2009	Referenced directly from 2nd SIGN Guidelines<br><br>Indications Immediate CT scanning (adult):<br>- GCS<13/15<br>- GCS 13/15 or 14/15 followed by failure to improve within one hour of clinical observation<br>- base of skull or depressed skull fracture and/or suspected penetrating injuries<br>- a deteriorating level of consciousness or new focal neurological signs<br>- GCS 15/15 with severe and persistent headache or two episodes of vomiting<br>- coagulopathy (eg warfarin use) and loss of consciousness or amnesia<br>Indications CT scanning within eight (adult):<br>- age>65 (with loss of consciousness or amnesia)

		- clinical evidence of a skull fracture but no clinical features indicative of an immediate CT scan<br>- any seizure activity<br>- significant retrograde amnesia (>30 minutes)<br>- dangerous mechanism of injury<br><br>Indications Immediate CT scanning (<16 years):<br>- GCS≤13 on assessment in ED<br>- witnessed loss of consciousness >5 minutes<br>- suspicion of open or depressed skull injury or tense fontanelle<br>- focal neurological deficit<br>- any sign of basal skull fracture.<br>Indications CT scanning within 8 hours (<16 years):<br>- presence of any bruise/swelling/laceration >5 cm on the head<br>- post-traumatic seizure<br>- amnesia (anterograde or retrograde) lasting >5 minutes<br>- clinical suspicion of non-accidental head injury<br>- a significant fall<br>- age under one year: GCS<15 in emergency department<br>- three or more discrete episodes of vomiting<br>- abnormal drowsiness.

Supplementary Material 2: Changes to Scotland's population structure

i) Mid-year Estimate Scottish Population 0-15

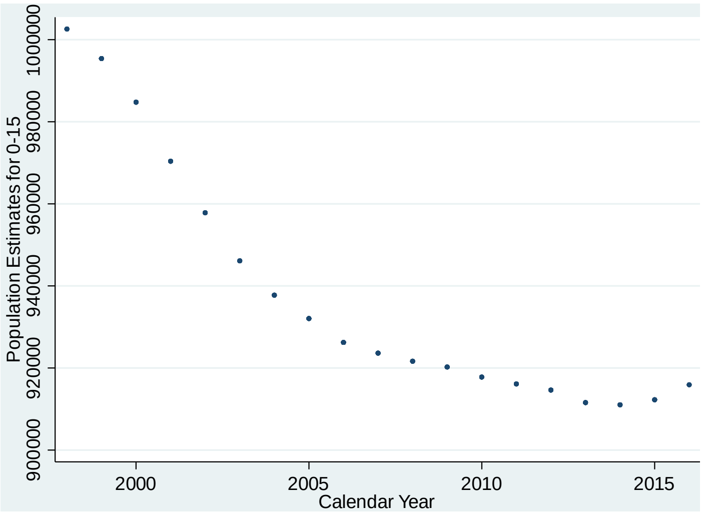

ii) Mid-year Estimate Scottish population 16-64

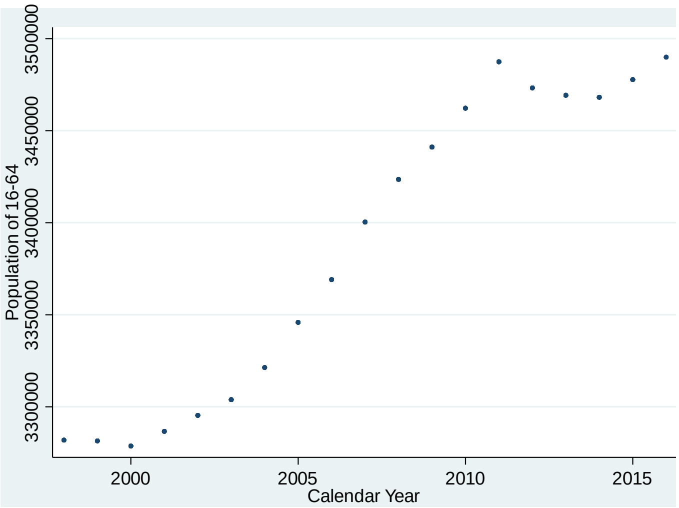

iii) Mid-year Estimate Scottish population 65+

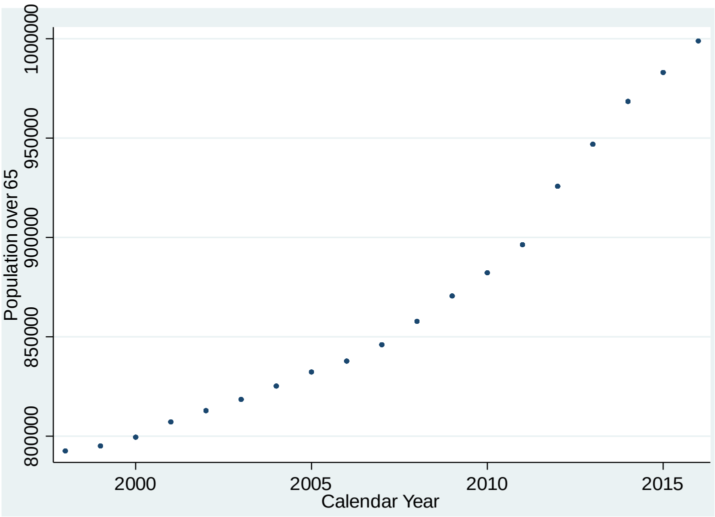

Supplementary Material 3: Increase in admissions related to the introduction of the 4-hour target in the 16-64 age group

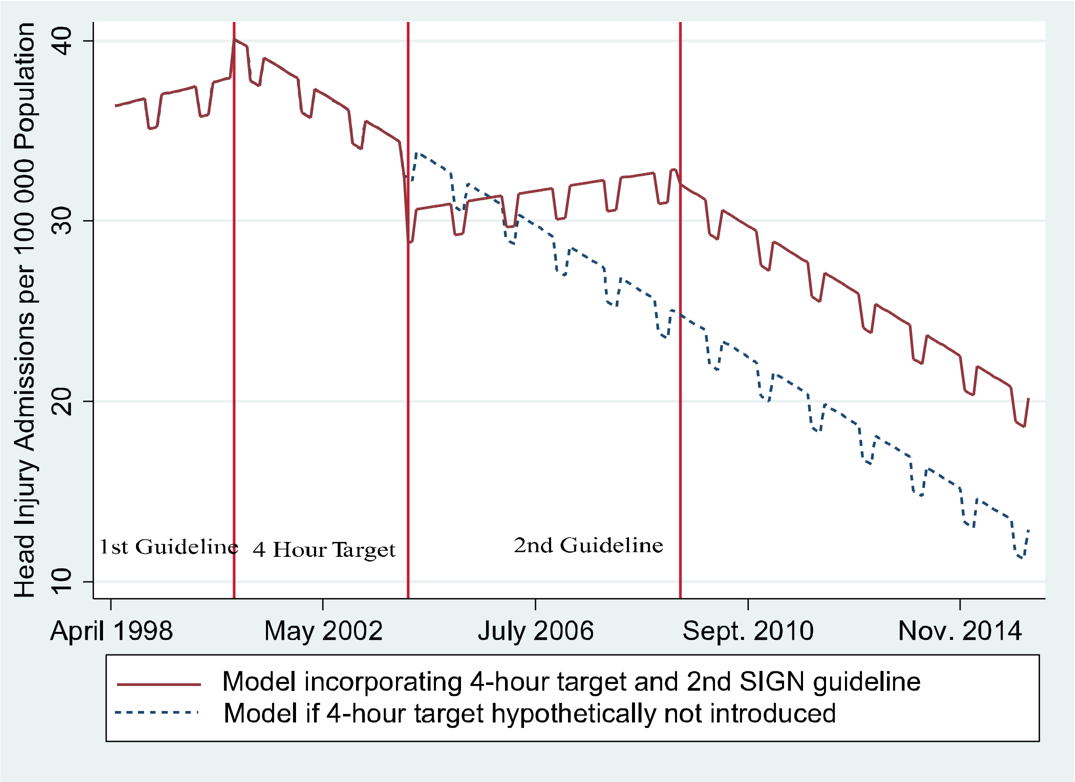

Supplementary Material 4: Estimate of Impact of the SIGN guidelines and introduction of 4-Hour Target admissions for head injury with 12-month time lag

**Age Band**	**Winter**	**Initial Trend**	**1st SIGN Guideline**	**4-hour Target Introduced**	**2nd SIGN Guideline**	**Durbin-Watson Statistic**
**All ages**	-3.00 (95% CI: -3.75 to -2.26) P<0.01	0.04 (95% CI: -0.07 to 0.14) P=0.48	**Change level:** 2.66 (95% CI:0.27 to 5.04) P=0.03	**Change level:** -2.73 (95% CI:-4.85 to -0.61) P<0.01	**Change level:** -2.07 (95% CI:-3.74 to -0.40) P=0.02	Untransformed 1.77 Prais-Winsten 2.00
			**Change trend:** -0.23 (95% CI:-0.38 to -0.08) P<0.01	**Change trend:** 0.25 (95% CI: 0.14 to 0.36) P<0.01	**Change trend:** -0.13 (95% CI: -0.18 to -0.08) P<0.01	
**0-15**	-9.31 (95% CI: -11.05 to -7.59) P<0.01	-0.18 (95% CI:-4.89 to 0.13) P=0.26	**Change level:** 8.53 (95% CI:1.93 to 15.13) P=0.01	**Change level:** 1.61 (95% CI:-4.39 to 7.61) P=0.60	**Change level:** -1.71 (95% CI:-6.61 to 3.20) P=0.49	Untransformed 1.37 Prais-Winsten 1.85
			**Change trend:** -0.17 (95% CI:-0.61 to 0.28) P=0.47	**Change trend:** 0.15 (95% CI: -0.17 to 0.48) P=0.36	**Change trend:** 0.10 (95% CI:-0.50 to 0.26) P=0.18	
**16-64**	-1.80 (95% CI:-2.56 to -1.04) P<0.01	0.06 (95% CI:-0.06 to 0.17) P=0.34	**Change level:** 1.29 (95% CI:-1.28 to 3.85) P=0.32	**Change level:** -4.19 (95% CI:-6.48 to -1.89) P<0.01	**Change level:** -3.06 (95% CI:-4.88 to -1.24) P<0.01	Untransformed 1.57 Prais-Winsten 2.05
			**Change trend:** -0.25 (95% CI:-0.41 to -0.09) P<0.01	**Change trend:** 0.29 (95% CI: -0.17 to 0.40) P<0.01	**Change trend:** -0.25 (95% CI:-0.30 to -0.19) P<0.01	
**65+**	1.69 (95% CI: 0.22 to 3.17) P=0.03	0.17 (95% CI:-0.04 to 0.38) P= 0.10	**Change level:** 3.64 (95% CI:-1.08 to 8.36) P= 0.13	**Change level:** -1.64 (95% CI:-5.83 to 2.55) P=0.44	**Change level:** 2.30 (95% CI:-1.00 to 5.60) P=0.17	Untransformed 1.78 Prais-Winsten 2.00
			**Change trend:** -0.22 (95% CI:-0.51 to 0.07) P=0.14	**Change trend** 0.26 (95% CI: 0.04 to 0.48) P=0.02	**Change trend:** 0.003 (95% CI:-0.10 to 0.10) P=0.95	

Supplementary Material 5: Increase in admissions related to the introduction of the 2nd SIGN guideline

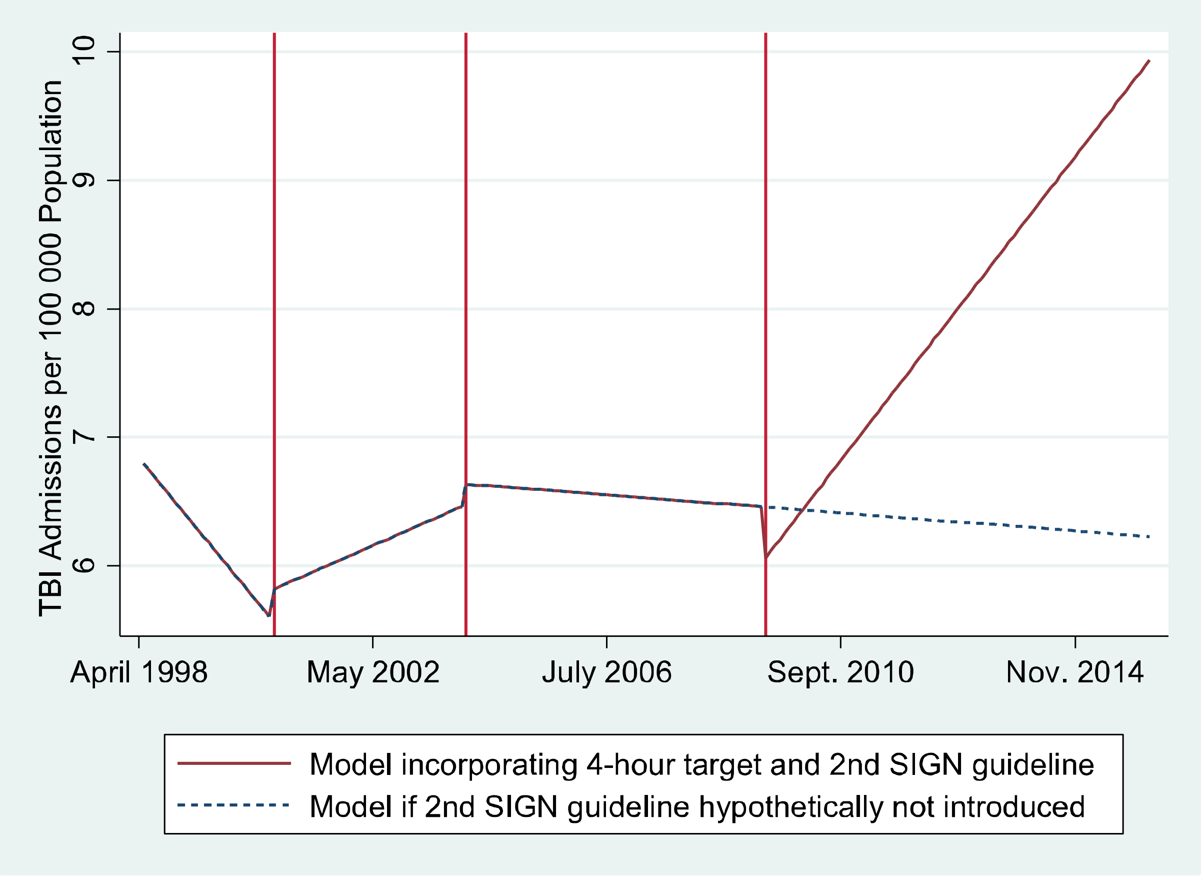

Supplementary Material 6: The impact of the SIGN guidelines on the percentage of admissions for TBI that resulted in neurosurgery

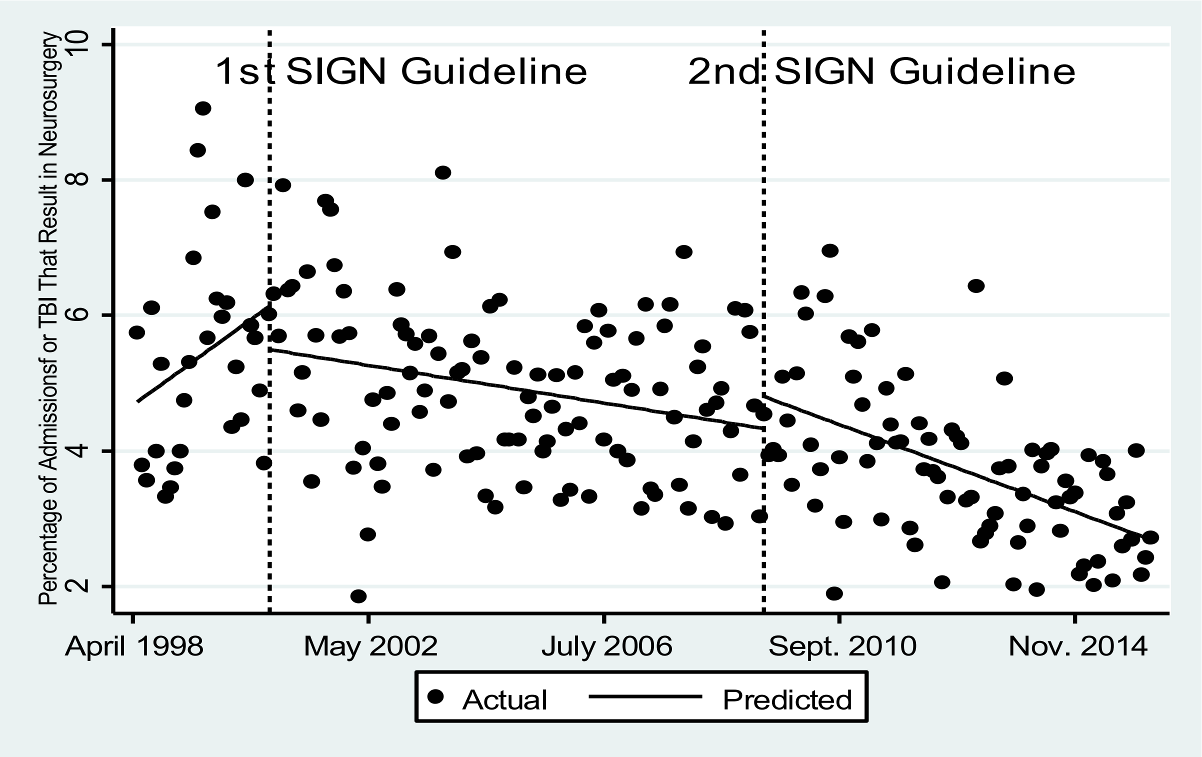

Supplementary Material 7: Estimate of impact of the SIGN guidelines and 4-Hour Target on admissions for Traumatic Brain Injury with 12-month time lag

Outcome	Initial Trend	1st SIGN Guideline	4-hour Target Introduced	2nd SIGN Guideline	Durbin-Watson Statistic
**Admissions for TBI/ 100 000**	-0.05 (95% CI: -0.10 to 0.003) P=0.07	**Change level:** 0.75 (95% CI:-0.34 to 1.84) P=0.18	**Change level:** 0.35 (95% CI:-0.63 to 1.33) P=0.48	**Change level:** -0.16 (95% CI: -0.92 to 0.60) P=0.69	Untransformed 1.44<br>Prais-Winsten 2.03
		**Change trend:** 0.05 (95% CI:-0.02 to 0.12) P=0.16	**Change trend:** -0.01 (95% CI:-0.06 to 0.04) P=0.66	**Change trend:** 0.05 (95% CI:3 to 0.08) P<0.01	
**Percentage TBI admissions neurosurgical**	0.05 (95% CI: -0.01 to 0.11) P=0.10	**Change level:** -0.96 (95% CI:-2.19 to 0.28) P=0.13		**Change level:** 0.17 (95% CI:-0.59 to 0.93) P=0.66	Untransformed 1.85
		**Change trend:** -0.06 (95% CI:-0.12 to 0.003) P=0.06		**Change trend:** -0.02 (95% CI:-0.04 to-0.004) P=0.01	

Supplementary Material 8: The impact of the SIGN guidelines on deaths per 100 000 population within 30 days of admission with traumatic brain injury

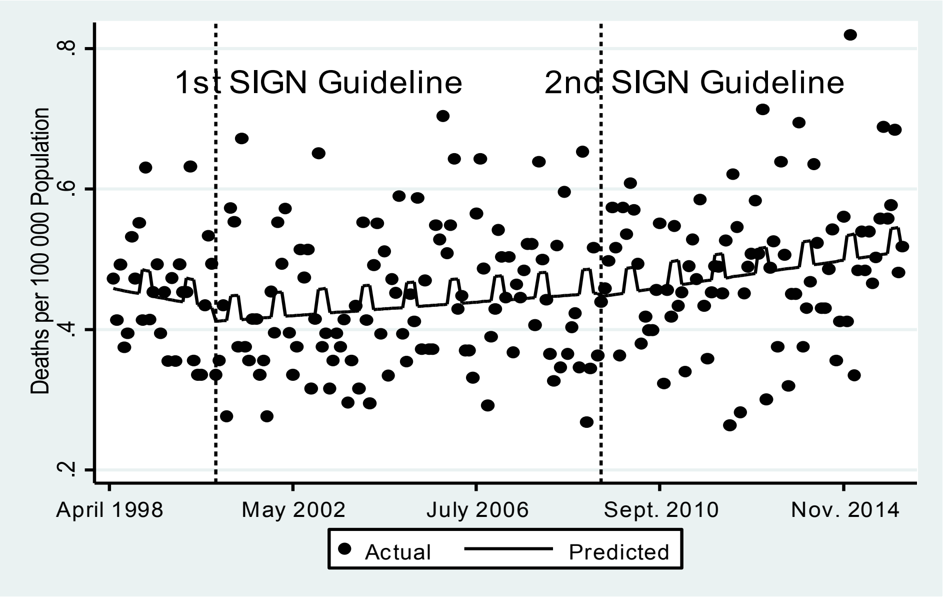

Supplementary Material 9: The impact of the SIGN guidelines on the percentage of patients admitted with TBI who died within 30 days of admission

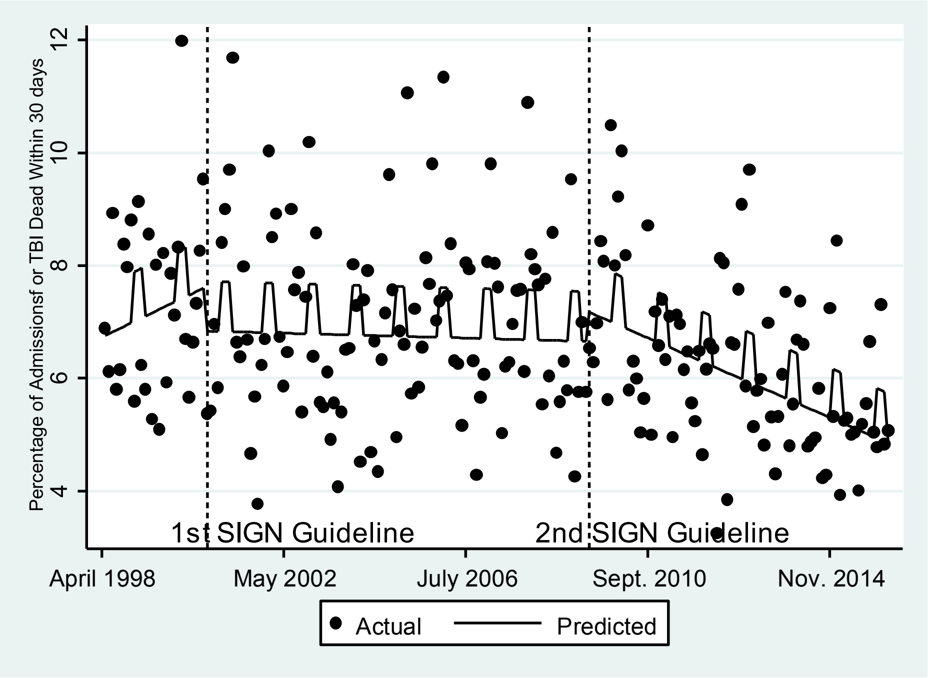

Supplementary Material 10: Estimate of impact of the SIGN guidelines on deaths following admission for Traumatic Brain Injury with 12 month time lag

**Outcome**	**Winter**	**Initial Trend**	**1st SIGN Guideline**	**2nd SIGN Guideline**	**Durbin-Watson Statistic**
Deaths/100 000	0.03 (95% CI:-0.01 to 0.06) P=0.15	-0.001 (95% CI:-0.004 to 0.003) P=0.59	**Change level:** -0.02 (95% CI:- 0.09 to 0.06) P=0.66	**Change level:** -0.03 (95% CI:-0.08 to 0.03) P=0.32	Untransformed 2.29
			**Change trend:** 0.001 (95% CI:-0.002 to 0.005) P=0.44	**Change trend:** 0.001 (95% CI:-0.0002 to 0.002) P=0.10	
Percentage TBI admissions death	0.74 (95% CI: 0.18 to 1.30) P=0.10	0.03 (95% CI:-0.03 to 0.10) P=0.34	**Change level:** -1.06 (95% CI: -2.40 to 0.29) P=0.12	**Change level:** -0.37 (95% CI:-1.20 to 0.47) P=0.39	Untransformed 2.17
			**Change trend:** -0.03 (95% CI:-0.10 to 0.04) P=0.38	**Change trend:** -0.02 (95% CI:-0.04 to -0.004) P=0.02	
