# Supplementary material for: Impact of the SIGN head injury guidelines and NHS 4-hour emergency target on hospital admissions for head injury in Scotland: an interrupted times series
Source: BMJ Open. 2018 Dec 22;8(12):e022279. doi: 10.1136/bmjopen-2018-022279 (PMC6318526; doi:10.1136/bmjopen-2018-022279)
Supplement: Author's manuscript [file bmjopen-2018-022279.draft_revisions.pdf]

BMJ Open is committed to open peer review. As part of this commitment we make the peer review history of every article we publish publicly available.

When an article is published we post the peer reviewers' comments and the authors' responses online. We also post the versions of the paper that were used during peer review. These are the versions that the peer review comments apply to.

The versions of the paper that follow are the versions that were submitted during the peer review process. They are not the versions of record or the final published versions. They should not be cited or distributed as the published version of this manuscript.

BMJ Open is an open access journal and the full, final, typeset and author-corrected version of record of the manuscript is available on our site with no access controls, subscription charges or pay-per-view fees (http://bmjopen.bmj.com).

If you have any questions on BMJ Open's open peer review process please email info.bmjopen@bmj.com

BMJ Open

The impact of the SIGN head injury guidelines and NHS 4-hour Emergency Target on hospital admissions for head injury in Scotland: An Interrupted Times Series.

Journal:	*BMJ Open*
Manuscript ID	bmjopen-2018-022279
Article Type:	Research
Date Submitted by the Author:	14-Feb-2018
Complete List of Authors:	Marincowitz, Carl; University of Hull, Hull York Medical School<br>Lecky, Fiona; University of Sheffield, School of Health and Related Research<br>Morris, Eleanor ; Hull York Medical School<br>Allgar, Victoria; Hull York Medical School<br>Sheldon, Trevor; University of York, Health Sciences
Keywords:	Traumatic Brain Injury, Head Injury, SIGN Guidelines, NHS 4-hour Emergency Department Performance Target, Interrupted Time Series

SCHOLARONE™
Manuscripts

The impact of the SIGN head injury guidelines and NHS 4-hour Emergency Target on hospital admissions for head injury in Scotland: An Interrupted Times Series.

Carl Marincowitz[1] MB BChir, MSc, BA

Fiona E. Lecky[2] MB Ch B, FRCS, DA, MSc, PhD, FCEM

Eleanor Morris[3]

Victoria Allgar[4] PhD, Chartered Statistician, Royal Statistical Society, BSc

Trevor A. Sheldon[5] MSc, MSc, DSc, FMedSci

1. **Corresponding Author.** Hull York Medical School, Allam Medical Building, University of Hull, Hull HU6, UK 7RX, Fax: +44 (0) 1482 464705 Tel +44 (0) 870 1245500
Email: carl.marincowitz@hyms.ac.uk

2. School of Health and Related Research, University of Sheffield, Regent Court, 30 Regent Street, Sheffield, S1 4DA, UK, Fax: +44 (0)114 222 0749 Tel: (+44) (0)114 222 4345,
Email: f.e.lecky@sheffield.ac.uk

3. Hull York Medical School, Allam Medical Building, University of Hull, Hull HU6, UK 7RX, Fax: +44 (0) 1482 464705 Tel +44 (0) 870 1245500 Email: hyem7@hyms.ac.uk

4 Hull York Medical School, John Hughlings Jackson Building, University of York, Heslington, York YO10 5DD Tel: 0870 124 5500, Email: Victoria.Allgar@hyms.ac.uk

5. Department of Health Sciences, University of York, Alcuin Research Resource Centre, Heslington, York, YO10 5DD, Tel +44 (0) 1904 321344, Fax: +44 (0) 1904 32 3433,
E-mail: trevor.sheldon@york.ac.uk

Word Count: 3877

For peer review only - http://bmjopen.bmj.com/site/about/guidelines.xhtml

Abstract

Objectives:

Head injury is a common reason for Emergency Department (ED) attendance. A small number of patients have life-threatening injuries, whilst the majority are discharged. National guidelines (SIGN) were introduced in Scotland with the aim of achieving early identification of those with acute intracranial lesions yet safely reducing hospital admissions.

This study aims to assess the impact of these guidelines and any effect the national 4-hour ED performance targets had on hospital admissions for head injury.

Setting:

All Scottish hospitals between April 1998 and March 2016.

Participants:

Patients admitted to hospital for head injury or traumatic brain injury (TBI) diagnosed by CT imaging identified using administrative Scottish Information Services Division data.

Interventions:

The SIGN head injury guidelines introduced in 2000 and 2009. The 4-hour ED target introduced in 2004.

Outcomes:

The monthly rate of hospital admissions for head injury and traumatic brain injury.

Study Design:

An interrupted time series analysis.

Results:

The 1st guideline was associated with a reduction in monthly admissions of 0.14 (95% CI:0.09 to 4.83) per 100, 000 population. The 4-hour target was associated with a monthly increase in admissions of 0.13 (95% CI:0.06 to 0.20) per 100, 000 population. The 2nd guideline reduced monthly admissions by 0.09 (95% CI:-0.13 to -0.05) per 100, 000 population. These effects varied between age groups.

The guidelines were associated with increased admissions for patients with injuries identified by CT imaging- Guideline 1: 0.06 (95% CI: 0.004 to 0.12); Guideline 2: 0.05 (95% CI: 0.04 to 0.06) per 100 000 population.

Conclusion:

Increased CT imaging of head injured patients recommended by SIGN guidelines reduced hospital admissions. The 4-hour ED target and the increased identification of TBI by CT imaging acted to undermine this effect.

For peer review only - http://bmjopen.bmj.com/site/about/guidelines.xhtml

Strengths and limitations of this study:

This is the first study to assess the impact of the SIGN head injury guidelines and 4-hour Emergency Department target on hospital admissions for head injury.

We used the robust method of interrupted time series analysis and found the SIGN guidelines acted to reduce hospital admissions, but the 4-hour target increased hospital admissions.

Due to the aggregated nature of the available data we were unable to perform some age group specific and injury sub-group sensitivity analysis.

Keywords:

Traumatic Brain Injury, Head Injury, SIGN Guidelines, NHS 4-hour Emergency Department Performance Target and Interrupted Time Series.

For peer review only

Background:

There are 1.4 million annual attendances to Emergency Departments (ED) in England and Wales following a head injury (blunt trauma to the head).[1] In Scotland an estimated 6.6% of ED attendances are for head injury.[2] Approximately 95% of patients present with an initial Glasgow Coma Scale (GCS) of 13-15, indicating normal or mildly impaired conscious level and are defined as having a "minor" head injury .[1 3]

Around 1% of minor head injured patients have life-threatening traumatic brain injuries (TBI) (injury to the brain/ functional impairment due to external force) but this may not be initially clinically apparent.[1] Early identification of severe TBI can facilitate life-saving neurosurgery.[4] The clinical challenge is to differentiate patients with life-threatening TBI who present with a high conscious level from patients who can be discharged safely. This can be achieved through observation for deterioration or cranial CT imaging.[5] The health services challenge is finding a way of differentiating these groups in a way that minimises unnecessary imaging or inpatient hospital admissions of patients without clinically important TBI. Research has focused on developing clinical decision rules that, using clinical assessment, triage patients at risk of life-threatening TBI to CT imaging and allow the discharge of low-risk patients. The most validated is the Canadian CT Head Rule (CCHR) and this forms the basis of the Scottish Intercollegiate Guidelines Network (SIGN) and English National Institute for Health and Care Excellence (NICE) head injury guidelines.[1 2 6]

Two iterations of the SIGN guidelines have been introduced (Supplementary Material 1).[2 7] The first recommended increased CT imaging of head injured patients but still featured a role for skull x-rays and admission for observation.[7] The 2nd SIGN guideline extended CT imaging further.[2] A study assessing trends in hospital admissions for TBI in Scotland between 1998 and 2009 found changes in rates of admissions at specific time points after 2000, but the analysis was data driven and does not explicitly assess the impact of the SIGN guidelines.[8] Implementation studies of NICE head injury guidelines in England suggested that the cost of increased CT imaging would be offset by a reduction in inpatient hospital admissions as patients admitted for observation would be dicharged from the ED following normal CT imaging.[9-11]

For peer review only - http://bmjopen.bmj.com/site/about/guidelines.xhtml

However, a study using English Hospital Episodes Statistics (HES) found head injury inpatient admissions increased following the introduction of the NICE guidelines, possibly due to the near simultaneous introduction of the 4-hour ED target. Supplementary material 1 presents the details of the 4-target and in 2002 around 23% of all patients in the UK remained in the ED for longer than 4-hours.[12] The target could act to incentivise inpatient admissions for patients at medium risk for TBI, who require imaging within 8 hours, to avoid "breaches". Previously they would have been imaged in the ED and the majority discharged. A further cause of increased admissions could be the detection of brain injuries previously not identified, some without clinically significant sequelae, due to increased imaging.[13] In Scotland the 4-hour target was not introduced at the same time as the SIGN guidelines. This provides a unique opportunity to assess the independent effects of these policy interventions.

Aims:

This study aims to assess the impact of the introduction of the SIGN head injury guidelines and the 4-hour target on the rate of hospital admissions in patients with head injury and explores the extent to which any increase in admissions were due to the identification of more TBI.

Methods:

Study Design:

Interrupted time series analysis is an established and robust method for the evaluation of health policies implemented at discrete time points.[14 15]

Dataset:

The Scottish Information Services Division (ISD) contains a repository of information routinely collected at discharge from hospital for all non-obstetric and non-psychiatric inpatient hospital admissions in Scotland. Since 1996 reason for admission has been categorised using ICD10 diagnostic coding and since 1989 interventions have been coded using the OPSC4 classification.[16-18] This repository was used, for the period 1998-2016, to generate monthly numbers of: patients admitted with ICD10 coding for head injury, patients admitted with ICD coding indicating TBI identified by CT imaging, neurosurgical

For peer review only - http://bmjopen.bmj.com/site/about/guidelines.xhtml

For peer review only

interventions and deaths within 30 days of admission for patients with CT evidence of TBI. Diagnostic codes were counted if they were either primary or secondary diagnoses.

We selected an ICD10 code subset definition of head injury that includes all possible definitions of TBI and head injury (Table 1). The ICD10 codes used are consistent with those selected to explore the NICE guideline's effect on hospital admissions for head injury, with the addition of codes for crush injuries.[13] We selected an ICD10 code subset to define TBI that corresponds to injuries identified by CT imaging including codes for traumatic intra-cranial haemorrhages and skull fractures, but excluded codes for concussion and other clinical diagnoses (Table 1).

Outcomes:

Admissions for head injury:

The monthly number of patients admitted to hospital with one or more ICD10 code indicating head injury between April 1998 and March 2016 was generated by the ISD. As the SIGN guidelines have specific recommendations regarding paediatric patients and patients 65 and over, admissions were stratified into: 0-15, 16-64 and ≥65 age groupings. Yearly and monthly number of admissions were converted into a rate per 100, 000 population using Nomis Office of National Statistics (ONS) mid-year population estimates for Scotland for each age grouping (Supplementary Material 2).

Admissions for Traumatic Brain Injury:

The ISD generated the monthly number of patients admitted to hospital with at least one ICD10 code sub-classification indicting an admission for TBI. Monthly and yearly rates per 100, 000 population of admissions for TBI were calculated using Nomis ONS mid-year Scottish population estimates.

The ISD also provided the monthly number of patients admitted with an ICD10 code indicating TBI that had one or more OPSC4 neurosurgical codes (Table 2). A monthly proportion of admissions for TBI that resulted in neurosurgery was estimated. The small number of monthly TBI admissions prevented release of data stratified by age group

For peer review only - http://bmjopen.bmj.com/site/about/guidelines.xhtml

Table 1: Annual trends admissions for head injury and TBI

Year	Annual rates head injury admissions per 100, 000 population each age group.*				Annual rates TBI admissions per 100, 000 population all age.** (Percentage annual rate all head injury admissions)
	All ages	0-15	16-64	65+	
1999	484	558	453	536	77 (15.9%)
2000	476	519	449	556	70 (14.7%)
2001	493	561	457	578	72 (14.6%)
2002	489	556	447	598	71 (14.5%)
2003	451	521	404	581	76 (16.9%)
2004	435	495	381	589	82 (18.9%)
2005	417	497	350	591	78 (18.7%)
2006	427	477	369	585	79 (18.5%)
2007	442	441	393	653	79 (17.9%)
2008	435	400	386	670	76 (17.4%)
2009	440	406	377	709	79 (18%)
2010	400	361	337	709	80 (20%)
2011	421	369	352	741	91 (21.6%)
2012	411	339	321	817	89 (21.7%)
2013	396	307	293	845	97 (24.5%)
2014	385	319	268	848	102 (26.5%)
2015	373	316	251	867	123 (33%)

*ICD10 codes for head injury: S00-S09, T04.0 and T06.0

** ICD10 codes for TBI: S02.0, S02.1, S02.7, S02.8, S02.9, S06.1, S06.3, S06.4, S06.5, S06.6, S06.7, S06.8, S06.9, T04.0 and T06.0

For peer review only - http://bmjopen.bmj.com/site/about/guidelines.xhtml

Table 2: Impact of the SIGN guidelines on number of admissions and deaths from Traumatic Brain Injury per 100, 000 Scottish population

**Outcome**	**Winter Effect**	**Initial Trend**	**1st SIGN Guideline**	**4-hour Target Introduced**	**2nd SIGN Guideline**	**Durbin-Watson Statistic**
**Admissions for TBI/100 000**		-0.04 (95% CI: -0.09 to 0.004) P=0.07	**Change level:** 0.26 (95% CI:- 0.74 to 1.26) P=0.61	**Change level:** 0.16 (95% CI: -0.67 to 0.99) P=0.71	**Change level:** -0.39 (95% CI: -1.09 to 0.30) P=0.27	Untransformed 1.46 Prais-Winsten 2.02
			**Change trend:** 0.06 (95% CI: 0.004 to 0.12) P=0.04	**Change trend:** -0.02 (95% CI: -0.05 to 0.01) P=0.24	**Change trend:** 0.05 (95% CI: 0.03 to 0.07) P<0.01	
**Percentage TBI admissions neurosurgical+**		0.05 (95% CI: -0.01 to 0.11) P=0.10	**Change level:** -0.64 (95% CI: -1.83 to 0.56) P=0.29		**Change level:** 0.47 (95% CI:-0.17 to 1.12) P=0.15	Untransformed 1.81
			**Change trend:** -0.06 (95% CI: -0.12 to 0.001) P=0.047		**Change trend:** -0.01 (95% CI:-0.03 to -0.003) P=0.01	
**Deaths/100 000**	0.03 (95% CI: 0.001 to 0.07) P=0.04	-0.001 (95% CI:-0.004 to 0.002) P=0.57	**Change level:** -0.02 (95% CI:- 0.09 to 0.06) P=0.62		**Change level:** -0.01 (95% CI:-0.06 to 0.05) P=0.85	Untransformed 2.3
			**Change trend:** 0.001 (95% CI:-0.002 to 0.005) P=0.44		**Change trend:** 0.0004 (95% CI: -0.001 to 0.001) P=0.46	
**Percentage TBI admissions death**	0.88 (95% CI: 0.36 to 1.41) P<0.01	0.03 (95% CI: -0.03 to 0.10) P=0.35	**Change level:** -0.79 (95% CI: -2.12 to 0.54) P=0.24		**Change level:** 0.52 (95% CI: -0.32 to 1.35) P=0.22	Untransformed 2.08
			**Change trend:** -0.03 (95% CI: -0.10 to 0.03) P=0.33		**Change trend:** -0.03 (95% CI: -0.04 to - 0.01) P<0.01	

+ Neurosurgical procedure defined as 1 or more OPSC4 codes: A05.2, A05.3, A05.4, A05.8, A05.9, A40.1, A40.8, A40.9, A41.1, A41.8, A41.9, V03.1, V03.2, V03.4, V03.6, V03.7, V03.8, V03.9, V05.3 and V05.4

For peer review only

For peer review only - http://bmjopen.bmj.com/site/about/guidelines.xhtml

Deaths related to Traumatic Brain Injury:

The ISD provided the monthly number of patients who died within 30 days of admission with at least one ICD10 code for TBI. This was converted into a rate using Nomis ONS mid-year Scottish population estimates. The monthly proportion of patients admitted with TBI who died within 30 days of admission was estimated using the total number of monthly admissions for ICD10 codes that corresponded to TBI.

Statistical analysis:

A monthly time series of the rate of inpatient hospital admissions for head injury ICD codes was plotted from April 1998 to March 2016. An interrupted times series analysis was completed assessing the impact of SIGN head injury guidelines and the 4-hour ED target using the ITSA package in STATA 14.[19 20] The model included 3 intervention time points: the introduction of the 1st SIGN guideline in August 2000, the introduction of the 4-hour target in 2004 and the introduction of the 2nd SIGN guideline in May 2009. Analysis was stratified into 3 age groups: 0-15, 16-64 and ≥65s. A segmented regression model predicting the rate of hospital admissions per 100, 000 population in each age grouping per month was estimated.[15]

Autocorrelation of the residuals was assessed using the Durbin-Watson and Rho statistic.[15 21] Where there was sufficient deviation from a Durbin Watson statistic of 2 and the Rho statistic was not statistically significant, the Prais-Winsten transformation was used to adjust for auto-correlation.[15] Seasonality was assessed by introducing a dummy variable to the model in which winter months (December, January and February) were coded 1 and was included in the model when a statistically significant predictor.

The interrupted time series analysis was repeated to assess: the impact of the SIGN guidelines and 4-hour target on the rate of hospital admissions for patients with an ICD10 code indicating TBI, the impact of SIGN guidelines on the proportion of inpatient admissions for TBI that resulted in neurosurgery or death and the death rate within 30 days of admission for TBI. It was thought a priori that the 4-hour target would not plausibly have affected neurosurgery or deaths. This was confirmed through visual inspection and was excluded from analysis of these outcomes.

For peer review only - http://bmjopen.bmj.com/site/about/guidelines.xhtml

Estimates of the impact of the policy interventions on inpatient admissions were made by using the pre-intervention model to estimate hypothetical monthly rates of admissions if no intervention had occurred. These were subtracted from the monthly admission rates estimated by the post-intervention model.[14] To explore any effect of a policy implementation lag a sensitivity analysis was performed for all the models in which the 12 months immediately following the introduction of a policy change, were excluded from analysis.[15]

Results:

Head injury inpatient hospital admissions:

Table 1 show yearly rates of inpatient hospital admissions. Fig. 1 and Table 3 present the results of the interrupted time series assessing the impact of the SIGN guidelines and 4-hour ED target on monthly head injury admissions. Admission rates and estimates of effect are reported per 100, 000 population in each age grouping. The SIGN guidelines and 4-hour target were associated with a significant change in the total level and trend of the rate admissions for head injury (Fig 1a). The effect varied between age group.

0-15 Age Group:

Monthly inpatient admissions fell from 47.18 to 24.02 per 100, 000 per month over the time period (Fig. 1b). Neither SIGN guideline nor the introduction of the 4-hour target significantly affected the underlying reducing trend in hospital admissions but the first guideline was associated with a borderline statistically significant increase in level (6.06; 95% CI: -0.49 to 12.62) (Table 3). Admissions were less likely to occur in winter months (-9.19; 95% CI: -10.81 to -7.57) (Table 3).

16-64 Age Group:

Inpatient admissions fell from 36.39 to 20.20 per 100, 000 per month from April 1998 to March 2016 (Fig 1c). Before the 1st SIGN guideline hospital admissions were increasing monthly (0.04; 95% CI: -0.08 to 0.15) (Table 3 and Fig. 1c). The 1st guideline was associated with a declining monthly trend in admissions (-0.20; 95% CI:-0.35 to -0.05). The 4-hour target was associated with an initial fall in the number of inpatient admissions (-3.54; 95% CI: -5.76 to -1.33) but subsequent trend of increasing monthly admissions (0.18; 95% CI:

For peer review only - http://bmjopen.bmj.com/site/about/guidelines.xhtml

0.10 to 0.27). The 2nd guideline was associated with a return to a declining trend in admissions (-0.18; 95% CI:-0.23 to -0.13). Inpatient admissions were reduced in winter months (-1.74; 95% CI:-2.48 to -1.01).

The trend following the introduction of the 1st SIGN guideline hypothetically continued in the period after the introduction of the 4-hour target is shown in Supplementary Material 3. By subtracting this from the model that incorporated the introduction of the 4-hour target and the 2nd SIGN guideline we estimate that from January 2004 to March 2016 the introduction of the 4-hour target was associated with an additional 745 hospital admissions per 100 000 population aged 16-64.

≥65 Age Group:

Monthly admissions increased from 40.00 to 76.09 over the time period (Fig. 1d). The only statistically significant change in the underlying trend was at the introduction of the 4-hour target which was associated with an acceleration in the increase in hospital admissions (0.15; 95% CI: 0.01 to 0.28) (Table 3 and Fig. 1d). Winter months were associated with increased hospital admissions (1.67; 95% CI: 0.32 to 3.02).

Sensitivity Analysis for 12-month implementation lag:

The introduction of an intervention lag in the model (Supplementary Material 4) did not materially affect estimates of effect associated with interventions.

Inpatient hospital admissions for Traumatic Brain Injury on CT scan:

Admission per 100, 000 per month increased from 6.85 to 10.21 over the time period (Fig. 2). Before the 1st SIGN guideline hospital admissions were decreasing (-0.04; 95% CI:-0.09 to 0.004) (Table 2 and Fig. 2). The introduction of the 1st guideline was associated with a trend of increasing admissions (0.06; 95% CI: 0.004 to 0.12). The 4-hour target was not associated with a significant change in level or trend. The introduction of the 2nd SIGN guideline was associated with an acceleration in the increase in admissions (0.05; 95% CI: 0.04 to 0.06).

Comparing admissions for TBI that would have occurred if the 2nd SIGN guideline had not been introduced with the empirically derived model indicates that from May 2009 to March 2016 the introduction of the 2nd SIGN guideline was associated with an additional 138 hospital admissions per 100, 000 population (Supplementary Material 5).

For peer review only - http://bmjopen.bmj.com/site/about/guidelines.xhtml

Table 3: Impact of the SIGN guidelines and introduction of 4-Hour ED Target on number of Head Injury Admissions per 100, 000 Scottish population by age group

Age Band	Winter Effect	Initial Trend	1st SIGN Guideline	4-hour Target Introduced	2nd SIGN Guideline	Durbin-Watson Statistic
**All ages**	-3.00 (95% CI:-3.78 to -2.30) P<0.01	0.04 (95% CI: -0.08 to 0.15) P=0.53	Change level: 2.45 (95% CI:0.09 to 4.83) P=0.04	Change level: -2.89 (95% CI:-4.84 to -0.95) P<0.01	Change level: -0.84 (95% CI:-2.50 to 0.78) P=0.31	Untransformed 1.68 Prais-Winsten 2.02
			Change trend: -0.14 (95% CI: -0.27 to -0.01) P=0.03	Change trend: 0.13 (95% CI: 0.06 to 0.20) P<0.01	Change trend: -0.09 (95% CI:-0.13 to -0.05) P<0.01	
**0-15**	-9.19 (95% CI: -10.81 to -7.57) P<0.01	-0.21 (95% CI: -0.53 to 0.12) P=0.22	Change level: 6.06 (95% CI:-0.49 to 12.62) P=0.07	Change level: -0.41 (95% CI: -5.98 to 5.17) P=0.89	Change level: -0.54 (95% CI:-5.31 to 4.22) P=0.82	Untransformed 1.34 Prais-Winsten 1.87
			Change trend: -0.10 (95% CI:-0.29 to 0.49) P=0.61	Change trend: -0.05 (95% CI: -0.27 to 0.16) P=0.63	Change trend: 0.06 (95% CI:-0.07 to 0.18) P=0.37	
**16-64**	-1.75 (95% CI:-2.48 to -1.01) P<0.01	0.06 (95% CI: -0.07 to 0.19) P=0.39	Change level: 2.12 (95% CI:-0.54 to 4.79) P=0.12	Change level: -3.54 (95% CI:-5.76 to -1.33) P<0.01	Change level: -0.87 (95% CI: -2.74 to 0.99)P=0.36	Untransformed 1.45 Prais-Winsten 2.10
			Change trend: -0.20 (95% CI: -0.35 to -0.05) P<0.01	Change trend: 0.18 (95% CI: -0.10 to 0.27) P<0.01	Change trend: -0.18 (95% CI:-0.23 to -0.13)P<0.01	
**65+**	1.67 (95% CI: 0.32 to 3.02) P=0.02	0.17(95% CI: -0.04 to 0.39) P=0.11	Change level: 2.28 (95% CI: -2.13 to 6.86) P= 0.30	Change level: -2.70 (95% CI: -6.39 to 0.99) P=0.15	Change level: 0.85 (95% CI:-2.22 to 3.93)P=0.59	Untransformed 1.70 Prais-Winsten 2.00
			Change trend: -0.16 (95% CI: -0.40 to 0.09) P=0.21	Change trend 0.15 (95% CI: 0.01 to 0.28) P=0.03	Change trend: 0.05 (95% CI: -0.03 to 0.12) P=0.23	

For peer review only - http://bmjopen.bmj.com/site/about/guidelines.xhtml

Both SIGN guidelines were associated with a reduction in trend in the percentage of TBI admissions resulting in neurosurgery: 1st guideline (-0.06; 95% CI: -0.12 to 0.001) and 2nd guideline (-0.01; 95% CI: -0.03 to -0.003) (Table 2 and supplementary material 6). A 12-month implementation lag did not materially affect the estimates (Supplementary Material 7).

Deaths following admission for TBI:

Neither guideline was associated with a change in level or trend in monthly death rate within 30 days of admission with an ICD 10 code indicating TBI (Table 2 and Supplementary Material 8). The introduction of the 2nd SIGN guideline was associated with a significant reduction in the underlying trend in the monthly percentage of inpatient admissions for TBI that resulted in death within 30 days (-0.03; 95% CI: -0.04 to -0.01) (Table 2 and Supplementary Material 9).

Introduction of a 12-month implementation lag into the models did not materially change the estimates (Supplementary Material 10).

Discussion:

Summary:

To our knowledge, this is the first study to evaluate the impact of the SIGN guidelines and the 4-hour ED target on head injury hospital admissions. We found evidence that the SIGN guidelines reduced inpatient admissions, the effect varying by age group. In the 16-64 age group both guidelines were associated with a reduction in hospital admissions (Fig. 1c and Table 3). This may be due to increased CT imaging in the ED identifying more patients without TBI safe for discharge. In the paediatric population there was an underlying trend of reducing hospital admissions that the guidelines did not appear to affect. In the ≥65 age group neither SIGN guideline iteration acted to offset the secular trend of increasing hospital admissions. Inpatient admissions increased in winter months in the ≥65 age group but were reduced in other age groups. Falls from standing are known to be the most common cause of brain injuries in those ≥65, whilst assault and road traffic accidents are more common causes of injuries in younger patients.[22] Weather conditions during winter

For peer review only - http://bmjopen.bmj.com/site/about/guidelines.xhtml

For peer review only

may increase the likelihood of falls from standing whilst reducing the prevalence of assault or road traffic accidents.[23]

There is controversy regarding which ICD10 codes correspond to clinical definitions of head injury and TBI with inconsistent sets of ICD10 codes used to encompass both.[24] We selected subsets of codes for head injury that are likely to be sensitive to changes in admission practice related to increased diagnostic precision of TBI from increased CT imaging and inpatient admissions of patients awaiting an exclusion of TBI due to the 4-hour target. Our ICD10 code selection for TBI was intended to encompass radiologically detected injuries as this outcome would be sensitive to increased diagnoses of TBI by CT imaging.

The introduction of both SIGN guideline iterations were associated with an increased in hospital admissions for patients with TBI and a reduction in the proportion of inpatient admissions that resulted in neurosurgery or death (Table 2). The guidelines may have acted, as previously hypothesised, to increase CT diagnosis and admissions of patients with brain injuries of lower severity who do not require intervention.[13]The 4-hour target was associated with an increase in hospital admissions for adults (Fig. 1c, 1d and Table 3). This effect was reversed by the 2nd SIGN guideline in the 16-64 but not the ≥65 age group.

Strengths:

We have used a time series of 216 data points and followed established techniques to control for seasonal factors and auto-correlation.[15] We have adjusted for population factors using mid-year population estimates that incorporate the changing demography of Scotland's population. The models constructed were robust to sensitivity analysis for time lags.

Weaknesses:

Ideally the effects of guideline implementation and policy interventions would be assessed using randomised control trials. However, interrupted time series analysis (one of the most rigorous of the quasi-experimental study designs) is becoming increasingly popular particular for the evaluation of health care practice, programmes and policy because it allows strong causal inferences when interventions are introduced at specific time points. Discontinuities in outcomes, observed at or shortly after the time of intervention, are

For peer review only - http://bmjopen.bmj.com/site/about/guidelines.xhtml

persuasive evidence of an effect with high internal and external validity.[14 15 25 26] The method has limitations notably the potential for observed discontinuities to result from co-interventions instead of the health policies under investigation. We are unaware however of other policies or sudden changes to the population of Scotland that could account for the observed changes in admissions for head injury in Scotland at the time of the either the introduction of the SIGN guidelines or the 4-hour ED target.

This study used routinely collected Scottish Information Services Division (ISD) data, and administrative data should be approached with some caution.[27] There may be inaccuracies in diagnostic codes due to coding errors. However, there were no changes to the cohort of admitted patients that data was collected on during the study period and ISD data has been found to be both sufficiently reliably and comprehensively collected to support its use in research.[28 29] Furthermore, random poor coding, without changes in coding practice, are unlikely to result in sharp discontinuities at the specific time points of the policies considered here. ICD coding changed in 1996, so we only used ISD data from 1998 to give time for adjustment. This limited the number of data points before the 1st SIGN guideline, so we may have lacked the power to detect some changes associated with the 1st guideline as statistically significant (Table 3 and Fig. 1).

We could not stratify all the analysis by age group as the small number of some outcomes prevented release of aggregated data. A sensitivity analysis using ICD10 injury subtypes was also not possible due to the aggregated nature of the available data. A more sensitive outcome measure for changes in admission practice due to either the SIGN guidelines or 4-hour target may be the proportion of attendances to the ED following head injury that result in inpatient hospital admission. TBI has become more common in the elderly and if analysis of the proportion of ED attendances that resulted in inpatient admission was stratified by age this would account for age group differences in incidence of injuries .[30]

There is evidence that deaths from severe TBI fell following the introduction of the NICE guidelines.[31] We may have missed this effect due to the undifferentiated cohort of TBI patients used to assess deaths and the use of all-cause mortality. We also could not adjust mortality by age or injury severity.

For peer review only - http://bmjopen.bmj.com/site/about/guidelines.xhtml

Estimates of the impact of guidelines will depend on the extent to which the guidelines have been implemented. There are no national audit data on this, though one local audit conducted in 2001 indicated less than half of patients the SIGN guidelines deemed were safe for discharge from the ED were actually discharged.[32] Our study may therefore underestimate the potential impact of full implementation.

Comparison to previous literature:

A study that assessed the NICE head injury guideline's impact on inpatient hospital admissions using English data (although not using an interrupted time series) did not support predictions that increased CT imaging would lead to a reduction in hospital admissions.[13] Increased admissions following the introduction of the NICE guidelines could be due to increased diagnosis of TBI due to more CT imaging or the effect of the 4-hour ED target.[13] We found evidence that both factors increased hospital admissions in Scotland.

The 4-hour target's effect on adult head injury hospital admissions appears related to the time from admission which CT imaging is recommended . The 1st guideline contained no recommendation for when CT imaging should occur. The introduction of the 4- hour ED target increased head injury hospital admissions in both the 16-64 and ≥65 age groupings, presumably as patients were admitted to await imaging to comply with the target. The 2nd SIGN guideline increased the indications for immediate CT imaging in the 16-64 age group and was associated with a downward trend in admissions. A reduction in hospital admissions was not observed in the ≥65 age group following the 2nd SIGN guideline. The 2nd SIGN guideline includes a series of specific additional indications for imaging in the ≥65 age group that are recommended to occur within 8 hours of ED attendance. As 8 hours is longer than the 4-hour target patients with these indications for CT imaging would be admitted to hospital. This may account for why the 2nd SIGN guideline was not associated with reduced hospital admissions for those ≥65.

Analysis of English HES data from 2000-2011 found yearly paediatric hospital admissions for head injury increased from 34 to 37 inpatient admissions per 10 000 children.[33] We found Scottish paediatric admissions fell from 56 per 10 000 children in 1999 to 32 per 10 000 children in 2015. There is evidence that clinicians are less likely to implement head injury

For peer review only - http://bmjopen.bmj.com/site/about/guidelines.xhtml

guidelines in children.[33-35] A lack of implementation may explain why the SIGN guidelines were not associated with a reduction in paediatric hospital admissions.

Implications:

The overall effect of the SIGN guidelines was to reduce inpatient hospital admissions for head injured patients and this supports previous research indicating early CT imaging may represent a cost-effective management strategy.[9] However, the guidelines were associated with increased inpatient admissions for patients with TBI, possibly resulting from increased imaging identifying more TBI of lower clinical severity. Research better characterising the risk associated with TBI identified by CT imaging could help identify a sub-set of low-risk patients who could be safely discharged from the ED. This could help mitigate the increase in inpatient hospital admissions in this group associated with the SIGN and other similar guidelines.

We found evidence that the 4-hour ED target acted to reverse reductions in hospital admissions in the 16-64 age group associated with the SIGN guidelines. As has been previously argued, performance targets need to be carefully considered before implementation to ensure that they do not have unintended consequences, in this case undermining the benefits of evidence-based clinical guidelines.[36] A more granular approach to the 4-hour ED target that reflects condition specific clinical circumstances, such as the time frame of CT imaging in head injury, could help to prevent such costly unintended consequences.

Given the limitations in the mortality analysis undertaken it is hard to draw conclusions about how effective the SIGN guidelines were at reducing deaths from TBI. Future analysis should attempt to adjust for age and severity of injury and this will require patient level data.

Conclusion:

Increased early CT imaging of head injured patients may reduce hospital admissions. However, this effect may be offset by an increase in the diagnosis of TBI of lower severity and the 4-hour ED target. Future research should aim to better risk stratify patients with TBI identified by CT imaging to help reduce hospital admissions related to increased CT imaging.

For peer review only - http://bmjopen.bmj.com/site/about/guidelines.xhtml

Care should also be taken when introducing arbitrary performance targets, such as the 4-hour target, to ensure they do not undermine the beneficial effect of clinical guidelines.

Acknowledgements:

Sir Graham Teasdale commented on the manuscript and provided invaluable historical context and knowledge regarding the use of administrative data sets in traumatic brain injury research.

Competing interests:

The authors declare that they have no competing interests.

Funding:

Carl Marincowitz is funded by a National Institute for Health Research Doctoral Fellowship (DRF-2016-09-086). This study presents independent research funded by the National Institute for Health Research (NIHR). The views expressed are those of the author(s) and not necessarily those of the NHS, the NIHR or the Department of Health.

Authors' contributions:

This idea for the study was conceived by Carl Marincowitz with help from Trevor Sheldon, Fiona Lecky and Victoria Allgar. The analysis was completed by Carl Marincowitz and Eleanor Morris with specialist advice regarding interrupted time series analysis from Trevor Sheldon and Victoria Allgar. Fiona Lecky provided specialist advice regarding the clinical context and interpretation of the results.

Data Sharing:

Extra data is available by emailing Carl.Marincowitz@hyms.ac.uk.

References:

1. NICE. National Clinical Guidance Centre. (2014). CG 176 Head Injury Triage, assessment, investigation and early management of head injury in children, young people and adults. National Institute for Health and Care Excellence. In: NICE, ed. UK: DOH, 2014.
2. Early management of a patient with a head injury: a national clinical guideline. Edinburgh: SIGN 2009.
3. Miller JD. Minor, moderate and severe head injury. *Neurosurg Rev* 1986;9(1-2):135-9.
4. Kim J, Kemp S, Kullas K, et al. Injury patterns in patients who "talk and die". *J Clin Neurosci* 2013;20(12):1697-701. doi: 10.1016/j.jocn.2013.02.021

For peer review only - http://bmjopen.bmj.com/site/about/guidelines.xhtml

5. af Geijerstam JL, Oredsson S, Britton M, et al. Medical outcome after immediate computed tomography or admission for observation in patients with mild head injury: randomised controlled trial. *BMJ* 2006;333(7566):465. doi: 10.1136/bmj.38918.669317.4F
6. Stiell IG, Wells GA, Vandemheen K, et al. The Canadian CT Head Rule for patients with minor head injury. *Lancet* 2001;357(9266):1391-6.
7. Network SIGN. Guideline 46: Early Management of Patients with a Head Injury. 2000
8. Shivaji T, Lee A, Dougall N, et al. The epidemiology of hospital treated traumatic brain injury in Scotland. *BMC Neurology* 2014;14(1):2. doi: 10.1186/1471-2377-14-2
9. Pandor A, Goodacre S, Harnan S, et al. Diagnostic management strategies for adults and children with minor head injury: a systematic review and an economic evaluation. *Health Technol Assess* 2011;15(27):1-202. doi: 10.3310/hta15270
10. Dunning J, Daly JP, Malhotra R, et al. The implications of NICE guidelines on the management of children presenting with head injury. *Arch Dis Child* 2004;89(8):763-7. doi: 10.1136/adc.2003.042523
11. Hassan Z, Smith M, Littlewood S, et al. Head injuries: a study evaluating the impact of the NICE head injury guidelines. *Emerg Med J* 2005;22(12):845-9. doi: 10.1136/emj.2004.021717
12. Comptroller and Auditor General. Improving Emergency Care in England. *National Audit Office* 2004;HC 1075 Session 2003-2004
13. Goodacre S. Hospital admissions with head injury following publication of NICE guidance. *Emerg Med J* 2008;25(9):556-7. doi: 10.1136/emj.2007.055723
14. Wagner AK, Soumerai SB, Zhang F, et al. Segmented regression analysis of interrupted time series studies in medication use research. *J Clin Pharm Ther* 2002;27(4):299-309.
15. Lopez Bernal J, Cummins S, Gasparrini A. Interrupted time series regression for the evaluation of public health interventions: a tutorial. *Int J Epidemiol* 2016 doi: 10.1093/ije/dyw098 [published Online First: 2016/06/11]
16. http://apps.who.int/classifications/icd10/browse/2010/en [
17. National Clinical Coding Standards - OPCS-4 (2017) Reference Book.
18. http://www.isdscotland.org/Products-and-Services/Terminology-Services/Coding-and-Terminology-Systems/ [
19. Penfold RB, Zhang F. Use of interrupted time series analysis in evaluating health care quality improvements. *Acad Pediatr* 2013;13(6 Suppl):S38-44. doi: 10.1016/j.acap.2013.08.002 [published Online First: 2013/12/07]
20. Linden A. Conducting interrupted time-series analysis for single- and multiple-group comparisons. *The Stata Journal* 2015;15(2):480-500.
21. Hamilton I, Lloyd C, Hewitt C, et al. Effect of reclassification of cannabis on hospital admissions for cannabis psychosis: a time series analysis. *Int J Drug Policy* 2014;25(1):151-6. doi: 10.1016/j.drugpo.2013.05.016 [published Online First: 2013/07/23]
22. Lawrence T, Helmy A, Bouamra O, et al. Traumatic brain injury in England and Wales: prospective audit of epidemiology, complications and standardised mortality. *BMJ Open* 2016;6(11):e012197. doi: 10.1136/bmjopen-2016-012197
23. Rock DJ, Judd K, Hallmayer JF. The seasonal relationship between assault and homicide in England and Wales. *Injury* 2008;39(9):1047-53. doi: https://doi.org/10.1016/j.injury.2008.03.025
24. Chen AY, Colantonio A. Defining neurotrauma in administrative data using the International Classification of Diseases Tenth Revision. *Emerg Themes Epidemiol* 2011;8(1):4. doi: 10.1186/1742-7622-8-4 [published Online First: 2011/05/17]
25. Kontopantelis E, Doran T, Springate DA, et al. Regression based quasi-experimental approach when randomisation is not an option: interrupted time series analysis. *BMJ* 2015;350:h2750. doi: 10.1136/bmj.h2750

For peer review only - http://bmjopen.bmj.com/site/about/guidelines.xhtml

26. Barnighausen T, Tugwell P, Rottingen JA, et al. Quasi-experimental study designs series-paper 4: uses and value. *J Clin Epidemiol* 2017;89:21-29. doi: 10.1016/j.jclinepi.2017.03.012 [published Online First: 2017/04/04]
27. Hand DJ. Statistical challenges of administrative and transaction data. *J R Statist Soc A* 2018 (pre-publication presented at The Royal Statistical Society);181(Part 3):1-24.
28. Burns EM, Rigby E, Mamidanna R, et al. Systematic review of discharge coding accuracy. *Journal of Public Health* 2012;34(1):138-48. doi: 10.1093/pubmed/fdr054
29. Harley K, Jones C. Quality of Scottish Morbidity Record (SMR) data. *Health Bull (Edinb)* 1996;54(5):410-7. [published Online First: 1996/09/01]
30. Woodford TCAERGDSKFLISSTAWM. MAJOR TRAUMA IN OLDER PEOPLE. *The Traruma and Audit Research Network Report* 2017
31. Fuller G, Bouamra O, Woodford M, et al. Temporal trends in head injury outcomes from 2003 to 2009 in England and Wales. *Br J Neurosurg* 2011;25(3):414-21. doi: 10.3109/02688697.2011.570882
32. Kerr J, Smith R, Gray S, et al. An audit of clinical practice in the management of head injured patients following the introduction of the Scottish Intercollegiate Guidelines Network (SIGN) recommendations. *Emerg Med J* 2005;22(12):850-4. doi: 10.1136/emj.2004.022673 [published Online First: 2005/11/22]
33. Marlow R, Mytton J, Maconochie IK, et al. Trends in admission and death rates due to paediatric head injury in England, 2000-2011. *Arch Dis Child* 2015;100(12):1136-40. doi: 10.1136/archdischild-2015-308615
34. Plumb J. 'My 15-month-old child fell over and bumped his head doctor. He's fine now but he's been sick 3 times since it happened'. "Oh dear, that's not so NICE". *Br J Neurosurg* 2009;23(6):574; author reply 75. doi: 10.3109/02688690903419115 [published Online First: 2009/11/20]
35. Mooney JS, Yates A, Sellar L, et al. Emergency head injury imaging: implementing NICE 2007 in a tertiary neurosciences centre and a busy district general hospital. *Emerg Med J* 2011;28(9):778-82. doi: 10.1136/emj.2009.083360
36. Sheldon T. Promoting health care quality: what role performance indicators? *Qual Health Care* 1998;7 Suppl:S45-50. [published Online First: 1999/05/29]

For peer review only

Figure 1:

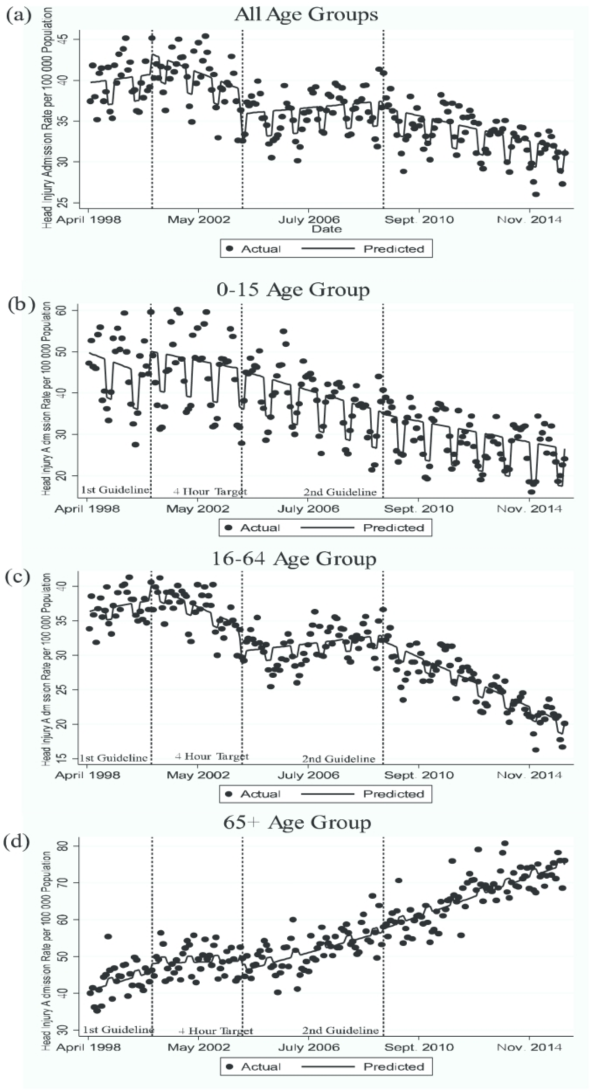

Figure 1: The Impact of the 4-hour target and SIGN guidelines on head injury admissions

210x246mm (300 x 300 DPI)

Figure 2:

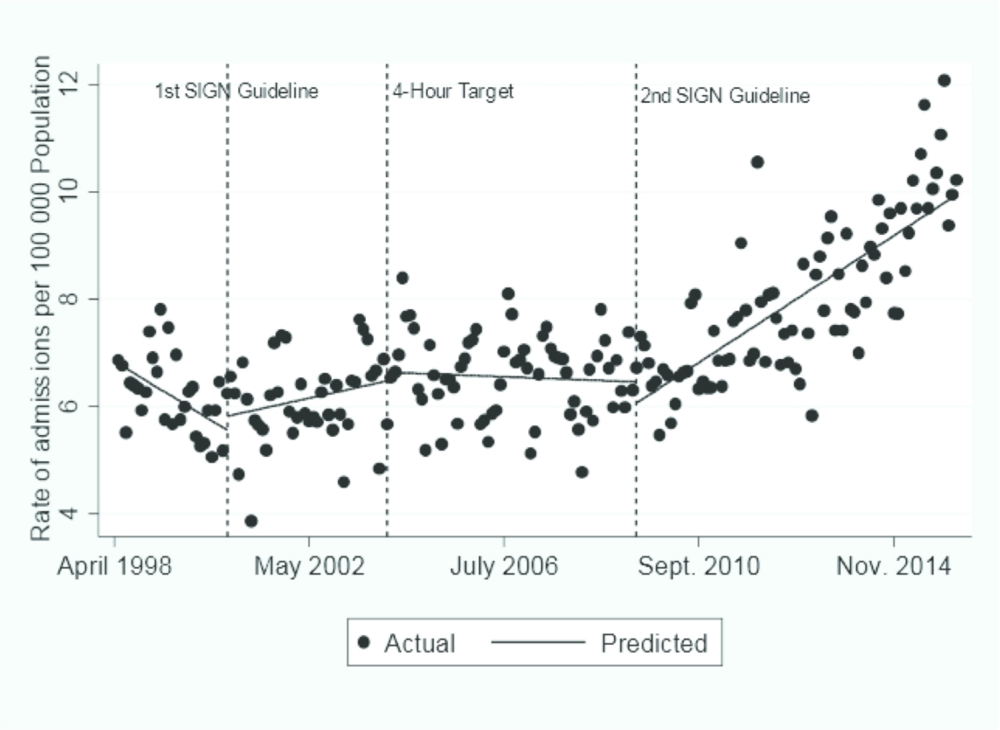

Figure 2: The impact of the SIGN guidelines and 4-Hour Target on admissions for Traumatic Brain Injury

206x159mm (300 x 300 DPI)

Supplementary Material 1: Summary of Health policies assessed

Policy	Time of Introduction	Key Features
1st SIGN Head Injury Guideline	August 2000	Indication for CT imaging (referenced directly from 2000 SIGN guideline):<br>(1) GCS<13<br>(2) A deteriorating level of consciousness or progressive focal neurological signs<br>(3) Confusion or drowsiness (CGS 13 or 14/15) followed by failure to improve within at most four hours of clinical observation<br>(4) Radiological/clinical evidence of a fracture, whatever the level of consciousness<br>(5) New focal neurological signs which are not getting worse<br>(6) Full consciousness (GCS 15/15) with no fracture but other features, including:<br>– severe and persistent headache<br>– nausea and vomiting<br>– irritability or altered behaviour<br>– a seizure.<br><br>Skull films should be carried out if any of the following apply and if CT is not being performed:<br>If the patient is alert and orientated and obeying commands (GCS 15/15) but:<br>– the mechanism of injury has not been trivial; or<br>– consciousness has been lost; or<br>– the patient has loss of memory or has vomited; or<br>– the scalp has a full thickness laceration or a boggy haematoma; or<br>– the history is inadequate.
4-Hour ED performance target	2004	98% of patients attending the ED to be assessed, treated and either discharged or admitted to hospital within 4 hours of arrival. Financial incentives associated with meeting the target.
2nd Sign Head Injury Guideline	May 2009	Referenced directly from 2nd SIGN Guidelines<br><br>Indications Immediate CT scanning (adult):<br>- GCS<13/15<br>- GCS 13/15 or 14/15 followed by failure to improve within one hour of clinical observation<br>- base of skull or depressed skull fracture and/or suspected penetrating injuries<br>- a deteriorating level of consciousness or new focal neurological signs<br>- GCS 15/15 with severe and persistent headache or two episodes of vomiting<br>- coagulopathy (eg warfarin use) and loss of consciousness or amnesia<br>Indications CT scanning within eight (adult):<br>- age>65 (with loss of consciousness or amnesia)

For peer review only

		- clinical evidence of a skull fracture but no clinical features indicative of an immediate CT scan<br>- any seizure activity<br>- significant retrograde amnesia (>30 minutes)<br>- dangerous mechanism of injury<br><br>Indications Immediate CT scanning (<16 years):<br>- GCS≤13 on assessment in ED<br>- witnessed loss of consciousness >5 minutes<br>- suspicion of open or depressed skull injury or tense fontanelle<br>- focal neurological deficit<br>- any sign of basal skull fracture.<br>Indications CT scanning within 8 hours (<16 years):<br>- presence of any bruise/swelling/laceration >5 cm on the head<br>- post-traumatic seizure<br>- amnesia (anterograde or retrograde) lasting >5 minutes<br>- clinical suspicion of non-accidental head injury<br>- a significant fall<br>- age under one year: GCS<15 in emergency department<br>- three or more discrete episodes of vomiting<br>- abnormal drowsiness.

For peer review only

Supplementary Material 2: Changes to Scotland's population structure

i) Mid-year Estimate Scottish Population 0-15

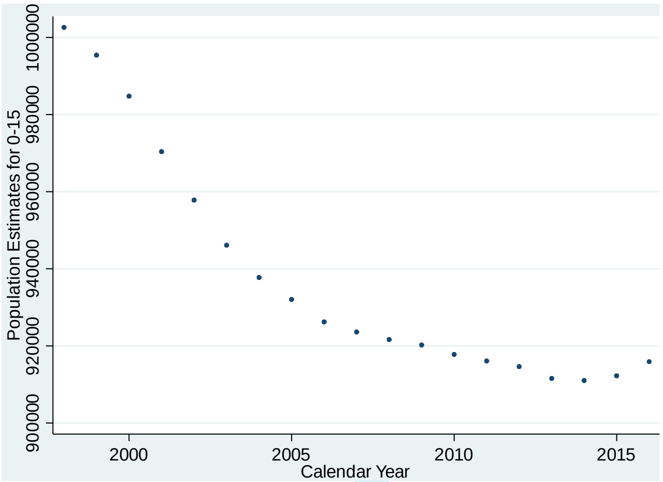

ii) Mid-year Estimate Scottish population 16-64

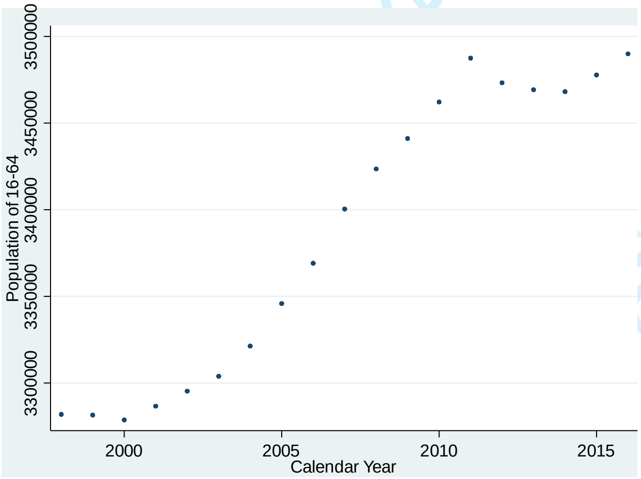

iii) Mid-year Estimate Scottish population 65+

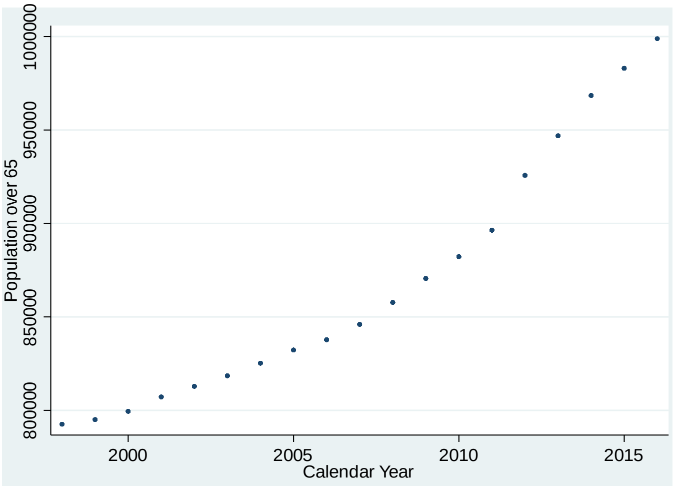

Supplementary Material 3: Increase in admissions related to the introduction of the 4-hour target in the 16-64 age group

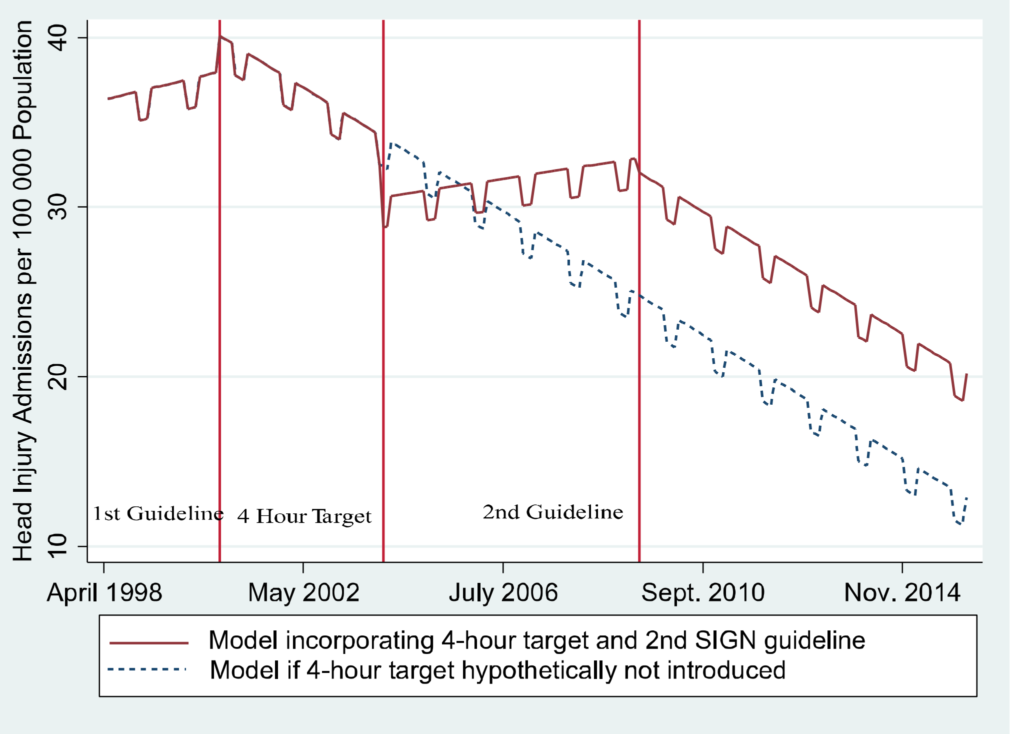

Supplementary Material 4: Estimate of Impact of the SIGN guidelines and introduction of 4-Hour Target admissions for head injury with 12-month time lag

Age Band	Winter	Initial Trend	1st SIGN Guideline	4-hour Target Introduced	2nd SIGN Guideline	Durbin-Watson Statistic
**All ages**	-3.00 (95% CI: -3.75 to -2.26) P<0.01	0.04 (95% CI: -0.07 to 0.14) P=0.48	**Change level:** 2.66 (95% CI:0.27 to 5.04) P=0.03	**Change level:** -2.73 (95% CI:-4.85 to -0.61) P<0.01	**Change level:** -2.07 (95% CI:-3.74 to -0.40) P=0.02	Untransformed 1.77 Prais-Winsten 2.00
			**Change trend:** -0.23 (95% CI:-0.38 to -0.08) P<0.01	**Change trend:** 0.25 (95% CI: 0.14 to 0.36) P<0.01	**Change trend:** -0.13 (95% CI: -0.18 to -0.08) P<0.01	
**0-15**	-9.31 (95% CI: -11.05 to -7.59) P<0.01	-0.18 (95% CI:-4.89 to 0.13) P=0.26	**Change level:** 8.53 (95% CI:1.93 to 15.13) P=0.01	**Change level:** 1.61 (95% CI:-4.39 to 7.61) P=0.60	**Change level:** -1.71 (95% CI:-6.61 to 3.20) P=0.49	Untransformed 1.37 Prais-Winsten 1.85
			**Change trend:** -0.17 (95% CI:-0.61 to 0.28) P=0.47	**Change trend:** 0.15 (95% CI: -0.17 to 0.48) P=0.36	**Change trend:** 0.10 (95% CI:-0.50 to 0.26) P=0.18	
**16-64**	-1.80 (95% CI:-2.56 to -1.04) P<0.01	0.06 (95% CI:-0.06 to 0.17) P=0.34	**Change level:** 1.29 (95% CI:-1.28 to 3.85) P=0.32	**Change level:** -4.19 (95% CI:-6.48 to -1.89) P<0.01	**Change level:** -3.06 (95% CI:-4.88 to -1.24) P<0.01	Untransformed 1.57 Prais-Winsten 2.05
			**Change trend:** -0.25 (95% CI:-0.41 to -0.09) P<0.01	**Change trend:** 0.29 (95% CI: -0.17 to 0.40) P<0.01	**Change trend:** -0.25 (95% CI:-0.30 to -0.19) P<0.01	
**65+**	1.69 (95% CI: 0.22 to 3.17) P=0.03	0.17 (95% CI:-0.04 to 0.38) P= 0.10	**Change level:** 3.64 (95% CI:-1.08 to 8.36) P= 0.13	**Change level:** -1.64 (95% CI:-5.83 to 2.55) P=0.44	**Change level:** 2.30 (95% CI:-1.00 to 5.60) P=0.17	Untransformed 1.78 Prais-Winsten 2.00
			**Change trend:** -0.22 (95% CI:-0.51 to 0.07) P=0.14	**Change trend** 0.26 (95% CI: 0.04 to 0.48) P=0.02	**Change trend:** 0.003 (95% CI:-0.10 to 0.10) P=0.95	

For peer review only

Supplementary Material 5: Increase in admissions related to the introduction of the 2nd SIGN guideline

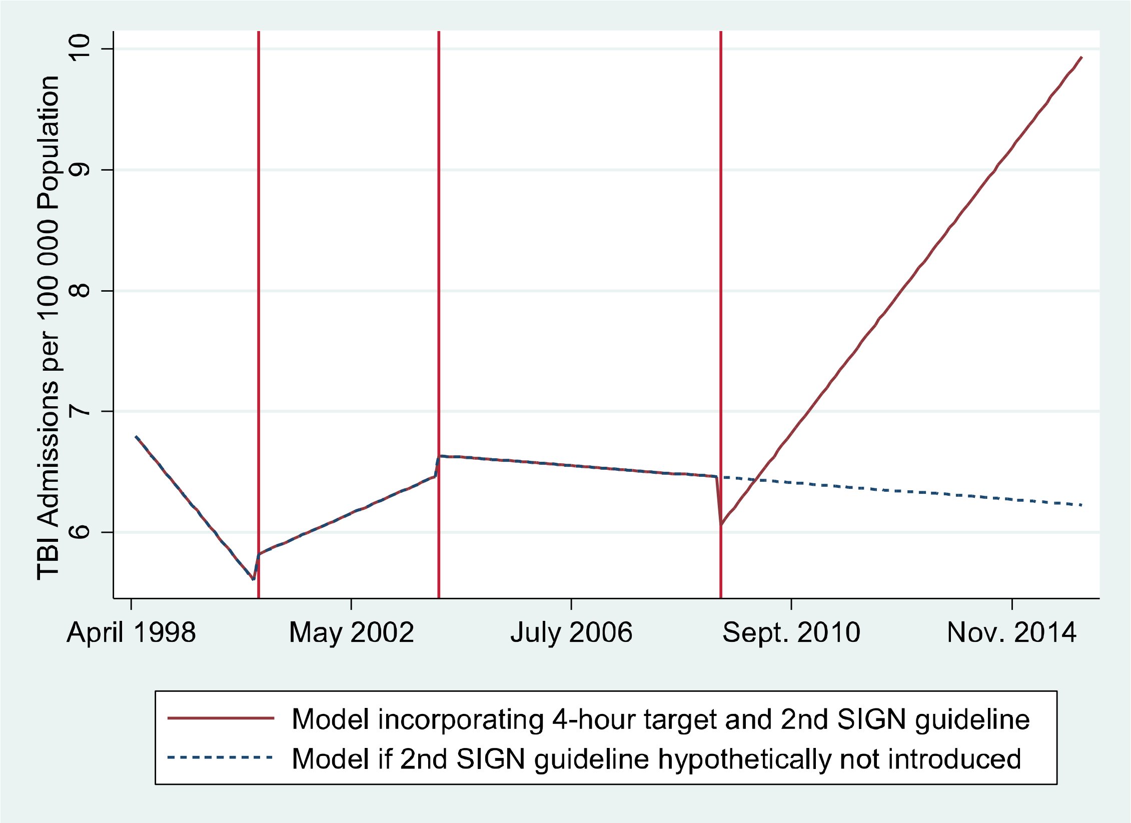

Supplementary Material 6: The impact of the SIGN guidelines on the percentage of admissions for TBI that resulted in neurosurgery

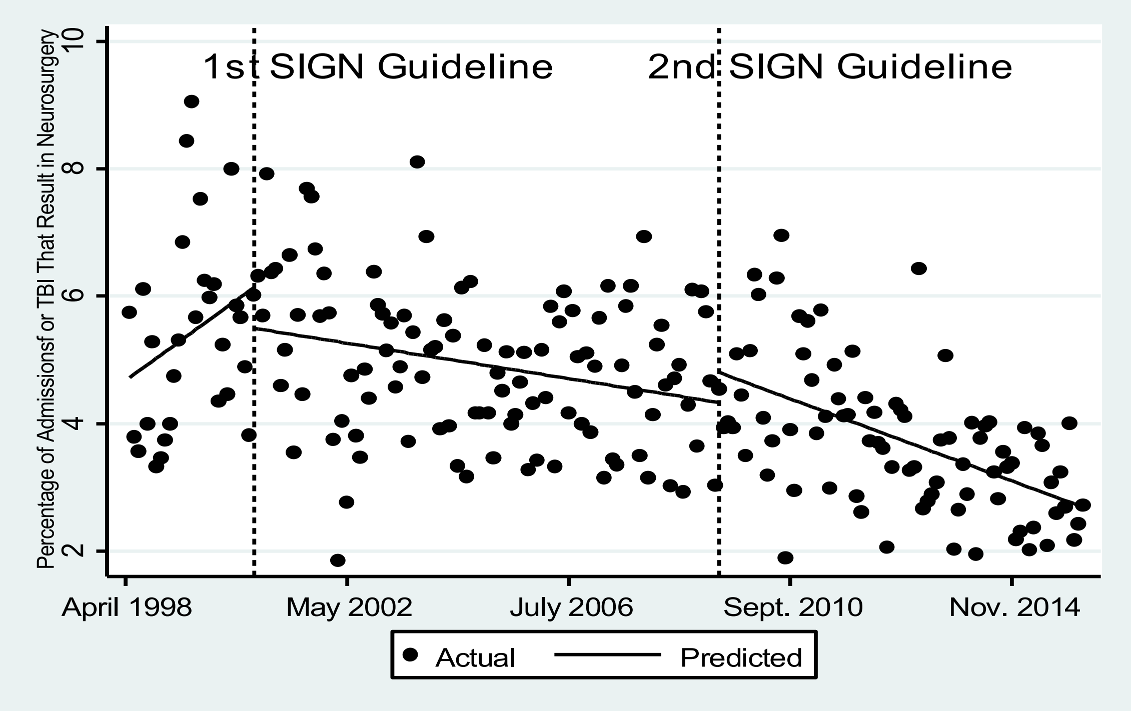

Supplementary Material 7: Estimate of impact of the SIGN guidelines and 4-Hour Target on admissions for Traumatic Brain Injury with 12-month time lag

**Outcome**	**Initial Trend**	**1st SIGN Guideline**	**4-hour Target Introduced**	**2nd SIGN Guideline**	**Durbin-Watson Statistic**
**Admissions for TBI/ 100 000**	-0.05 (95% CI: -0.10 to 0.003) P=0.07	**Change level:** 0.75 (95% CI:-0.34 to 1.84) P=0.18	**Change level:** 0.35 (95% CI:-0.63 to 1.33) P=0.48	**Change level:** -0.16 (95% CI: -0.92 to 0.60) P=0.69	Untransformed 1.44<br>Prais-Winsten 2.03
		**Change trend:** 0.05 (95% CI:-0.02 to 0.12) P=0.16	**Change trend:** -0.01 (95% CI:-0.06 to 0.04) P=0.66	**Change trend:** 0.05 (95% CI:3 to 0.08) P<0.01	
**Percentage TBI admissions neurosurgical**	0.05 (95% CI: -0.01 to 0.11) P=0.10	**Change level:** -0.96 (95% CI:-2.19 to 0.28) P=0.13		**Change level:** 0.17 (95% CI:-0.59 to 0.93) P=0.66	Untransformed 1.85
		**Change trend:** -0.06 (95% CI:-0.12 to 0.003) P=0.06		**Change trend:** -0.02 (95% CI:-0.04 to-0.004) P=0.01	

For peer review only

Supplementary Material 8: The impact of the SIGN guidelines on deaths per 100 000 population within 30 days of admission with traumatic brain injury

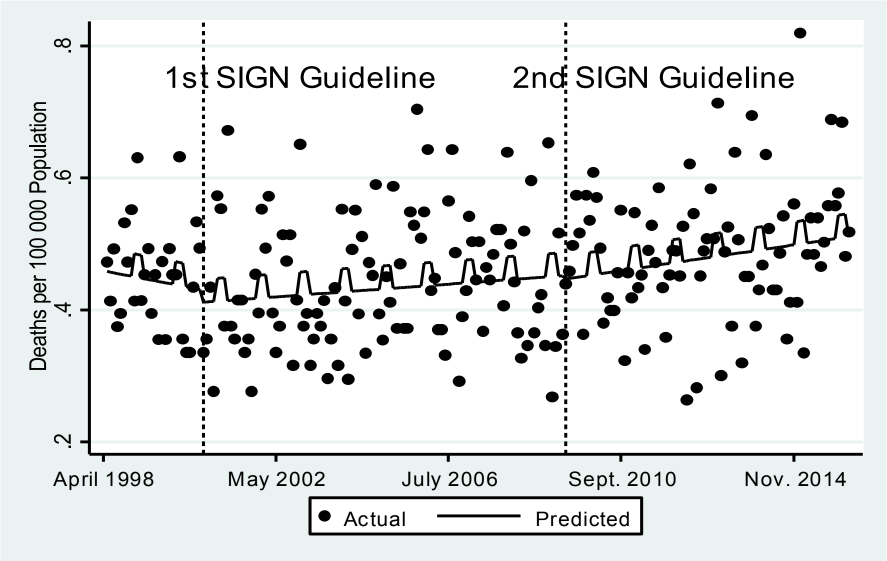

Supplementary Material 9: The impact of the SIGN guidelines on the percentage of patients admitted with TBI who died within 30 days of admission

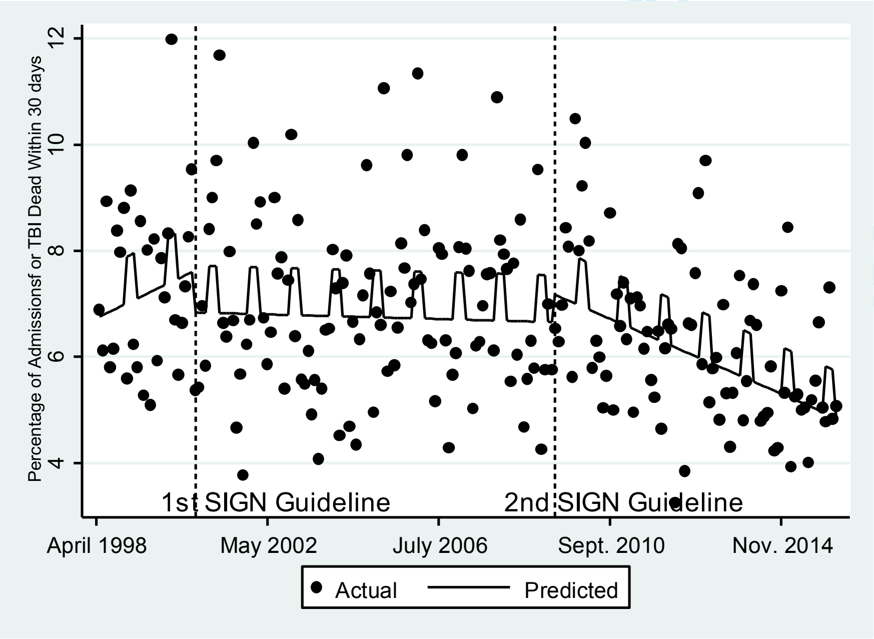

Supplementary Material 10: Estimate of impact of the SIGN guidelines on deaths following admission for Traumatic Brain Injury with 12 month time lag

Outcome	Winter	Initial Trend	1st SIGN Guideline	2nd SIGN Guideline	Durbin-Watson Statistic
Deaths/100 000	0.03 (95% CI:-0.01 to 0.06) P=0.15	-0.001 (95% CI:-0.004 to 0.003) P=0.59	**Change level:** -0.02 (95% CI:- 0.09 to 0.06) P=0.66	**Change level:** -0.03 (95% CI:-0.08 to 0.03) P=0.32	Untransformed 2.29
			**Change trend:** 0.001 (95% CI:-0.002 to 0.005) P=0.44	**Change trend:** 0.001 (95% CI:-0.0002 to 0.002) P=0.10	
Percentage TBI admissions death	0.74 (95% CI: 0.18 to 1.30) P=0.10	0.03 (95% CI:-0.03 to 0.10) P=0.34	**Change level:** -1.06 (95% CI: -2.40 to 0.29) P=0.12	**Change level:** -0.37 (95% CI:-1.20 to 0.47) P=0.39	Untransformed 2.17
			**Change trend:** -0.03 (95% CI:-0.10 to 0.04) P=0.38	**Change trend:** -0.02 (95% CI:-0.04 to -0.004) P=0.02	

For peer review only

BMJ Open

The impact of the SIGN head injury guidelines and NHS 4-hour Emergency Target on hospital admissions for head injury in Scotland: An Interrupted Times Series.

Journal:	*BMJ Open*
Manuscript ID	bmjopen-2018-022279.R1
Article Type:	Research
Date Submitted by the Author:	10-Jul-2018
Complete List of Authors:	Marincowitz, Carl; University of Hull, Hull York Medical School<br>Lecky, Fiona; University of Sheffield, School of Health and Related Research<br>Morris, Eleanor ; Hull York Medical School<br>Allgar, Victoria; Hull York Medical School<br>Sheldon, Trevor; University of York, Health Sciences
**Primary Subject Heading**:	Emergency medicine
Secondary Subject Heading:	Health policy, Radiology and imaging, Diagnostics
Keywords:	Traumatic Brain Injury, Head Injury, SIGN Guidelines, NHS 4-hour Emergency Department Performance Target, Interrupted Time Series

SCHOLARONE™
Manuscripts

The impact of the SIGN head injury guidelines and NHS 4-hour Emergency Target on hospital admissions for head injury in Scotland: An Interrupted Times Series.

Carl Marincowitz[1] MB BChir, MSc, BA

Fiona E. Lecky[2] MB Ch B, FRCS, DA, MSc, PhD, FCEM

Eleanor Morris[3]

Victoria Allgar[4] PhD, Chartered Statistician, Royal Statistical Society, BSc

Trevor A. Sheldon[5] MSc, MSc, DSc, FMedSci

1. **Corresponding Author.** Hull York Medical School, Allam Medical Building, University of Hull, Hull HU6, UK 7RX, Fax: +44 (0) 1482 464705 Tel +44 (0) 870 1245500
Email: carl.marincowitz@hyms.ac.uk

2. School of Health and Related Research, University of Sheffield, Regent Court, 30 Regent Street, Sheffield, S1 4DA, UK, Fax: +44 (0)114 222 0749 Tel: (+44) (0)114 222 4345,
Email: f.e.lecky@sheffield.ac.uk

3. Hull York Medical School, Allam Medical Building, University of Hull, Hull HU6, UK 7RX, Fax: +44 (0) 1482 464705 Tel +44 (0) 870 1245500 Email: hyem7@hyms.ac.uk

4 Hull York Medical School, John Hughlings Jackson Building, University of York, Heslington, York YO10 5DD Tel: 0870 124 5500, Email: Victoria.Allgar@hyms.ac.uk

5. Department of Health Sciences, University of York, Alcuin Research Resource Centre, Heslington, York, YO10 5DD, Tel +44 (0) 1904 321344, Fax: +44 (0) 1904 323 521,
E-mail: trevor.sheldon@york.ac.uk

Word Count: 4001

For peer review only

For peer review only - http://bmjopen.bmj.com/site/about/guidelines.xhtml

Abstract

Objectives:

Head injury is a common reason for Emergency Department (ED) attendance. Around 1% of patients have life-threatening injuries, whilst 80% of patients are discharged. National guidelines (SIGN) were introduced in Scotland with the aim of achieving early identification of those with acute intracranial lesions yet safely reducing hospital admissions.

This study aims to assess the impact of these guidelines and any effect the national 4-hour ED performance targets had on hospital admissions for head injury.

Setting:

All Scottish hospitals between April 1998 and March 2016.

Participants:

Patients admitted to hospital for head injury or traumatic brain injury (TBI) diagnosed by CT imaging identified using administrative Scottish Information Services Division data. There are 275 hospitals in Scotland. In 2015/2016 there were 571, 221 emergency hospital admissions in Scotland.

Interventions:

The SIGN head injury guidelines introduced in 2000 and 2009. The 4-hour ED target introduced in 2004.

Outcomes:

The monthly rate of hospital admissions for head injury and traumatic brain injury.

Study Design:

An interrupted time series analysis.

Results:

The 1st guideline was associated with a reduction in monthly admissions of 0.14 (95% CI:0.09 to 4.83) per 100, 000 population. The 4-hour target was associated with a monthly increase in admissions of 0.13 (95% CI:0.06 to 0.20) per 100, 000 population. The 2nd guideline reduced monthly admissions by 0.09 (95% CI:-0.13 to -0.05) per 100, 000 population. These effects varied between age groups.

The guidelines were associated with increased admissions for patients with injuries identified by CT imaging- Guideline 1: 0.06 (95% CI: 0.004 to 0.12); Guideline 2: 0.05 (95% CI: 0.04 to 0.06) per 100 000 population.

Conclusion:

Increased CT imaging of head injured patients recommended by SIGN guidelines reduced hospital admissions. The 4-hour ED target and the increased identification of TBI by CT imaging acted to undermine this effect.

For peer review only - http://bmjopen.bmj.com/site/about/guidelines.xhtml

Strengths and limitations of this study:

This is the first study to assess the impact of the SIGN head injury guidelines and 4-hour Emergency Department target on hospital admissions for head injury.

We used the robust method of interrupted time series analysis and found the SIGN guidelines acted to reduce hospital admissions, but the 4-hour target increased hospital admissions.

Due to the aggregated nature of the available data we were unable to perform some age group specific and injury sub-group sensitivity analysis.

Keywords:

Traumatic Brain Injury, Head Injury, SIGN Guidelines, NHS 4-hour Emergency Department Performance Target and Interrupted Time Series.

For peer review only

For peer review only - http://bmjopen.bmj.com/site/about/guidelines.xhtml

Background:

There are 1.4 million annual attendances to Emergency Departments (ED) in England and Wales following a head injury (blunt trauma to the head).[1] In Scotland an estimated 6.6% of ED attendances are for head injury.[2] Approximately 95% of patients present with an initial Glasgow Coma Scale (GCS) of 13-15, indicating normal or minimallyimpaired conscious level and are defined as having a minor head injury.[1 3]

Around 1% of minor head injured patients have life-threatening traumatic brain injuries (TBI) (injury to the brain/ functional impairment due to external force) but this may not be initially clinically apparent.[1] Early identification of severe TBI can facilitate life-saving neurosurgery.[4] The clinical challenge is to differentiate patients with life-threatening TBI who present with a high conscious level from patients who can be discharged safely. This can be achieved through observation for deterioration or cranial CT imaging.[5] The health services challenge is finding a way of differentiating these groups in a way that minimises unnecessary imaging or inpatient hospital admissions of patients without clinically important TBI. Research has focused on developing clinical decision rules that, using clinical assessment, triage patients at risk of life-threatening TBI to CT imaging and allow the discharge of low-risk patients. The most validated, in adults, is the Canadian CT Head Rule (CCHR) and this forms the basis of the Scottish Intercollegiate Guidelines Network (SIGN) and English National Institute for Health and Care Excellence (NICE) head injury guidelines.[1 2 6] The 2nd SIGN guideline contains specific paediatric indications for CT imaging influenced by the CHALICE rule, which was derived in a population of head injured children.[2]

Two iterations of the SIGN guidelines have been introduced (Fig.1).[2 7] The first recommended increased CT imaging of head injured patients but still featured a role for skull x-rays and admission for observation.[7] The 2nd SIGN guideline extended CT imaging further.[2] A study assessing trends in hospital admissions for TBI in Scotland between 1998 and 2009 found changes in rates of admissions at specific time points after 2000, but the analysis was data driven and does not explicitly assess the impact of the SIGN guidelines.[8] Implementation studies of NICE head injury guidelines in England suggested that the cost of increased CT imaging (£100 per scan) would be offset by a reduction in inpatient hospital admissions (£847 per admission) as patients admitted for observation would be dicharged from the ED following normal CT imaging, preventing many admissions.[9-11]

For peer review only - http://bmjopen.bmj.com/site/about/guidelines.xhtml

However, a study using English Hospital Episodes Statistics (HES) found head injury inpatient admissions increased following the introduction of the NICE guidelines, possibly due to the near simultaneous introduction of the 4-hour ED target. Supplementary material 1 presents the details of the 4-target and in 2002 around 23% of all patients in the UK remained in the ED for longer than 4-hours.[12] The target could act to incentivise inpatient admissions for patients at medium risk for TBI, who require imaging within 8 hours, to avoid breaches. Previously they would have been imaged in the ED and the majority discharged. A further cause of increased admissions could be the detection of brain injuries previously not identified, some without clinically significant sequelae, due to increased imaging.[13] In Scotland the 4-hour target was not introduced at the same time as the SIGN guidelines. This provides a unique opportunity to assess the independent effects of these policy interventions.

Aims:

This study aims to assess the impact of the introduction of the SIGN head injury guidelines and the 4-hour target on the rate of hospital admissions in patients with head injury and explores the extent to which any increase in admissions were due to the identification of more TBI.

Methods:

Study Design:

Interrupted time series analysis is an established and robust method for the evaluation of health policies implemented at discrete time points.[14 15]

Dataset:

The Scottish Information Services Division (ISD) contains a repository of information routinely collected at discharge from hospital for all non-obstetric and non-psychiatric inpatient hospital admissions in Scotland (including most short stay admissions to Clinical Decision Units) . Since 1996 reason for admission has been categorised using ICD10 diagnostic coding and since 1989 interventions have been coded using the OPSC4 classification.[16-18] This repository was used, for the period 1998-2016, to generate monthly numbers of: patients admitted with ICD10 coding for head injury, patients admitted with

For peer review only

ICD coding indicating TBI identified by CT imaging, neurosurgical interventions and deaths within 30 days of admission for patients with CT evidence of TBI. Diagnostic codes were counted if they were either primary or secondary diagnoses.

The data extract used for analysis is available from ISD Scotland for a commercial fee. The data set was fully anonymised and aggregated with small numbers suppressed.

We selected an ICD10 code subset definition of head injury that includes all possible definitions of TBI and head injury (Table 1). The ICD10 codes used are consistent with those selected to explore the NICE guideline's effect on hospital admissions for head injury, with the addition of codes for crush injuries.[13] We selected an ICD10 code subset to define TBI that corresponds to injuries identified by CT imaging including codes for traumatic intra-cranial haemorrhages and skull fractures, but excluded codes for concussion and other clinical diagnoses (Table 1).

Outcomes:

Admissions for head injury:

The monthly number of patients admitted to hospital with one or more ICD10 code indicating head injury between April 1998 and March 2016 was generated by the ISD. As the SIGN guidelines have specific recommendations regarding paediatric patients and patients 65 and over, admissions were stratified into: 0-15, 16-64 and ≥65 age groupings. Yearly and monthly number of admissions were converted into a rate per 100, 000 population using Nomis Office of National Statistics (ONS) mid-year population estimates for Scotland for each age grouping (Supplementary Material 2).

Admissions for Traumatic Brain Injury:

The ISD generated the monthly number of patients admitted to hospital with at least one ICD10 code sub-classification indicting an admission for TBI. Monthly and yearly rates per 100, 000 population of admissions for TBI were calculated using Nomis ONS mid-year Scottish population estimates.

The ISD also provided the monthly number of patients admitted with an ICD10 code indicating TBI that had one or more OPSC4 neurosurgical codes (Table 2). A monthly proportion of admissions for TBI that resulted in neurosurgery was estimated. The small number of monthly TBI admissions prevented release of data stratified by age group.

For peer review only - http://bmjopen.bmj.com/site/about/guidelines.xhtml

Table 1: Annual trends admissions for head injury and TBI

Year	Annual rates head injury admissions per 100, 000 population each age group.*				Annual rates TBI admissions per 100, 000 population all age.** (Percentage annual rate all head injury admissions)
	All ages	0-15	16-64	65+	
1999	484	558	453	536	77 (15.9%)
2000	476	519	449	556	70 (14.7%)
2001	493	561	457	578	72 (14.6%)
2002	489	556	447	598	71 (14.5%)
2003	451	521	404	581	76 (16.9%)
2004	435	495	381	589	82 (18.9%)
2005	417	497	350	591	78 (18.7%)
2006	427	477	369	585	79 (18.5%)
2007	442	441	393	653	79 (17.9%)
2008	435	400	386	670	76 (17.4%)
2009	440	406	377	709	79 (18%)
2010	400	361	337	709	80 (20%)
2011	421	369	352	741	91 (21.6%)
2012	411	339	321	817	89 (21.7%)
2013	396	307	293	845	97 (24.5%)
2014	385	319	268	848	102 (26.5%)
2015	373	316	251	867	123 (33%)

For peer review only

*ICD10 codes for head injury: S00-S09, T04.0 and T06.0

** ICD10 codes for TBI: S02.0, S02.1, S02.7, S02.8, S02.9, S06.1, S06.3, S06.4, S06.5, S06.6, S06.7, S06.8, S06.9, T04.0 and T06.0

For peer review only - http://bmjopen.bmj.com/site/about/guidelines.xhtml

Table 2: Impact of the SIGN guidelines on number of admissions and deaths from Traumatic Brain Injury per 100, 000 Scottish population

Outcome	Winter Effect	Initial Trend	1st SIGN Guideline	4-hour Target Introduced	2nd SIGN Guideline	Durbin-Watson Statistic
**Admissions for TBI/100 000**		-0.04 (95% CI: -0.09 to 0.004) P=0.07	**Change level:** 0.26 (95% CI:- 0.74 to 1.26) P=0.61	**Change level:** 0.16 (95% CI: -0.67 to 0.99) P=0.71	**Change level:** -0.39 (95% CI: -1.09 to 0.30) P=0.27	Untransformed 1.46 Prais-Winsten 2.02
			**Change trend:** 0.06 (95% CI: 0.004 to 0.12) P=0.04	**Change trend:** -0.02 (95% CI: -0.05 to 0.01) P=0.24	**Change trend:** 0.05 (95% CI: 0.03 to 0.07) P<0.01	
**Percentage TBI admissions neurosurgical+**		0.05 (95% CI: -0.01 to 0.11) P=0.10	**Change level:** -0.64 (95% CI: -1.83 to 0.56) P=0.29		**Change level:** 0.47 (95% CI:-0.17 to 1.12) P=0.15	Untransformed 1.81
			**Change trend:** -0.06 (95% CI: -0.12 to 0.001) P=0.047		**Change trend:** -0.01 (95% CI:-0.03 to -0.003) P=0.01	
**Deaths/100 000**	0.03 (95% CI: 0.001 to 0.07) P=0.04	-0.001 (95% CI:-0.004 to 0.002) P=0.57	**Change level:** -0.02 (95% CI:- 0.09 to 0.06) P=0.62		**Change level:** -0.01 (95% CI:-0.06 to 0.05) P=0.85	Untransformed 2.3
			**Change trend:** 0.001 (95% CI:-0.002 to 0.005) P=0.44		**Change trend:** 0.0004 (95% CI: -0.001 to 0.001) P=0.46	
**Percentage TBI admissions death**	0.88 (95% CI: 0.36 to 1.41) P<0.01	0.03 (95% CI: -0.03 to 0.10) P=0.35	**Change level:** -0.79 (95% CI: -2.12 to 0.54) P=0.24		**Change level:** 0.52 (95% CI: -0.32 to 1.35) P=0.22	Untransformed 2.08
			**Change trend:** -0.03 (95% CI: -0.10 to 0.03) P=0.33		**Change trend:** -0.03 (95% CI: -0.04 to -0.01) P<0.01	

+ Neurosurgical procedure defined as 1 or more OPSC4 codes: A05.2, A05.3, A05.4, A05.8, A05.9, A40.1, A40.8, A40.9, A41.1, A41.8, A41.9, V03.1, V03.2, V03.4, V03.6, V03.7, V03.8, V03.9, V05.3 and V05.4

For peer review only - http://bmjopen.bmj.com/site/about/guidelines.xhtml

Deaths related to Traumatic Brain Injury:

The ISD provided the monthly number of patients who died within 30 days of admission with at least one ICD10 code for TBI. This was converted into a rate using Nomis ONS mid-year Scottish population estimates. The monthly proportion of patients admitted with TBI who died within 30 days of admission was estimated using the total number of monthly admissions for ICD10 codes that corresponded to TBI.

Statistical analysis:

A monthly time series of the rate of inpatient hospital admissions for head injury ICD codes was plotted from April 1998 to March 2016. An interrupted times series analysis was completed assessing the impact of SIGN head injury guidelines and the 4-hour ED target using the ITSA package in STATA 14.[19 20] The model included 3 intervention time points: the introduction of the 1st SIGN guideline in August 2000, the introduction of the 4-hour target in 2004 and the introduction of the 2nd SIGN guideline in May 2009. Analysis was stratified into 3 age groups: 0-15, 16-64 and ≥65s. A segmented regression model predicting the rate of hospital admissions per 100, 000 population in each age grouping per month was estimated.[15]

Autocorrelation of the residuals was assessed using the Durbin-Watson and Rho statistic.[15 21] Where there was sufficient deviation from a Durbin Watson statistic of 2 and the Rho statistic was not statistically significant, the Prais-Winsten transformation was used to adjust for auto-correlation.[15] Seasonality was assessed by introducing a dummy variable to the model in which winter months (December, January and February) were coded 1 and was included in the model when a statistically significant predictor.

The interrupted time series analysis was repeated to assess: the impact of the SIGN guidelines and 4-hour target on the rate of hospital admissions for patients with an ICD10 code indicating TBI, the impact of SIGN guidelines on the proportion of inpatient admissions for TBI that resulted in neurosurgery or death and the death rate within 30 days of admission for TBI. It was thought a priori that the 4-hour target would not plausibly have affected neurosurgery or deaths. This was confirmed through visual inspection and was excluded from analysis of these outcomes.

For peer review only - http://bmjopen.bmj.com/site/about/guidelines.xhtml

Estimates of the impact of the policy interventions on inpatient admissions were made by using the pre-intervention model to estimate hypothetical monthly rates of admissions if no intervention had occurred. These were subtracted from the monthly admission rates estimated by the post-intervention model.[14] To explore any effect of a policy implementation lag a sensitivity analysis was performed for all the models in which the 12 months immediately following the introduction of a policy change, were excluded from analysis.[15]

Patient and Public Involvement

The Hull and East Yorkshire NHS Trust Trans-Humber Consumer Research Panel and Hull branch of the Headway charity were consulted in the initial stages of developing the research questions addressed in this study. These patient groups highlighted that although national head injury guidelines seemed evidence based, there appeared to be little evidence to show they had achieved their aims.

Results:

Head injury inpatient hospital admissions:

Table 1 show yearly rates of inpatient hospital admissions. Fig. 1 and Table 3 present the results of the interrupted time series assessing the impact of the SIGN guidelines and 4-hour ED target on monthly head injury admissions. Admission rates and estimates of effect are reported per 100, 000 population in each age grouping. The SIGN guidelines and 4-hour target were associated with a significant change in the total level and trend of the rate admissions for head injury (Fig 1a). The effect varied between age group.

0-15 Age Group:

Monthly inpatient admissions fell from 47.18 to 24.02 per 100, 000 per month over the time period (Fig. 1b). Neither SIGN guideline nor the introduction of the 4-hour target significantly affected the underlying reducing trend in hospital admissions but the first guideline was associated with a borderline statistically significant increase in level (6.06; 95% CI: -0.49 to 12.62) (Table 3). Admissions were less likely to occur in winter months (-9.19; 95% CI: -10.81 to -7.57) (Table 3).

16-64 Age Group:

For peer review only - http://bmjopen.bmj.com/site/about/guidelines.xhtml

Inpatient admissions fell from 36.39 to 20.20 per 100, 000 per month from April 1998 to March 2016 (Fig 1c). Before the 1st SIGN guideline hospital admissions were increasing monthly (0.04; 95% CI: -0.08 to 0.15) (Table 3 and Fig. 1c). The 1st guideline was associated with a declining monthly trend in admissions (-0.20; 95% CI:-0.35 to -0.05). The 4-hour target was associated with an initial fall in the number of inpatient admissions (-3.54; 95% CI: -5.76 to -1.33) but subsequent trend of increasing monthly admissions (0.18; 95% CI: 0.10 to 0.27). The 2nd guideline was associated with a return to a declining trend in admissions (-0.18; 95% CI:-0.23 to -0.13). Inpatient admissions were reduced in winter months (-1.74; 95% CI:-2.48 to -1.01).

The trend following the introduction of the 1st SIGN guideline hypothetically continued in the period after the introduction of the 4-hour target is shown in Supplementary Material 3. By subtracting this from the model that incorporated the introduction of the 4-hour target and the 2nd SIGN guideline we estimate that from January 2004 to March 2016 the introduction of the 4-hour target was associated with an additional 745 hospital admissions per 100 000 population aged 16-64.

≥65 Age Group:

Monthly admissions increased from 40.00 to 76.09 over the time period (Fig. 1d). The only statistically significant change in the underlying trend was at the introduction of the 4-hour target which was associated with an acceleration in the increase in hospital admissions (0.15; 95% CI: 0.01 to 0.28) (Table 3 and Fig. 1d). Winter months were associated with increased hospital admissions (1.67; 95% CI: 0.32 to 3.02).

Sensitivity Analysis for 12-month implementation lag:

The introduction of an intervention lag in the model (Supplementary Material 4) did not materially affect estimates of effect associated with interventions.

Inpatient hospital admissions for Traumatic Brain Injury on CT scan:

Admission per 100, 000 per month increased from 6.85 to 10.21 over the time period (Fig. 2). Before the 1st SIGN guideline hospital admissions were decreasing (-0.04; 95% CI:-0.09 to 0.004) (Table 2 and Fig. 2). The introduction of the 1st guideline was associated with a trend of increasing admissions (0.06; 95% CI: 0.004 to 0.12). The 4-hour target was not associated

For peer review only - http://bmjopen.bmj.com/site/about/guidelines.xhtml

For peer review only

with a significant change in level or trend. The introduction of the 2nd SIGN guideline was associated with an acceleration in the increase in admissions (0.05; 95% CI: 0.04 to 0.06).

Comparing admissions for TBI that would have occurred if the 2nd SIGN guideline had not been introduced with the empirically derived model indicates that from May 2009 to March 2016 the introduction of the 2nd SIGN guideline was associated with an additional 138 hospital admissions per 100, 000 population (Supplementary Material 5).

For peer review only

For peer review only - http://bmjopen.bmj.com/site/about/guidelines.xhtml

Table 3: Impact of the SIGN guidelines and introduction of 4-Hour ED Target on number of Head Injury Admissions per 100, 000 Scottish population by age group

**Age Band**	**Winter Effect**	**Initial Trend**	**1st SIGN Guideline**	**4-hour Target Introduced**	**2nd SIGN Guideline**	**Durbin-Watson Statistic**
**All ages**	-3.00 (95% CI:-3.78 to -2.30) P<0.01	0.04 (95% CI: -0.08 to 0.15) P=0.53	**Change level:** 2.45 (95% CI:0.09 to 4.83) P=0.04	**Change level:** -2.89 (95% CI:-4.84 to -0.95) P<0.01	**Change level:** -0.84 (95% CI:-2.50 to 0.78) P=0.31	Untransformed 1.68 Prais-Winsten 2.02
			**Change trend:** -0.14 (95% CI: -0.27 to -0.01) P=0.03	**Change trend:** 0.13 (95% CI: 0.06 to 0.20) P<0.01	**Change trend:** -0.09 (95% CI:-0.13 to -0.05) P<0.01	
**0-15**	-9.19 (95% CI: -10.81 to -7.57) P<0.01	-0.21 (95% CI: -0.53 to 0.12) P=0.22	**Change level:** 6.06 (95% CI:-0.49 to 12.62) P=0.07	**Change level:** -0.41 (95% CI: -5.98 to 5.17) P=0.89	**Change level:** -0.54 (95% CI:-5.31 to 4.22) P=0.82	Untransformed 1.34 Prais-Winsten 1.87
			**Change trend:** -0.10 (95% CI:-0.29 to 0.49) P=0.61	**Change trend:** -0.05 (95% CI: -0.27 to 0.16) P=0.63	**Change trend:** 0.06 (95% CI:-0.07 to 0.18) P=0.37	
**16-64**	-1.75 (95% CI:-2.48 to -1.01) P<0.01	0.06 (95% CI: -0.07 to 0.19) P=0.39	**Change level:** 2.12 (95% CI:-0.54 to 4.79) P=0.12	**Change level:** -3.54 (95% CI:-5.76 to -1.33) P<0.01	**Change level:** -0.87 (95% CI: -2.74 to 0.99)P=0.36	Untransformed 1.45 Prais-Winsten 2.10
			**Change trend:** -0.20 (95% CI: -0.35 to -0.05) P<0.01	**Change trend:** 0.18 (95% CI: -0.10 to 0.27) P<0.01	**Change trend:** -0.18 (95% CI:-0.23 to -0.13)P<0.01	
**65+**	1.67 (95% CI: 0.32 to 3.02) P=0.02	0.17(95% CI: -0.04 to 0.39) P=0.11	**Change level:** 2.28 (95% CI: -2.13 to 6.86) P= 0.30	**Change level:** -2.70 (95% CI: -6.39 to 0.99) P=0.15	**Change level:** 0.85 (95% CI:-2.22 to 3.93)P=0.59	Untransformed 1.70 Prais-Winsten 2.00
			**Change trend:** -0.16 (95% CI: -0.40 to 0.09) P=0.21	**Change trend** 0.15 (95% CI: 0.01 to 0.28) P=0.03	**Change trend:** 0.05 (95% CI: -0.03 to 0.12) P=0.23	

For peer review only

Both SIGN guidelines were associated with a reduction in trend in the percentage of TBI admissions resulting in neurosurgery: 1st guideline (-0.06; 95% CI: -0.12 to 0.001) and 2nd guideline (-0.01; 95% CI: -0.03 to -0.003) (Table 2 and supplementary material 6). A 12-month implementation lag did not materially affect the estimates (Supplementary Material 7).

Deaths following admission for TBI:

Neither guideline was associated with a change in level or trend in monthly death rate within 30 days of admission with an ICD 10 code indicating TBI (Table 2 and Supplementary Material 8). The introduction of the 2nd SIGN guideline was associated with a significant reduction in the underlying trend in the monthly percentage of inpatient admissions for TBI that resulted in death within 30 days (-0.03; 95% CI: -0.04 to -0.01) (Table 2 and Supplementary Material 9).

Introduction of a 12-month implementation lag into the models did not materially change the estimates (Supplementary Material 10).

Discussion:

Summary:

To our knowledge, this is the first study to evaluate the impact of the SIGN guidelines and the 4-hour ED target on head injury hospital admissions. We found evidence that the SIGN guidelines reduced inpatient admissions, the effect varying by age group. In the 16-64 age group both guidelines were associated with a reduction in hospital admissions (Fig. 1c and Table 3). This may be due to increased CT imaging in the ED identifying more patients without TBI safe for discharge. In the paediatric population there was an underlying trend of reducing hospital admissions that the guidelines did not appear to affect. In the ≥65 age group neither SIGN guideline iteration acted to offset the secular trend of increasing hospital admissions. Inpatient admissions increased in winter months in the ≥65 age group but were reduced in other age groups. Falls from standing are known to be the most common cause of brain injuries in those ≥65, whilst assault and road traffic accidents are more common causes of injuries in younger patients.[22] Weather conditions during winter

For peer review only - http://bmjopen.bmj.com/site/about/guidelines.xhtml

may increase the likelihood of falls from standing whilst reducing the prevalence of assault or road traffic accidents.[23]

The introduction of both SIGN guideline iterations were associated with an increased in hospital admissions for patients with TBI and a reduction in the proportion of inpatient admissions that resulted in neurosurgery or death (Table 2). The guidelines may have acted, as previously hypothesised, to increase CT diagnosis and admissions of patients with brain injuries of lower severity who do not require intervention.[13] The 4-hour target was associated with an increase in hospital admissions for adults (Fig. 1c, 1d and Table 3). This effect was reversed by the 2nd SIGN guideline in the 16-64 but not the ≥65 age group.

The 4-hour target's effect on adult head injury hospital admissions appears related to the time from admission which CT imaging is recommended . The 1st guideline contained no recommendation for when CT imaging should occur. The introduction of the 4- hour ED target increased head injury hospital admissions in both the 16-64 and ≥65 age groupings, presumably as patients were admitted to await imaging to comply with the target. The 2nd SIGN guideline increased the indications for immediate CT imaging in the 16-64 age group and was associated with a downward trend in admissions. A reduction in hospital admissions was not observed in the ≥65 age group following the 2nd SIGN guideline. The 2nd SIGN guideline includes a series of specific additional indications for imaging in the ≥65 age group that are recommended to occur within 8 hours of ED attendance. As 8 hours is longer than the 4-hour target patients with these indications for CT imaging would be admitted to hospital. This may account for why the 2nd SIGN guideline was not associated with reduced hospital admissions for those ≥65.

Strengths:

We have used a time series of 216 data points and followed established techniques to control for seasonal factors and auto-correlation.[15] We have adjusted for population factors using mid-year population estimates that incorporate the changing demography of Scotland's population. The models constructed were robust to sensitivity analysis for time lags.

There is controversy regarding which ICD10 codes correspond to clinical definitions of head injury and TBI with inconsistent sets of ICD10 codes used to encompass both.[24] We selected

For peer review only - http://bmjopen.bmj.com/site/about/guidelines.xhtml

subsets of codes for head injury that are likely to be sensitive to changes in admission practice related to increased diagnostic precision of TBI from increased CT imaging and inpatient admissions of patients awaiting an exclusion of TBI due to the 4-hour target. Our ICD10 code selection for TBI was intended to encompass radiologically detected injuries as this outcome would be sensitive to increased diagnoses of TBI by CT imaging.

Weaknesses:

Ideally the effects of guideline implementation and policy interventions would be assessed using randomised control trials. However, interrupted time series analysis (a rigorous quasi-experimental study design) is becoming increasingly popular particular for the evaluation of health care practice, programmes and policy because it allows causal inferences when interventions are introduced at specific time points. Discontinuities in outcomes, observed at or shortly after the time of intervention, constitute persuasive evidence of an effect with high internal and external validity.[14 15 25 26] The method has limitations notably the potential for observed discontinuities to result from co-interventions instead of the health policies under investigation. We cannot find other policies or sudden changes to the population of Scotland that could account for the observed changes in admissions for head injury in Scotland at the time of the either the introduction of the SIGN guidelines or the 4-hour ED target.

This study used routinely collected Scottish Information Services Division (ISD) data, and administrative data should be approached with some caution.[27] There may be inaccuracies in diagnostic codes due to coding errors. However, there were no changes to the cohort of admitted patients that data was collected on during the study period and ISD data has been found to be both sufficiently reliably and comprehensively collected to support its use in research.[28 29] Furthermore, random poor coding, without changes in coding practice, are unlikely to result in sharp discontinuities at the specific time points of the policies considered here. ICD coding changed in 1996, so we only used ISD data from 1998 to give time for adjustment. This limited the number of data points before the 1st SIGN guideline, so we may have lacked the power to detect some changes associated with the 1st guideline as statistically significant (Table 3 and Fig. 1).

For peer review only - http://bmjopen.bmj.com/site/about/guidelines.xhtml

We could not stratify all the analysis by age group as the small number of some outcomes prevented release of aggregated data. A sensitivity analysis using ICD10 injury subtypes was also not possible due to the aggregated nature of the available data. A more sensitive outcome measure for changes in admission practice due to either the SIGN guidelines or 4-hour target may be the proportion of attendances to the ED following head injury that result in inpatient hospital admission. TBI has become more common in the elderly and if analysis of the proportion of ED attendances that resulted in inpatient admission was stratified by age this would account for age group differences in incidence of injuries .[30]

There is evidence that deaths from severe TBI fell following the introduction of the NICE guidelines.[31] We may have missed this effect due to the undifferentiated cohort of TBI patients used to assess deaths and the use of all-cause mortality. We also could not adjust mortality by age or injury severity.

Estimates of the impact of guidelines will depend on the extent to which the guidelines have been implemented. There are no national audit data on this, though one local audit conducted in 2001 indicated less than half of patients the SIGN guidelines deemed were safe for discharge from the ED were actually discharged.[32] Our study may therefore underestimate the potential impact of full implementation.

Comparison to previous literature:

A study that assessed the NICE head injury guideline's impact using English data (although not using an interrupted time series) found that increased CT imaging led to an increase in hospital admissions, contrary to expectations.[13] Increased admissions following the introduction of the NICE guidelines could be due to increased diagnosis of TBI due to more CT imaging or the effect of the 4-hour ED target.[13] We found evidence that both factors increased hospital admissions in Scotland.

Analysis of English HES data from 2000-2011 found yearly paediatric hospital admissions for head injury increased from 34 to 37 inpatient admissions per 10 000 children.[33] We found Scottish paediatric admissions fell from 56 per 10 000 children in 1999 to 32 per 10 000 children in 2015. There is evidence that clinicians are less likely to implement head injury guidelines in children.[33-35] A lack of implementation may explain why the SIGN guidelines were not associated with a reduction in paediatric hospital admissions.

For peer review only - http://bmjopen.bmj.com/site/about/guidelines.xhtml

Implications:

The overall effect of the SIGN guidelines was to reduce inpatient hospital admissions for head injured patients and this supports previous research indicating early CT imaging may represent a cost-effective management strategy.[9] However, the guidelines were associated with increased inpatient admissions for patients with TBI, possibly resulting from increased imaging identifying more TBI of lower clinical severity. Research better characterising the risk associated with TBI identified by CT imaging could help identify a sub-set of low-risk patients who could be safely discharged from the ED. This could help mitigate the increase in inpatient hospital admissions in this group associated with the SIGN and other similar guidelines.

We found evidence that the 4-hour ED target acted to reverse reductions in hospital admissions in the 16-64 age group associated with the SIGN guidelines. As has been previously argued, performance targets need to be carefully considered before implementation to ensure that they do not have unintended consequences, in this case undermining the benefits of evidence-based clinical guidelines.[36] A more granular approach to the 4-hour ED target that reflects condition specific clinical circumstances, such as the time frame of CT imaging in head injury, could help to prevent such costly unintended consequences.

Given the limitations in the mortality analysis undertaken it is hard to draw conclusions about how effective the SIGN guidelines were at reducing deaths from TBI. Future analysis should attempt to adjust for age and severity of injury and this will require patient level data.

Conclusion:

Increased early CT imaging of head injured patients may reduce hospital admissions. However, this effect may be offset by an increase in the diagnosis of TBI of lower severity and the 4-hour ED target. Future research should aim to better risk stratify patients with TBI identified by CT imaging to help reduce hospital admissions related to increased CT imaging. Care should also be taken when introducing arbitrary performance targets, such as the 4-hour target, to ensure they do not undermine the beneficial effect of clinical guidelines.

For peer review only - http://bmjopen.bmj.com/site/about/guidelines.xhtml
For peer review only

Acknowledgements:

Sir Graham Teasdale commented on the manuscript and provided invaluable historical context and knowledge regarding the use of administrative data sets in traumatic brain injury research. The Hull and East Yorkshire NHS Trust Trans-Humber Consumer Research Panel and Hull branch of the Headway charity helped develop the research questions addressed in this study.

Competing interests:

The authors declare that they have no competing interests.

Funding:

Carl Marincowitz is funded by a National Institute for Health Research Doctoral Fellowship (DRF-2016-09-086). This study presents independent research funded by the National Institute for Health Research (NIHR). The views expressed are those of the author(s) and not necessarily those of the NHS, the NIHR or the Department of Health.

Authors' contributions:

This idea for the study was conceived by Carl Marincowitz with help from Trevor Sheldon, Fiona Lecky and Victoria Allgar. The analysis was completed by Carl Marincowitz and Eleanor Morris with specialist advice regarding interrupted time series analysis from Trevor Sheldon and Victoria Allgar. Fiona Lecky provided specialist advice regarding the clinical context and interpretation of the results. All authors read and approved the final manuscript.

Data Sharing:

The data used for this analysis was purchased for a fee from Information Service Division Scotland. The corresponding author is happy to share the exact details of the data extract and how to acquire it from ISD Scotland..

Figures:

Figure 1: The Impact of the 4-hour target and SIGN guidelines on head injury admissions

Figure 2: The impact of the SIGN guidelines and 4-Hour Target on admissions for Traumatic Brain Injury

For peer review only - http://bmjopen.bmj.com/site/about/guidelines.xhtml

References:

1. NICE. National Clinical Guidance Centre. (2014). CG 176 Head Injury Triage, assessment, investigation and early management of head injury in children, young people and adults. National Institute for Health and Care Excellence. In: NICE, ed. UK: DOH, 2014.
2. Early management of a patient with a head injury: a national clinical guideline. Edinburgh: SIGN 2009.
3. Miller JD. Minor, moderate and severe head injury. *Neurosurg Rev* 1986;9(1-2):135-9.
4. Kim J, Kemp S, Kullas K, et al. Injury patterns in patients who "talk and die". *J Clin Neurosci* 2013;20(12):1697-701. doi: 10.1016/j.jocn.2013.02.021
5. af Geijerstam JL, Oredsson S, Britton M, et al. Medical outcome after immediate computed tomography or admission for observation in patients with mild head injury: randomised controlled trial. *BMJ* 2006;333(7566):465. doi: 10.1136/bmj.38918.669317.4F
6. Stiell IG, Wells GA, Vandemheen K, et al. The Canadian CT Head Rule for patients with minor head injury. *Lancet* 2001;357(9266):1391-6.
7. Network SIGN. Guideline 46: Early Management of Patients with a Head Injury. 2000
8. Shivaji T, Lee A, Dougall N, et al. The epidemiology of hospital treated traumatic brain injury in Scotland. *BMC Neurology* 2014;14(1):2. doi: 10.1186/1471-2377-14-2
9. Pandor A, Goodacre S, Harnan S, et al. Diagnostic management strategies for adults and children with minor head injury: a systematic review and an economic evaluation. *Health Technol Assess* 2011;15(27):1-202. doi: 10.3310/hta15270
10. Dunning J, Daly JP, Malhotra R, et al. The implications of NICE guidelines on the management of children presenting with head injury. *Arch Dis Child* 2004;89(8):763-7. doi: 10.1136/adc.2003.042523
11. Hassan Z, Smith M, Littlewood S, et al. Head injuries: a study evaluating the impact of the NICE head injury guidelines. *Emerg Med J* 2005;22(12):845-9. doi: 10.1136/emj.2004.021717
12. Comptroller and Auditor General. Improving Emergency Care in England. *National Audit Office* 2004;HC 1075 Session 2003-2004
13. Goodacre S. Hospital admissions with head injury following publication of NICE guidance. *Emerg Med J* 2008;25(9):556-7. doi: 10.1136/emj.2007.055723
14. Wagner AK, Soumerai SB, Zhang F, et al. Segmented regression analysis of interrupted time series studies in medication use research. *J Clin Pharm Ther* 2002;27(4):299-309.
15. Lopez Bernal J, Cummins S, Gasparrini A. Interrupted time series regression for the evaluation of public health interventions: a tutorial. *Int J Epidemiol* 2016 doi: 10.1093/ije/dyw098 [published Online First: 2016/06/11]
16. http://apps.who.int/classifications/icd10/browse/2010/en [
17. National Clinical Coding Standards - OPCS-4 (2017) Reference Book.
18. http://www.isdscotland.org/Products-and-Services/Terminology-Services/Coding-and-Terminology-Systems/ [
19. Penfold RB, Zhang F. Use of interrupted time series analysis in evaluating health care quality improvements. *Acad Pediatr* 2013;13(6 Suppl):S38-44. doi: 10.1016/j.acap.2013.08.002 [published Online First: 2013/12/07]
20. Linden A. Conducting interrupted time-series analysis for single- and multiple-group comparisons. *The Stata Journal* 2015;15(2):480-500.
21. Hamilton I, Lloyd C, Hewitt C, et al. Effect of reclassification of cannabis on hospital admissions for cannabis psychosis: a time series analysis. *Int J Drug Policy* 2014;25(1):151-6. doi: 10.1016/j.drugpo.2013.05.016 [published Online First: 2013/07/23]

For peer review only - http://bmjopen.bmj.com/site/about/guidelines.xhtml

22. Lawrence T, Helmy A, Bouamra O, et al. Traumatic brain injury in England and Wales: prospective audit of epidemiology, complications and standardised mortality. *BMJ Open* 2016;6(11):e012197. doi: 10.1136/bmjopen-2016-012197
23. Rock DJ, Judd K, Hallmayer JF. The seasonal relationship between assault and homicide in England and Wales. *Injury* 2008;39(9):1047-53. doi: https://doi.org/10.1016/j.injury.2008.03.025
24. Chen AY, Colantonio A. Defining neurotrauma in administrative data using the International Classification of Diseases Tenth Revision. *Emerg Themes Epidemiol* 2011;8(1):4. doi: 10.1186/1742-7622-8-4 [published Online First: 2011/05/17]
25. Kontopantelis E, Doran T, Springate DA, et al. Regression based quasi-experimental approach when randomisation is not an option: interrupted time series analysis. *BMJ* 2015;350:h2750. doi: 10.1136/bmj.h2750
26. Barnighausen T, Tugwell P, Rottingen JA, et al. Quasi-experimental study designs series-paper 4: uses and value. *J Clin Epidemiol* 2017;89:21-29. doi: 10.1016/j.jclinepi.2017.03.012 [published Online First: 2017/04/04]
27. Hand DJ. Statistical challenges of administrative and transaction data. *J R Statist Soc A* 2018 (pre-publication presented at The Royal Statistical Society);181(Part 3):1-24.
28. Burns EM, Rigby E, Mamidanna R, et al. Systematic review of discharge coding accuracy. *Journal of Public Health* 2012;34(1):138-48. doi: 10.1093/pubmed/fdr054
29. Harley K, Jones C. Quality of Scottish Morbidity Record (SMR) data. *Health Bull (Edinb)* 1996;54(5):410-7. [published Online First: 1996/09/01]
30. Woodford TCAERGDSKFLISSTAWM. MAJOR TRAUMA IN OLDER PEOPLE. *The Traruma and Audit Research Network Report* 2017
31. Fuller G, Bouamra O, Woodford M, et al. Temporal trends in head injury outcomes from 2003 to 2009 in England and Wales. *Br J Neurosurg* 2011;25(3):414-21. doi: 10.3109/02688697.2011.570882
32. Kerr J, Smith R, Gray S, et al. An audit of clinical practice in the management of head injured patients following the introduction of the Scottish Intercollegiate Guidelines Network (SIGN) recommendations. *Emerg Med J* 2005;22(12):850-4. doi: 10.1136/emj.2004.022673 [published Online First: 2005/11/22]
33. Marlow R, Mytton J, Maconochie IK, et al. Trends in admission and death rates due to paediatric head injury in England, 2000-2011. *Arch Dis Child* 2015;100(12):1136-40. doi: 10.1136/archdischild-2015-308615
34. Plumb J. 'My 15-month-old child fell over and bumped his head doctor. He's fine now but he's been sick 3 times since it happened'. "Oh dear, that's not so NICE". *Br J Neurosurg* 2009;23(6):574; author reply 75. doi: 10.3109/02688690903419115 [published Online First: 2009/11/20]
35. Mooney JS, Yates A, Sellar L, et al. Emergency head injury imaging: implementing NICE 2007 in a tertiary neurosciences centre and a busy district general hospital. *Emerg Med J* 2011;28(9):778-82. doi: 10.1136/emj.2009.083360
36. Sheldon T. Promoting health care quality: what role performance indicators? *Qual Health Care* 1998;7 Suppl:S45-50. [published Online First: 1999/05/29]

For peer review only

For peer review only - http://bmjopen.bmj.com/site/about/guidelines.xhtml

Figure 1:

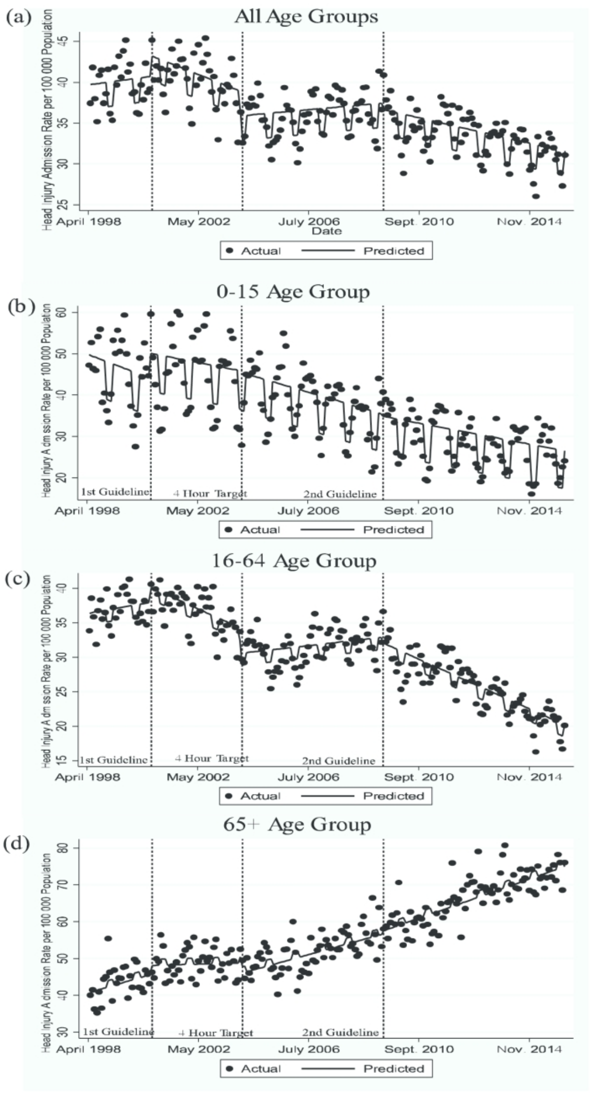

Figure 1: The Impact of the 4-hour target and SIGN guidelines on head injury admissions

210x246mm (300 x 300 DPI)

Figure 2:

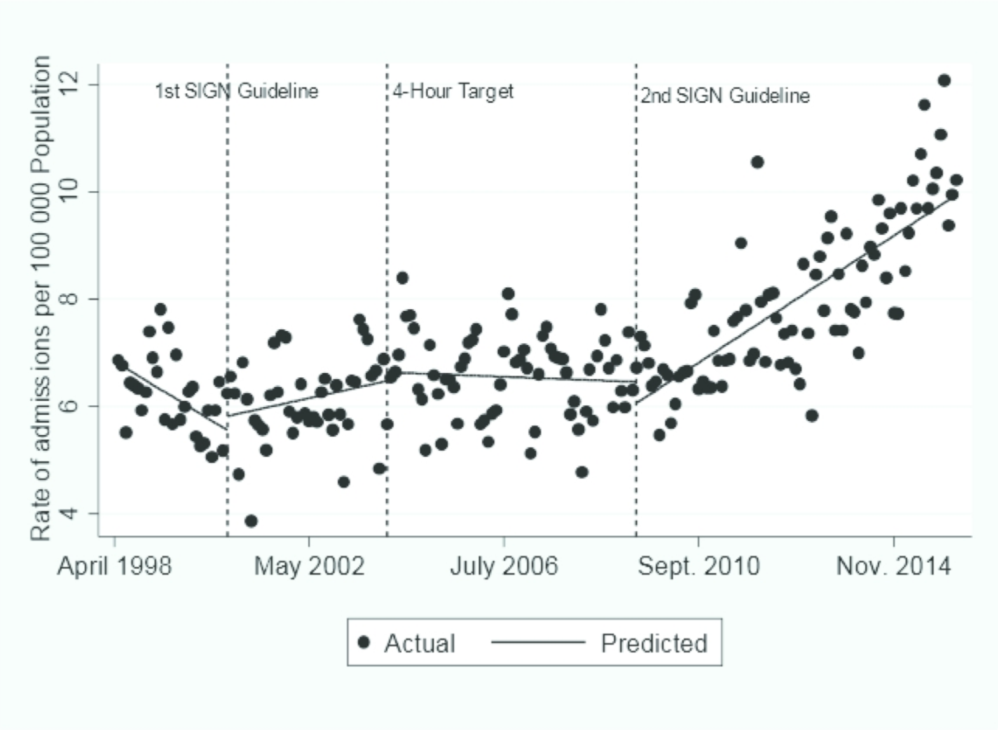

Figure 2: The impact of the SIGN guidelines and 4-Hour Target on admissions for Traumatic Brain Injury

206x159mm (300 x 300 DPI)

Supplementary Material 1: Summary of Health policies assessed

Policy	Time of Introduction	Key Features
1st SIGN Head Injury Guideline	August 2000	Indication for CT imaging (referenced directly from 2000 SIGN guideline):<br>(1) GCS<13<br>(2) A deteriorating level of consciousness or progressive focal neurological signs<br>(3) Confusion or drowsiness (CGS 13 or 14/15) followed by failure to improve within at most four hours of clinical observation<br>(4) Radiological/clinical evidence of a fracture, whatever the level of consciousness<br>(5) New focal neurological signs which are not getting worse<br>(6) Full consciousness (GCS 15/15) with no fracture but other features, including:<br>– severe and persistent headache<br>– nausea and vomiting<br>– irritability or altered behaviour<br>– a seizure.<br><br>Skull films should be carried out if any of the following apply and if CT is not being performed:<br>If the patient is alert and orientated and obeying commands (GCS 15/15) but:<br>– the mechanism of injury has not been trivial; or<br>– consciousness has been lost; or<br>– the patient has loss of memory or has vomited; or<br>– the scalp has a full thickness laceration or a boggy haematoma; or<br>– the history is inadequate.
4-Hour ED performance target	2004	98% of patients attending the ED to be assessed, treated and either discharged or admitted to hospital within 4 hours of arrival. Financial incentives associated with meeting the target.
2nd Sign Head Injury Guideline	May 2009	Referenced directly from 2nd SIGN Guidelines<br><br>Indications Immediate CT scanning (adult):<br>- GCS<13/15<br>- GCS 13/15 or 14/15 followed by failure to improve within one hour of clinical observation<br>- base of skull or depressed skull fracture and/or suspected penetrating injuries<br>- a deteriorating level of consciousness or new focal neurological signs<br>- GCS 15/15 with severe and persistent headache or two episodes of vomiting<br>- coagulopathy (eg warfarin use) and loss of consciousness or amnesia<br>Indications CT scanning within eight (adult):<br>- age>65 (with loss of consciousness or amnesia)

For peer review only

		- clinical evidence of a skull fracture but no clinical features indicative of an immediate CT scan<br>- any seizure activity<br>- significant retrograde amnesia (>30 minutes)<br>- dangerous mechanism of injury<br><br>Indications Immediate CT scanning (<16 years):<br>- GCS≤13 on assessment in ED<br>- witnessed loss of consciousness >5 minutes<br>- suspicion of open or depressed skull injury or tense fontanelle<br>- focal neurological deficit<br>- any sign of basal skull fracture.<br>Indications CT scanning within 8 hours (<16 years):<br>- presence of any bruise/swelling/laceration >5 cm on the head<br>- post-traumatic seizure<br>- amnesia (anterograde or retrograde) lasting >5 minutes<br>- clinical suspicion of non-accidental head injury<br>- a significant fall<br>- age under one year: GCS<15 in emergency department<br>- three or more discrete episodes of vomiting<br>- abnormal drowsiness.

For peer review only

Supplementary Material 2: Changes to Scotland's population structure

i) Mid-year Estimate Scottish Population 0-15

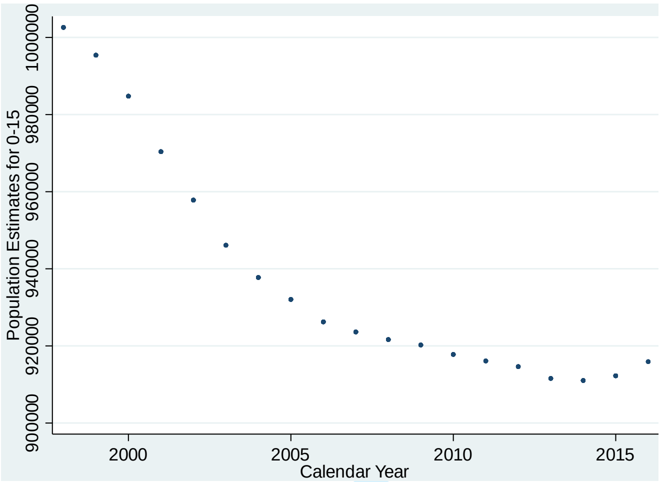

ii) Mid-year Estimate Scottish population 16-64

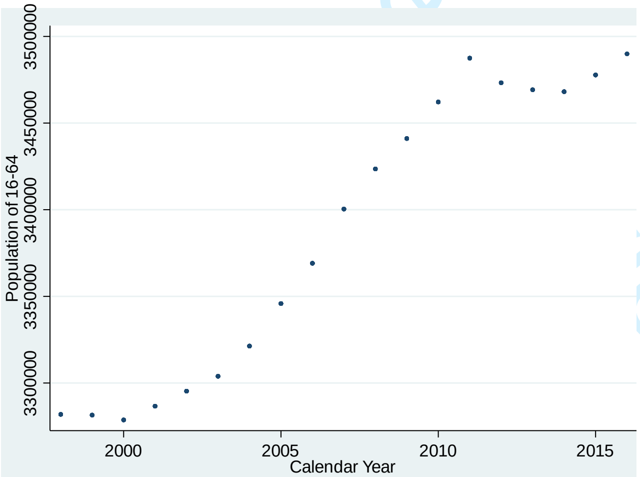

iii) Mid-year Estimate Scottish population 65+

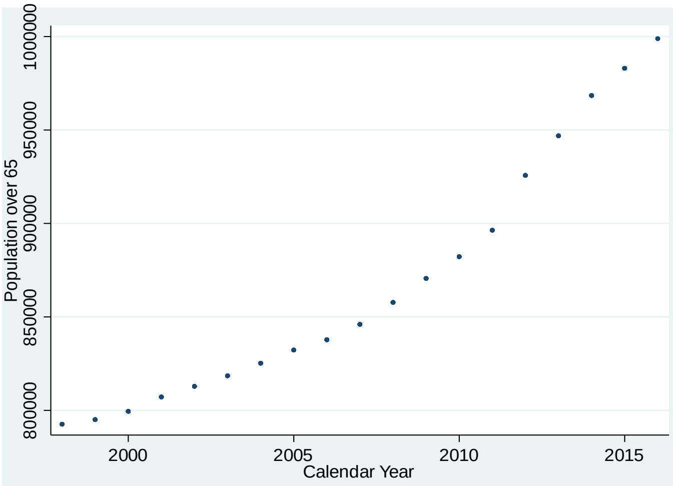

For peer review only

Supplementary Material 3: Increase in admissions related to the introduction of the 4-hour target in the 16-64 age group

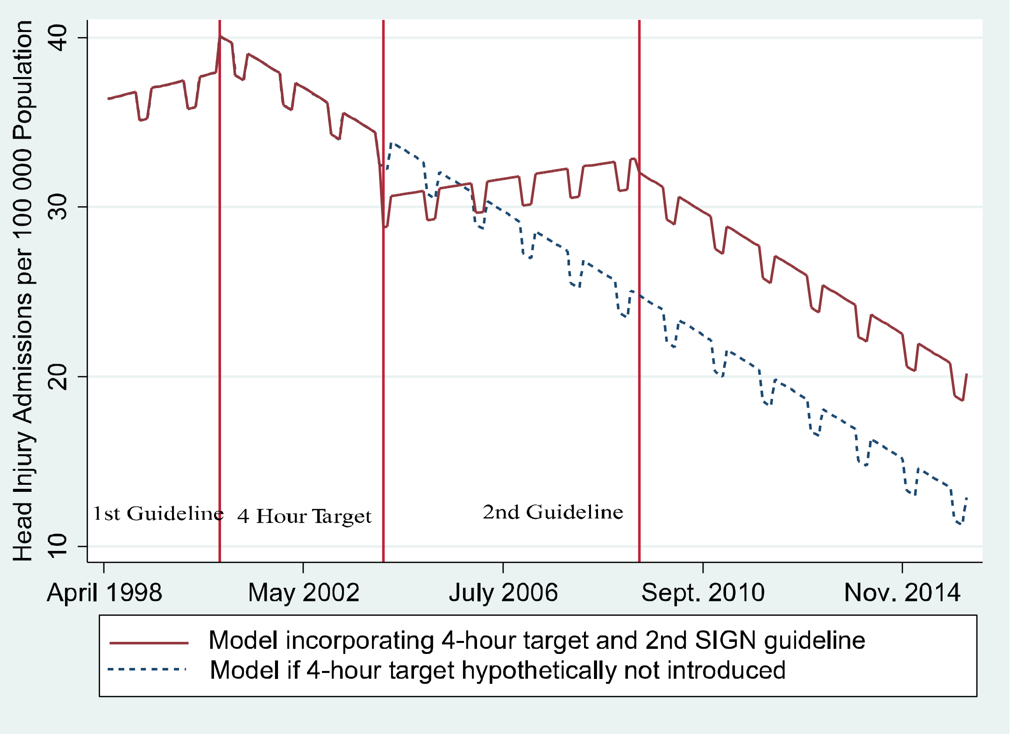

For peer review only

Supplementary Material 4: Estimate of Impact of the SIGN guidelines and introduction of 4-Hour Target admissions for head injury with 12-month time lag

Age Band	Winter	Initial Trend	1st SIGN Guideline	4-hour Target Introduced	2nd SIGN Guideline	Durbin-Watson Statistic
**All ages**	-3.00 (95% CI: -3.75 to -2.26) P<0.01	0.04 (95% CI: -0.07 to 0.14) P=0.48	**Change level:** 2.66 (95% CI:0.27 to 5.04) P=0.03	**Change level:** -2.73 (95% CI:-4.85 to -0.61) P<0.01	**Change level:** -2.07 (95% CI:-3.74 to -0.40) P=0.02	Untransformed 1.77<br>Prais-Winsten 2.00
			**Change trend:** -0.23 (95% CI:-0.38 to -0.08) P<0.01	**Change trend:** 0.25 (95% CI: 0.14 to 0.36) P<0.01	**Change trend:** -0.13 (95% CI: -0.18 to -0.08) P<0.01	
**0-15**	-9.31 (95% CI: -11.05 to -7.59) P<0.01	-0.18 (95% CI:-4.89 to 0.13) P=0.26	**Change level:** 8.53 (95% CI:1.93 to 15.13) P=0.01	**Change level:** 1.61 (95% CI:-4.39 to 7.61) P=0.60	**Change level:** -1.71 (95% CI:-6.61 to 3.20) P=0.49	Untransformed 1.37<br>Prais-Winsten 1.85
			**Change trend:** -0.17 (95% CI:-0.61 to 0.28) P=0.47	**Change trend:** 0.15 (95% CI: -0.17 to 0.48) P=0.36	**Change trend:** 0.10 (95% CI:-0.50 to 0.26) P=0.18	
**16-64**	-1.80 (95% CI:-2.56 to -1.04) P<0.01	0.06 (95% CI:-0.06 to 0.17) P=0.34	**Change level:** 1.29 (95% CI:-1.28 to 3.85) P=0.32	**Change level:** -4.19 (95% CI:-6.48 to -1.89) P<0.01	**Change level:** -3.06 (95% CI:-4.88 to -1.24) P<0.01	Untransformed 1.57<br>Prais-Winsten 2.05
			**Change trend:** -0.25 (95% CI:-0.41 to -0.09) P<0.01	**Change trend:** 0.29 (95% CI: -0.17 to 0.40) P<0.01	**Change trend:** -0.25 (95% CI:-0.30 to -0.19) P<0.01	
**65+**	1.69 (95% CI: 0.22 to 3.17) P=0.03	0.17 (95% CI:-0.04 to 0.38) P= 0.10	**Change level:** 3.64 (95% CI:-1.08 to 8.36) P= 0.13	**Change level:** -1.64 (95% CI:-5.83 to 2.55) P=0.44	**Change level:** 2.30 (95% CI:-1.00 to 5.60) P=0.17	Untransformed 1.78<br>Prais-Winsten 2.00
			**Change trend:** -0.22 (95% CI:-0.51 to 0.07) P=0.14	**Change trend** 0.26 (95% CI: 0.04 to 0.48) P=0.02	**Change trend:** 0.003 (95% CI:-0.10 to 0.10) P=0.95	

For peer review only

Supplementary Material 5: Increase in admissions related to the introduction of the 2nd SIGN guideline

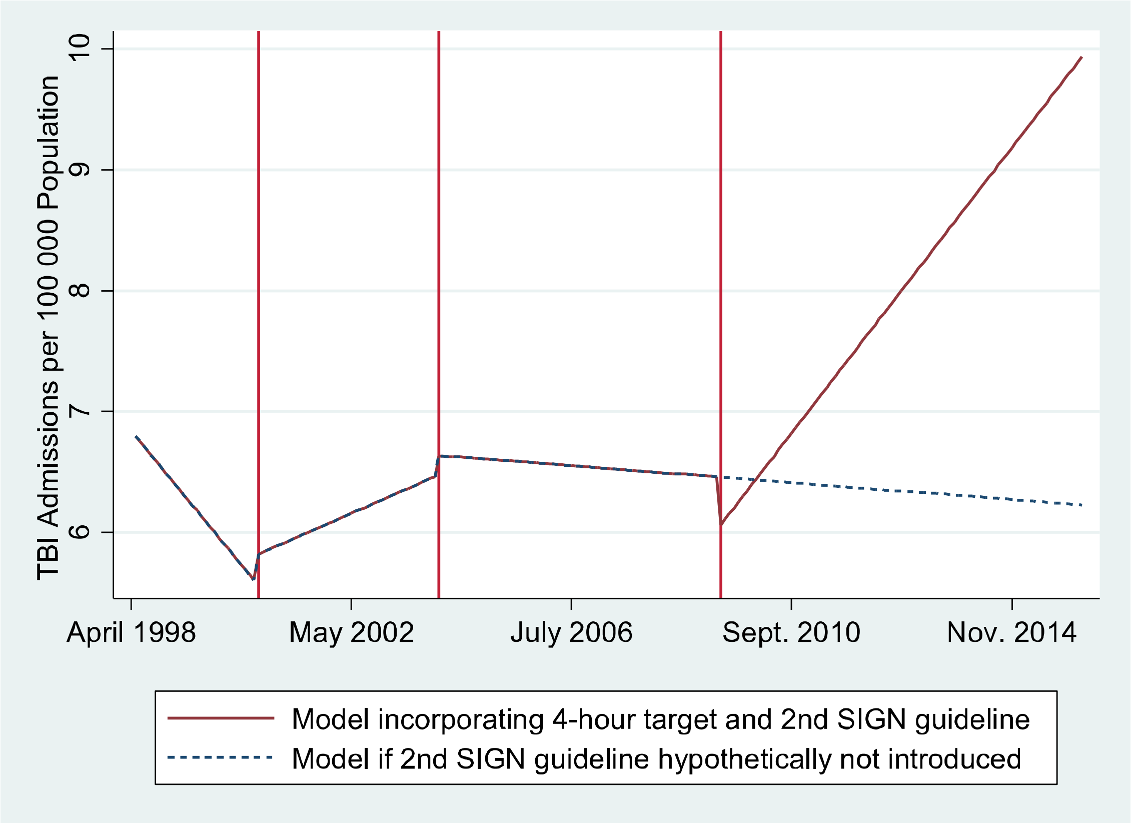

Supplementary Material 6: The impact of the SIGN guidelines on the percentage of admissions for TBI that resulted in neurosurgery

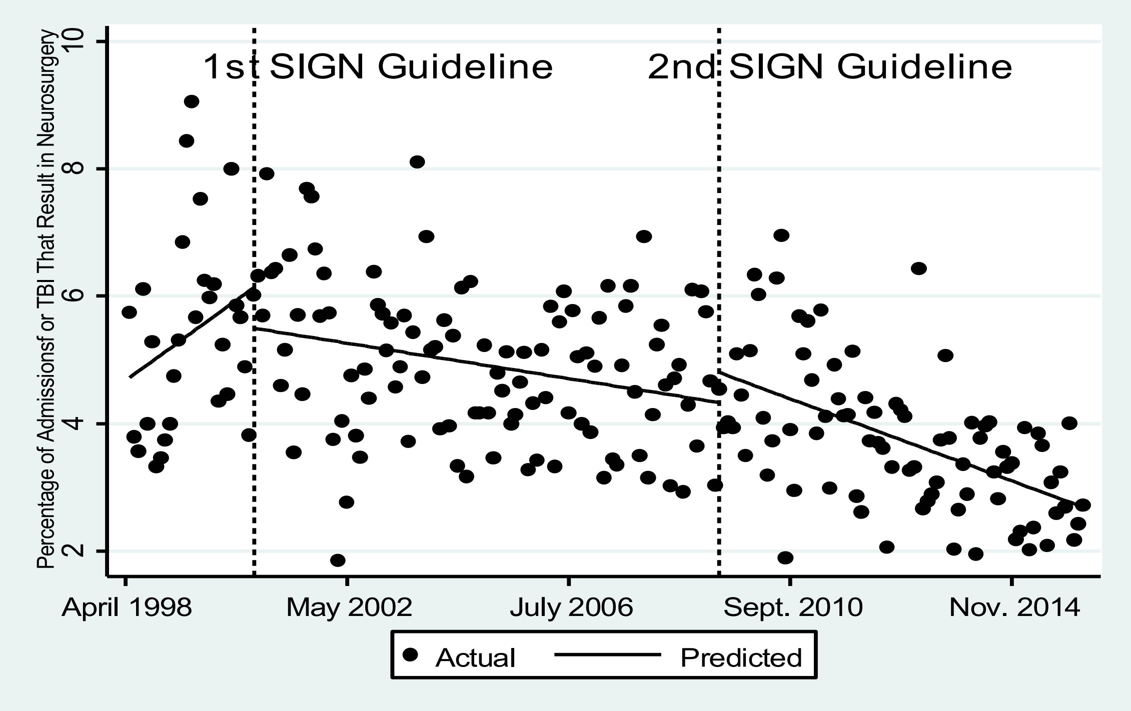

Supplementary Material 7: Estimate of impact of the SIGN guidelines and 4-Hour Target on admissions for Traumatic Brain Injury with 12-month time lag

Outcome	Initial Trend	1st SIGN Guideline	4-hour Target Introduced	2nd SIGN Guideline	Durbin-Watson Statistic
**Admissions for TBI/ 100 000**	-0.05 (95% CI: -0.10 to 0.003) P=0.07	**Change level:** 0.75 (95% CI:-0.34 to 1.84) P=0.18	**Change level:** 0.35 (95% CI:-0.63 to 1.33) P=0.48	**Change level:** -0.16 (95% CI: -0.92 to 0.60) P=0.69	Untransformed 1.44 Prais-Winsten 2.03
		**Change trend:** 0.05 (95% CI:-0.02 to 0.12) P=0.16	**Change trend:** -0.01 (95% CI:-0.06 to 0.04) P=0.66	**Change trend:** 0.05 (95% CI:3 to 0.08) P<0.01	
**Percentage TBI admissions neurosurgical**	0.05 (95% CI: -0.01 to 0.11) P=0.10	**Change level:** -0.96 (95% CI:-2.19 to 0.28) P=0.13		**Change level:** 0.17 (95% CI:-0.59 to 0.93) P=0.66	Untransformed 1.85
		**Change trend:** -0.06 (95% CI:-0.12 to 0.003) P=0.06		**Change trend:** -0.02 (95% CI:-0.04 to-0.004) P=0.01	

For peer review only

Supplementary Material 8: The impact of the SIGN guidelines on deaths per 100 000 population within 30 days of admission with traumatic brain injury

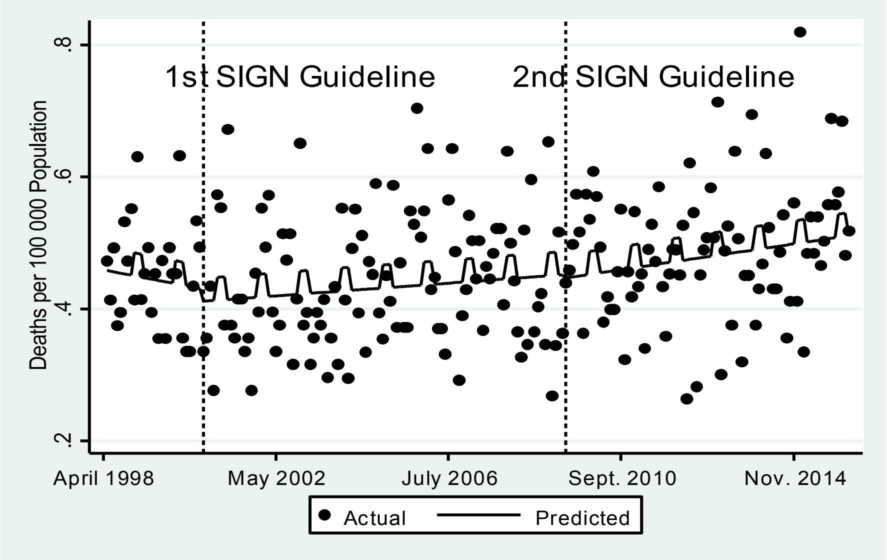

Supplementary Material 9: The impact of the SIGN guidelines on the percentage of patients admitted with TBI who died within 30 days of admission

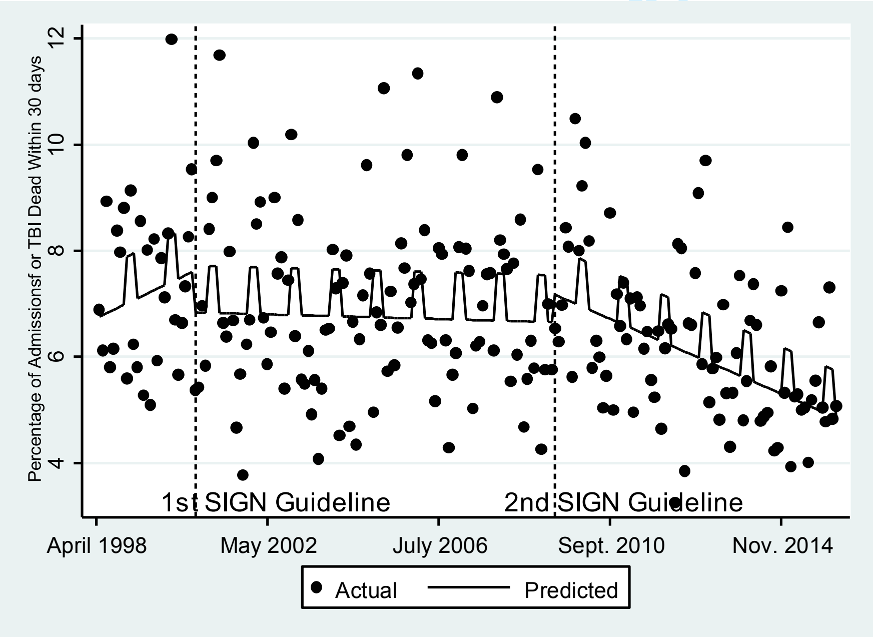

For peer review only

Supplementary Material 10: Estimate of impact of the SIGN guidelines on deaths following admission for Traumatic Brain Injury with 12 month time lag

**Outcome**	**Winter**	**Initial Trend**	**1st SIGN Guideline**	**2nd SIGN Guideline**	**Durbin-Watson Statistic**
Deaths/100 000	0.03 (95% CI:-0.01 to 0.06) P=0.15	-0.001 (95% CI:-0.004 to 0.003) P=0.59	**Change level:** -0.02 (95% CI:- 0.09 to 0.06) P=0.66	**Change level:** -0.03 (95% CI:-0.08 to 0.03) P=0.32	Untransformed 2.29
			**Change trend:** 0.001 (95% CI:-0.002 to 0.005) P=0.44	**Change trend:** 0.001 (95% CI:-0.0002 to 0.002) P=0.10	
Percentage TBI admissions death	0.74 (95% CI: 0.18 to 1.30) P=0.10	0.03 (95% CI:-0.03 to 0.10) P=0.34	**Change level:** -1.06 (95% CI: -2.40 to 0.29) P=0.12	**Change level:** -0.37 (95% CI:-1.20 to 0.47) P=0.39	Untransformed 2.17
			**Change trend:** -0.03 (95% CI:-0.10 to 0.04) P=0.38	**Change trend:** -0.02 (95% CI:-0.04 to -0.004) P=0.02	

For peer review only

STROBE Statement—checklist of items that should be included in reports of observational studies

	Item No	Recommendation
**Title and abstract**	1	(a) Indicate the study's design with a commonly used term in the title or the abstract<br>**Page 1**
		(*b*) Provide in the abstract an informative and balanced summary of what was done and what was found<br>**Page 2**
**Introduction**		
Background/rationale	2	Explain the scientific background and rationale for the investigation being reported<br>**Page 4**
Objectives	3	State specific objectives, including any prespecified hypotheses<br>**Page 4**
**Methods**		
Study design	4	Present key elements of study design early in the paper<br>**Pages 5,6**
Setting	5	Describe the setting, locations, and relevant dates, including periods of recruitment, exposure, follow-up, and data collection<br>**Pages 5,6**
Participants	6	(*a*) *Cohort study*—Give the eligibility criteria, and the sources and methods of selection of participants. Describe methods of follow-up<br>**Pages 5,6**
		(*b*) *Cohort study*—For matched studies, give matching criteria and number of exposed and unexposed<br>**Pages 5,6**
Variables	7	Clearly define all outcomes, exposures, predictors, potential confounders, and effect modifiers. Give diagnostic criteria, if applicable<br>**Pages 6,9**
Data sources/ measurement	8*	For each variable of interest, give sources of data and details of methods of assessment (measurement). Describe comparability of assessment methods if there is more than one group<br>**Pages 5,6,9**
Bias	9	Describe any efforts to address potential sources of bias<br>**Pages 9,10**
Study size	10	Explain how the study size was arrived at<br>**N/A**
Quantitative variables	11	Explain how quantitative variables were handled in the analyses. If applicable, describe which groupings were chosen and why<br>**Pages 6,9**
Statistical methods	12	(*a*) Describe all statistical methods, including those used to control for confounding<br>**Pages 9,10**
		(*b*) Describe any methods used to examine subgroups and interactions<br>**Pages 9,10**
		(*c*) Explain how missing data were addressed
		(*d*) *Cohort study*—If applicable, explain how loss to follow-up was addressed<br>*Case-control study*—If applicable, explain how matching of cases and controls was

For peer review only

		addressed<br>*Cross-sectional study*—If applicable, describe analytical methods taking account of sampling strategy
		(*e*) Describe any sensitivity analyses<br>**Page 10**
**Results**		
Participants	13*	(a) Report numbers of individuals at each stage of study—eg numbers potentially eligible, examined for eligibility, confirmed eligible, included in the study, completing follow-up, and analysed<br>**Page 7**
		(b) Give reasons for non-participation at each stage
		(c) Consider use of a flow diagram
Descriptive data	14*	(a) Give characteristics of study participants (eg demographic, clinical, social) and information on exposures and potential confounders<br>**Page 7**
		(b) Indicate number of participants with missing data for each variable of interest
		(c) *Cohort study*—Summarise follow-up time (eg, average and total amount)
Outcome data	15*	*Cohort study*—Report numbers of outcome events or summary measures over time
		Case-control study—Report numbers in each exposure category, or summary measures of exposure
		Cross-sectional study—Report numbers of outcome events or summary measures
Main results	16	(*a*) Give unadjusted estimates and, if applicable, confounder-adjusted estimates and their precision (eg, 95% confidence interval). Make clear which confounders were adjusted for and why they were included<br>**Pages 7, 10, 11, 12, 13**
		(*b*) Report category boundaries when continuous variables were categorized
		(*c*) If relevant, consider translating estimates of relative risk into absolute risk for a meaningful time period
Other analyses	17	Report other analyses done—eg analyses of subgroups and interactions, and sensitivity analyses
**Discussion**		
Key results	18	Summarise key results with reference to study objectives<br>**Pages 13, 14**
Limitations	19	Discuss limitations of the study, taking into account sources of potential bias or imprecision. Discuss both direction and magnitude of any potential bias<br>**Pages 14, 15, 16**
Interpretation	20	Give a cautious overall interpretation of results considering objectives, limitations, multiplicity of analyses, results from similar studies, and other relevant evidence<br>**Pages 16, 17**
Generalisability	21	Discuss the generalisability (external validity) of the study results<br>**Pages 16, 17**
**Other information**		
Funding	22	Give the source of funding and the role of the funders for the present study and, if applicable, for the original study on which the present article is based<br><br>**Page 18**

For peer review only

For peer review only - http://bmjopen.bmj.com/site/about/guidelines.xhtml

*Give information separately for cases and controls in case-control studies and, if applicable, for exposed and unexposed groups in cohort and cross-sectional studies.

Note: An Explanation and Elaboration article discusses each checklist item and gives methodological background and published examples of transparent reporting. The STROBE checklist is best used in conjunction with this article (freely available on the Web sites of PLoS Medicine at http://www.plosmedicine.org/, Annals of Internal Medicine at http://www.annals.org/, and Epidemiology at http://www.epidem.com/). Information on the STROBE Initiative is available at www.strobe-statement.org.

For peer review only

BMJ Open

The impact of the SIGN head injury guidelines and NHS 4-hour Emergency Target on hospital admissions for head injury in Scotland: An Interrupted Times Series.

Journal:	*BMJ Open*
Manuscript ID	bmjopen-2018-022279.R2
Article Type:	Research
Date Submitted by the Author:	26-Sep-2018

Complete List of Authors:	Marincowitz, Carl; University of Hull, Hull York Medical School<br>Lecky, Fiona; University of Sheffield, School of Health and Related Research<br>Morris, Eleanor ; Hull York Medical School<br>Allgar, Victoria; Hull York Medical School<br>Sheldon, Trevor; University of York, Health Sciences

**Primary Subject Heading**:	Emergency medicine
Secondary Subject Heading:	Health policy, Radiology and imaging, Diagnostics
Keywords:	Traumatic Brain Injury, Head Injury, SIGN Guidelines, NHS 4-hour Emergency Department Performance Target, Interrupted Time Series

SCHOLARONE™
Manuscripts

The impact of the SIGN head injury guidelines and NHS 4-hour Emergency Target on hospital admissions for head injury in Scotland: An Interrupted Times Series.

Carl Marincowitz[1] MB BChir, MSc, BA

Fiona E. Lecky[2] MB Ch B, FRCS, DA, MSc, PhD, FCEM

Eleanor Morris[3]

Victoria Allgar[4] PhD, Chartered Statistician, Royal Statistical Society, BSc

Trevor A. Sheldon[5] MSc, MSc, DSc, FMedSci

1. **Corresponding Author.** Hull York Medical School, Allam Medical Building, University of Hull, Hull HU6, UK 7RX, Fax: +44 (0) 1482 464705 Tel +44 (0) 870 1245500
Email: carl.marincowitz@hyms.ac.uk

2. School of Health and Related Research, University of Sheffield, Regent Court, 30 Regent Street, Sheffield, S1 4DA, UK, Fax: +44 (0)114 222 0749 Tel: (+44) (0)114 222 4345,
Email: f.e.lecky@sheffield.ac.uk

3. Hull York Medical School, Allam Medical Building, University of Hull, Hull HU6, UK 7RX, Fax: +44 (0) 1482 464705 Tel +44 (0) 870 1245500 Email: hyem7@hyms.ac.uk

4 Hull York Medical School, John Hughlings Jackson Building, University of York, Heslington, York YO10 5DD Tel: 0870 124 5500, Email: Victoria.Allgar@hyms.ac.uk

5. Department of Health Sciences, University of York, Alcuin Research Resource Centre, Heslington, York, YO10 5DD, Tel +44 (0) 1904 321344, Fax: +44 (0) 1904 323 521,
E-mail: trevor.sheldon@york.ac.uk

Word Count: 4081

For peer review only - http://bmjopen.bmj.com/site/about/guidelines.xhtml

For peer review only

Abstract

Objectives:

Head injury is a common reason for Emergency Department (ED) attendance. Around 1% of patients have life-threatening injuries, whilst 80% of patients are discharged. National guidelines (SIGN) were introduced in Scotland with the aim of achieving early identification of those with acute intracranial lesions yet safely reducing hospital admissions.

This study aims to assess the impact of these guidelines and any effect the national 4-hour ED performance targets had on hospital admissions for head injury.

Setting:

All Scottish hospitals between April 1998 and March 2016.

Participants:

Patients admitted to hospital for head injury or traumatic brain injury (TBI) diagnosed by CT imaging identified using administrative Scottish Information Services Division data. There are 275 hospitals in Scotland. In 2015/2016 there were 571, 221 emergency hospital admissions in Scotland.

Interventions:

The SIGN head injury guidelines introduced in 2000 and 2009. The 4-hour ED target introduced in 2004.

Outcomes:

The monthly rate of hospital admissions for head injury and traumatic brain injury.

Study Design:

An interrupted time series analysis.

Results:

The 1st guideline was associated with a reduction in monthly admissions of 0.14 (95% CI:0.09 to 4.83) per 100, 000 population. The 4-hour target was associated with a monthly increase in admissions of 0.13 (95% CI:0.06 to 0.20) per 100, 000 population. The 2nd guideline reduced monthly admissions by 0.09 (95% CI:-0.13 to -0.05) per 100, 000 population. These effects varied between age groups.

The guidelines were associated with increased admissions for patients with injuries identified by CT imaging- Guideline 1: 0.06 (95% CI: 0.004 to 0.12); Guideline 2: 0.05 (95% CI: 0.04 to 0.06) per 100 000 population.

Conclusion:

Increased CT imaging of head injured patients recommended by SIGN guidelines reduced hospital admissions. The 4-hour ED target and the increased identification of TBI by CT imaging acted to undermine this effect.

For peer review only - http://bmjopen.bmj.com/site/about/guidelines.xhtml

Strengths and limitations of this study:

This is the first study to assess the impact of the SIGN head injury guidelines and 4-hour Emergency Department target on hospital admissions for head injury.

We used the robust method of interrupted time series analysis and found the SIGN guidelines acted to reduce hospital admissions, but the 4-hour target increased hospital admissions.

Due to the aggregated nature of the available data we were unable to perform some age group specific and injury sub-group sensitivity analysis.

Keywords:

Traumatic Brain Injury, Head Injury, SIGN Guidelines, NHS 4-hour Emergency Department Performance Target and Interrupted Time Series.

For peer review only

For peer review only - http://bmjopen.bmj.com/site/about/guidelines.xhtml

Background:

There are 1.4 million annual attendances to Emergency Departments (ED) in England and Wales following a head injury (blunt trauma to the head).[1] In Scotland an estimated 6.6% of ED attendances are for head injury.[2] Approximately 95% of patients present with an initial Glasgow Coma Scale (GCS) of 13-15, indicating normal or minimally impaired conscious level and are defined as having a minor head injury.[1 3]

Around 1% of minor head injured patients have life-threatening traumatic brain injuries (TBI) (injury to the brain/ functional impairment due to external force) but this may not be initially clinically apparent.[1] Early identification of severe TBI can facilitate life-saving neurosurgery.[4] The clinical challenge is to differentiate patients with life-threatening TBI who present with a high conscious level from patients who can be discharged safely. This can be achieved through observation for deterioration or cranial CT imaging.[5] The health services challenge is finding a way of differentiating these groups in a way that minimises unnecessary imaging or inpatient hospital admissions of patients without clinically important TBI. Research has focused on developing clinical decision rules that, using clinical assessment, triage patients at risk of life-threatening TBI to CT imaging and allow the discharge of low-risk patients. The most validated, in adults, is the Canadian CT Head Rule (CCHR) and this forms the basis of the Scottish Intercollegiate Guidelines Network (SIGN) and English National Institute for Health and Care Excellence (NICE) head injury guidelines.[1 2 6] The 2nd SIGN guideline contains specific paediatric indications for CT imaging influenced by the CHALICE rule, which was derived in a population of head injured children.[2]

Two iterations of the SIGN guidelines have been introduced (Fig.1).[2 7] The first recommended increased CT imaging of head injured patients but still featured a role for skull x-rays and admission for observation.[7] The 2nd SIGN guideline extended CT imaging further.[2] A study assessing trends in hospital admissions for TBI in Scotland between 1998 and 2009 found changes in rates of admissions at specific time points after 2000, but the analysis was data driven and does not explicitly assess the impact of the SIGN guidelines.[8] Implementation studies of NICE head injury guidelines in England suggested that the cost of increased CT imaging (£100 per scan) would be offset by a reduction in inpatient hospital admissions (£847 per admission) as patients admitted for observation would be dicharged from the ED following normal CT imaging, preventing many admissions.[9-11]

For peer review only - http://bmjopen.bmj.com/site/about/guidelines.xhtml

However, a study using English Hospital Episodes Statistics (HES) found head injury inpatient admissions increased following the introduction of the NICE guidelines, possibly due to the near simultaneous introduction of the 4-hour ED target. Supplementary material 1 presents the details of the 4-target and in 2002 around 23% of all patients in the UK remained in the ED for longer than 4-hours.[12] The target could act to incentivise inpatient admissions for patients at medium risk for TBI, who require imaging within 8 hours, to avoid breaches. Previously they would have been imaged in the ED and the majority discharged. A further cause of increased admissions could be the detection of brain injuries previously not identified, some without clinically significant sequelae, due to increased imaging.[13] In Scotland the 4-hour target was not introduced at the same time as the SIGN guidelines. This provides a unique opportunity to assess the independent effects of these policy interventions.

Aims:

This study aims to assess the impact of the introduction of the SIGN head injury guidelines and the 4-hour target on the rate of hospital admissions in patients with head injury and explores the extent to which any increase in admissions were due to the identification of more TBI.

Methods:

Study Design:

Interrupted time series analysis is an established and robust method for the evaluation of health policies implemented at discrete time points.[14 15]

Dataset:

The Scottish Information Services Division (ISD) contains a repository of information routinely collected at discharge from hospital for all non-obstetric and non-psychiatric inpatient hospital admissions in Scotland (including most short stay admissions to Clinical Decision Units) . Since 1996 reason for admission has been categorised using ICD10 diagnostic coding and since 1989 interventions have been coded using the OPSC4 classification.[16-18] This repository was used, for the period 1998-2016, to generate monthly numbers of: patients admitted with ICD10 coding for head injury, patients admitted with

For peer review only - http://bmjopen.bmj.com/site/about/guidelines.xhtml

For peer review only

ICD coding indicating TBI identified by CT imaging, neurosurgical interventions and deaths within 30 days of admission for patients with CT evidence of TBI. Diagnostic codes were counted if they were either primary or secondary diagnoses.

The data extract used for analysis is available from ISD Scotland for a commercial fee. The data set was fully anonymised and aggregated with small numbers suppressed.

We selected an ICD10 code subset definition of head injury that includes all possible definitions of TBI and head injury (Table 1). The ICD10 codes used are consistent with those selected to explore the NICE guideline's effect on hospital admissions for head injury, with the addition of codes for crush injuries.[13] We selected an ICD10 code subset to define TBI that corresponds to injuries identified by CT imaging including codes for traumatic intra-cranial haemorrhages and skull fractures, but excluded codes for concussion and other clinical diagnoses (Table 1).

Outcomes:

Admissions for head injury:

The monthly number of patients admitted to hospital with one or more ICD10 code indicating head injury between April 1998 and March 2016 was generated by the ISD. As the SIGN guidelines have specific recommendations regarding paediatric patients and patients 65 and over, admissions were stratified into: 0-15, 16-64 and ≥65 age groupings. Yearly and monthly number of admissions were converted into a rate per 100, 000 population using Nomis Office of National Statistics (ONS) mid-year population estimates for Scotland for each age grouping (Supplementary Material 2).

Admissions for Traumatic Brain Injury:

The ISD generated the monthly number of patients admitted to hospital with at least one ICD10 code sub-classification indicting an admission for TBI. Monthly and yearly rates per 100, 000 population of admissions for TBI were calculated using Nomis ONS mid-year Scottish population estimates.

The ISD also provided the monthly number of patients admitted with an ICD10 code indicating TBI that had one or more OPSC4 neurosurgical codes (Table 2). A monthly

For peer review only - http://bmjopen.bmj.com/site/about/guidelines.xhtml

proportion of admissions for TBI that resulted in neurosurgery was estimated. The small number of monthly TBI admissions prevented release of data stratified by age group.

For peer review only

For peer review only - http://bmjopen.bmj.com/site/about/guidelines.xhtml

Table 1: Annual trends admissions for head injury and TBI

Year	Annual rates head injury admissions per 100, 000 population each age group.*				Annual rates TBI admissions per 100, 000 population all age.** (Percentage annual rate all head injury admissions)
	All ages	0-15	16-64	65+	
1999	484	558	453	536	77 (15.9%)
2000	476	519	449	556	70 (14.7%)
2001	493	561	457	578	72 (14.6%)
2002	489	556	447	598	71 (14.5%)
2003	451	521	404	581	76 (16.9%)
2004	435	495	381	589	82 (18.9%)
2005	417	497	350	591	78 (18.7%)
2006	427	477	369	585	79 (18.5%)
2007	442	441	393	653	79 (17.9%)
2008	435	400	386	670	76 (17.4%)
2009	440	406	377	709	79 (18%)
2010	400	361	337	709	80 (20%)
2011	421	369	352	741	91 (21.6%)
2012	411	339	321	817	89 (21.7%)
2013	396	307	293	845	97 (24.5%)
2014	385	319	268	848	102 (26.5%)
2015	373	316	251	867	123 (33%)

*ICD10 codes for head injury: S00-S09, T04.0 and T06.0

** ICD10 codes for TBI: S02.0, S02.1, S02.7, S02.8, S02.9, S06.1, S06.3, S06.4, S06.5, S06.6, S06.7, S06.8, S06.9, T04.0 and T06.0

For peer review only - http://bmjopen.bmj.com/site/about/guidelines.xhtml

Table 2: Impact of the SIGN guidelines on number of admissions and deaths from Traumatic Brain Injury per 100, 000 Scottish population

Outcome	Winter Effect	Initial Trend	1st SIGN Guideline	4-hour Target Introduced	2nd SIGN Guideline	Durbin-Watson Statistic
**Admissions for TBI/100 000**		-0.04 (95% CI: -0.09 to 0.004) P=0.07	**Change level:** 0.26 (95% CI:- 0.74 to 1.26) P=0.61	**Change level:** 0.16 (95% CI: -0.67 to 0.99) P=0.71	**Change level:** -0.39 (95% CI: -1.09 to 0.30) P=0.27	Untransformed 1.46 Prais-Winsten 2.02
			**Change trend:** 0.06 (95% CI: 0.004 to 0.12) P=0.04	**Change trend:** -0.02 (95% CI: -0.05 to 0.01) P=0.24	**Change trend:** 0.05 (95% CI: 0.03 to 0.07) P<0.01	
**Percentage TBI admissions neurosurgical+**		0.05 (95% CI: -0.01 to 0.11) P=0.10	**Change level:** -0.64 (95% CI: -1.83 to 0.56) P=0.29		**Change level:** 0.47 (95% CI:-0.17 to 1.12) P=0.15	Untransformed 1.81
			**Change trend:** -0.06 (95% CI: -0.12 to 0.001) P=0.047		**Change trend:** -0.01 (95% CI:-0.03 to -0.003) P=0.01	
**Deaths/100 000**	0.03 (95% CI: 0.001 to 0.07) P=0.04	-0.001 (95% CI:-0.004 to 0.002) P=0.57	**Change level:** -0.02 (95% CI:- 0.09 to 0.06) P=0.62		**Change level:** -0.01 (95% CI:-0.06 to 0.05) P=0.85	Untransformed 2.3
			**Change trend:** 0.001 (95% CI:-0.002 to 0.005) P=0.44		**Change trend:** 0.0004 (95% CI: -0.001 to 0.001) P=0.46	
**Percentage TBI admissions death**	0.88 (95% CI: 0.36 to 1.41) P<0.01	0.03 (95% CI: -0.03 to 0.10) P=0.35	**Change level:** -0.79 (95% CI: -2.12 to 0.54) P=0.24		**Change level:** 0.52 (95% CI: -0.32 to 1.35) P=0.22	Untransformed 2.08
			**Change trend:** -0.03 (95% CI: -0.10 to 0.03) P=0.33		**Change trend:** -0.03 (95% CI: -0.04 to -0.01) P<0.01	

+ Neurosurgical procedure defined as 1 or more OPSC4 codes: A05.2, A05.3, A05.4, A05.8, A05.9, A40.1, A40.8, A40.9, A41.1, A41.8, A41.9, V03.1, V03.2, V03.4, V03.6, V03.7, V03.8, V03.9, V05.3 and V05.4

For peer review only - http://bmjopen.bmj.com/site/about/guidelines.xhtml

Deaths related to Traumatic Brain Injury:

The ISD provided the monthly number of patients who died within 30 days of admission with at least one ICD10 code for TBI. This was converted into a rate using Nomis ONS mid-year Scottish population estimates. The monthly proportion of patients admitted with TBI who died within 30 days of admission was estimated using the total number of monthly admissions for ICD10 codes that corresponded to TBI.

Statistical analysis:

A monthly time series of the rate of inpatient hospital admissions for head injury ICD codes was plotted from April 1998 to March 2016. An interrupted times series analysis was completed assessing the impact of SIGN head injury guidelines and the 4-hour ED target using the ITSA package in STATA 14.[19 20] The model included 3 intervention time points: the introduction of the 1st SIGN guideline in August 2000, the introduction of the 4-hour target in 2004 and the introduction of the 2nd SIGN guideline in May 2009. Analysis was stratified into 3 age groups: 0-15, 16-64 and ≥65s. A segmented regression model predicting the rate of hospital admissions per 100, 000 population in each age grouping per month was estimated.[15]

Autocorrelation of the residuals was assessed using the Durbin-Watson and Rho statistic.[15 21] Where there was sufficient deviation from a Durbin Watson statistic of 2 and the Rho statistic was not statistically significant, the Prais-Winsten transformation was used to adjust for auto-correlation.[15] Seasonality was assessed by introducing a dummy variable to the model in which winter months (December, January and February) were coded 1 and was included in the model when a statistically significant predictor.

The interrupted time series analysis was repeated to assess: the impact of the SIGN guidelines and 4-hour target on the rate of hospital admissions for patients with an ICD10 code indicating TBI, the impact of SIGN guidelines on the proportion of inpatient admissions for TBI that resulted in neurosurgery or death and the death rate within 30 days of admission for TBI. It was thought a priori that the 4-hour target would not plausibly have affected neurosurgery or deaths. This was confirmed through visual inspection and was excluded from analysis of these outcomes.

For peer review only - http://bmjopen.bmj.com/site/about/guidelines.xhtml

Estimates of the impact of the policy interventions on inpatient admissions were made by using the pre-intervention model to estimate hypothetical monthly rates of admissions if no intervention had occurred. These were subtracted from the monthly admission rates estimated by the post-intervention model.[14] To explore any effect of a policy implementation lag a sensitivity analysis was performed for all the models in which the 12 months immediately following the introduction of a policy change, were excluded from analysis.[15]

Patient and Public Involvement

The Hull and East Yorkshire NHS Trust Trans-Humber Consumer Research Panel and Hull branch of the Headway charity were consulted in the initial stages of developing the research questions addressed in this study. These patient groups highlighted that although national head injury guidelines seemed evidence based, there appeared to be little evidence to show they had achieved their aims.

Results:

Head injury inpatient hospital admissions:

Table 1 show yearly rates of inpatient hospital admissions. Fig. 1 and Table 3 present the results of the interrupted time series assessing the impact of the SIGN guidelines and 4-hour ED target on monthly head injury admissions. Admission rates and estimates of effect are reported per 100, 000 population in each age grouping. The SIGN guidelines and 4-hour target were associated with a significant change in the total level and trend of the rate admissions for head injury (Fig 1a). The effect varied between age group.

0-15 Age Group:

Monthly inpatient admissions fell from 47.18 to 24.02 per 100, 000 per month over the time period (Fig. 1b). Neither SIGN guideline nor the introduction of the 4-hour target significantly affected the underlying reducing trend in hospital admissions but the first guideline was associated with a borderline statistically significant increase in level (6.06; 95% CI: -0.49 to 12.62) (Table 3). Admissions were less likely to occur in winter months (-9.19; 95% CI: -10.81 to -7.57) (Table 3).

16-64 Age Group:

For peer review only - http://bmjopen.bmj.com/site/about/guidelines.xhtml

Inpatient admissions fell from 36.39 to 20.20 per 100, 000 per month from April 1998 to March 2016 (Fig 1c). Before the 1st SIGN guideline hospital admissions were increasing monthly (0.04; 95% CI: -0.08 to 0.15) (Table 3 and Fig. 1c). The 1st guideline was associated with a declining monthly trend in admissions (-0.20; 95% CI:-0.35 to -0.05). The 4-hour target was associated with an initial fall in the number of inpatient admissions (-3.54; 95% CI: -5.76 to -1.33) but subsequent trend of increasing monthly admissions (0.18; 95% CI: 0.10 to 0.27). The 2nd guideline was associated with a return to a declining trend in admissions (-0.18; 95% CI:-0.23 to -0.13). Inpatient admissions were reduced in winter months (-1.74; 95% CI:-2.48 to -1.01).

The trend following the introduction of the 1st SIGN guideline hypothetically continued in the period after the introduction of the 4-hour target is shown in Supplementary Material 3. By subtracting this from the model that incorporated the introduction of the 4-hour target and the 2nd SIGN guideline we estimate that from January 2004 to March 2016 the introduction of the 4-hour target was associated with an additional 745 hospital admissions per 100 000 population aged 16-64.

≥65 Age Group:

Monthly admissions increased from 40.00 to 76.09 over the time period (Fig. 1d). The only statistically significant change in the underlying trend was at the introduction of the 4-hour target which was associated with an acceleration in the increase in hospital admissions (0.15; 95% CI: 0.01 to 0.28) (Table 3 and Fig. 1d). Winter months were associated with increased hospital admissions (1.67; 95% CI: 0.32 to 3.02).

Sensitivity Analysis for 12-month implementation lag:

The introduction of an intervention lag in the model (Supplementary Material 4) did not materially affect estimates of effect associated with interventions.

Inpatient hospital admissions for Traumatic Brain Injury on CT scan:

Admission per 100, 000 per month increased from 6.85 to 10.21 over the time period (Fig. 2). Before the 1st SIGN guideline hospital admissions were decreasing (-0.04; 95% CI:-0.09 to 0.004) (Table 2 and Fig. 2). The introduction of the 1st guideline was associated with a trend of increasing admissions (0.06; 95% CI: 0.004 to 0.12). The 4-hour target was not associated

For peer review only - http://bmjopen.bmj.com/site/about/guidelines.xhtml

with a significant change in level or trend. The introduction of the 2nd SIGN guideline was associated with an acceleration in the increase in admissions (0.05; 95% CI: 0.04 to 0.06).

Comparing admissions for TBI that would have occurred if the 2nd SIGN guideline had not been introduced with the empirically derived model indicates that from May 2009 to March 2016 the introduction of the 2nd SIGN guideline was associated with an additional 138 hospital admissions per 100, 000 population (Supplementary Material 5).

For peer review only

For peer review only - http://bmjopen.bmj.com/site/about/guidelines.xhtml

Table 3: Impact of the SIGN guidelines and introduction of 4-Hour ED Target on number of Head Injury Admissions per 100, 000 Scottish population by age group

Age Band	Winter Effect	Initial Trend	1st SIGN Guideline	4-hour Target Introduced	2nd SIGN Guideline	Durbin-Watson Statistic
**All ages**	-3.00 (95% CI:-3.78 to -2.30) P<0.01	0.04 (95% CI: -0.08 to 0.15) P=0.53	**Change level:** 2.45 (95% CI:0.09 to 4.83) P=0.04	**Change level:** -2.89 (95% CI:-4.84 to -0.95) P<0.01	**Change level:** -0.84 (95% CI:-2.50 to 0.78) P=0.31	Untransformed 1.68 Prais-Winsten 2.02
			**Change trend:** -0.14 (95% CI: -0.27 to -0.01) P=0.03	**Change trend:** 0.13 (95% CI: 0.06 to 0.20) P<0.01	**Change trend:** -0.09 (95% CI:-0.13 to -0.05) P<0.01	
**0-15**	-9.19 (95% CI: -10.81 to -7.57) P<0.01	-0.21 (95% CI: -0.53 to 0.12) P=0.22	**Change level:** 6.06 (95% CI:-0.49 to 12.62) P=0.07	**Change level:** -0.41 (95% CI: -5.98 to 5.17) P=0.89	**Change level:** -0.54 (95% CI:-5.31 to 4.22) P=0.82	Untransformed 1.34 Prais-Winsten 1.87
			**Change trend:** -0.10 (95% CI:-0.29 to 0.49) P=0.61	**Change trend:** -0.05 (95% CI: -0.27 to 0.16) P=0.63	**Change trend:** 0.06 (95% CI:-0.07 to 0.18) P=0.37	
**16-64**	-1.75 (95% CI:-2.48 to -1.01) P<0.01	0.06 (95% CI: -0.07 to 0.19) P=0.39	**Change level:** 2.12 (95% CI:-0.54 to 4.79) P=0.12	**Change level:** -3.54 (95% CI:-5.76 to -1.33) P<0.01	**Change level:** -0.87 (95% CI: -2.74 to 0.99)P=0.36	Untransformed 1.45 Prais-Winsten 2.10
			**Change trend:** -0.20 (95% CI: -0.35 to -0.05) P<0.01	**Change trend:** 0.18 (95% CI: -0.10 to 0.27) P<0.01	**Change trend:** -0.18 (95% CI:-0.23 to -0.13)P<0.01	
**65+**	1.67 (95% CI: 0.32 to 3.02) P=0.02	0.17(95% CI: -0.04 to 0.39) P=0.11	**Change level:** 2.28 (95% CI: -2.13 to 6.86) P= 0.30	**Change level:** -2.70 (95% CI: -6.39 to 0.99) P=0.15	**Change level:** 0.85 (95% CI:-2.22 to 3.93)P=0.59	Untransformed 1.70 Prais-Winsten 2.00
			**Change trend:** -0.16 (95% CI: -0.40 to 0.09) P=0.21	**Change trend** 0.15 (95% CI: 0.01 to 0.28) P=0.03	**Change trend:** 0.05 (95% CI: -0.03 to 0.12) P=0.23	

For peer review only - http://bmjopen.bmj.com/site/about/guidelines.xhtml

Both SIGN guidelines were associated with a reduction in trend in the percentage of TBI admissions resulting in neurosurgery: 1st guideline (-0.06; 95% CI: -0.12 to 0.001) and 2nd guideline (-0.01; 95% CI: -0.03 to -0.003) (Table 2 and supplementary material 6). A 12-month implementation lag did not materially affect the estimates (Supplementary Material 7).

Deaths following admission for TBI:

Neither guideline was associated with a change in level or trend in monthly death rate within 30 days of admission with an ICD 10 code indicating TBI (Table 2 and Supplementary Material 8). The introduction of the 2nd SIGN guideline was associated with a significant reduction in the underlying trend in the monthly percentage of inpatient admissions for TBI that resulted in death within 30 days (-0.03; 95% CI: -0.04 to -0.01) (Table 2 and Supplementary Material 9).

Introduction of a 12-month implementation lag into the models did not materially change the estimates (Supplementary Material 10).

Discussion:

Summary:

To our knowledge, this is the first study to evaluate the impact of the SIGN guidelines and the 4-hour ED target on head injury hospital admissions. We found evidence that the SIGN guidelines reduced inpatient admissions, the effect varying by age group. In the 16-64 age group both guidelines were associated with a reduction in hospital admissions (Fig. 1c and Table 3). This may be due to increased CT imaging in the ED identifying more patients without TBI safe for discharge. In the paediatric population there was an underlying trend of reducing hospital admissions that the guidelines did not appear to affect. In the ≥65 age group neither SIGN guideline iteration acted to offset the secular trend of increasing hospital admissions. Inpatient admissions increased in winter months in the ≥65 age group but were reduced in other age groups. Falls from standing are known to be the most common cause of brain injuries in those ≥65, whilst assault and road traffic accidents are more common causes of injuries in younger patients.[22] Weather conditions during winter

For peer review only - http://bmjopen.bmj.com/site/about/guidelines.xhtml

may increase the likelihood of falls from standing whilst reducing the prevalence of assault or road traffic accidents.[23]

The introduction of both SIGN guideline iterations were associated with an increased in hospital admissions for patients with TBI and a reduction in the proportion of inpatient admissions that resulted in neurosurgery or death (Table 2). The guidelines may have acted, as previously hypothesised, to increase CT diagnosis and admissions of patients with brain injuries of lower severity who do not require intervention.[13] The 4-hour target was associated with an increase in hospital admissions for adults (Fig. 1c, 1d and Table 3). This effect was reversed by the 2nd SIGN guideline in the 16-64 but not the ≥65 age group.

The 4-hour target's effect on adult head injury hospital admissions appears related to the time from admission which CT imaging is recommended . The 1st guideline contained no recommendation for when CT imaging should occur. The introduction of the 4- hour ED target increased head injury hospital admissions in both the 16-64 and ≥65 age groupings, presumably as patients were admitted to await imaging to comply with the target. The 2nd SIGN guideline increased the indications for immediate CT imaging in the 16-64 age group and was associated with a downward trend in admissions. A reduction in hospital admissions was not observed in the ≥65 age group following the 2nd SIGN guideline. The 2nd SIGN guideline includes a series of specific additional indications for imaging in the ≥65 age group that are recommended to occur within 8 hours of ED attendance. As 8 hours is longer than the 4-hour target patients with these indications for CT imaging would be admitted to hospital. This may account for why the 2nd SIGN guideline was not associated with reduced hospital admissions for those ≥65.

Strengths:

We have used a time series of 216 data points and followed established techniques to control for seasonal factors and auto-correlation.[15] We have adjusted for population factors using mid-year population estimates that incorporate the changing demography of Scotland's population. The models constructed were robust to sensitivity analysis for time lags.

There is controversy regarding which ICD10 codes correspond to clinical definitions of head injury and TBI with inconsistent sets of ICD10 codes used to encompass both.[24] We selected

For peer review only - http://bmjopen.bmj.com/site/about/guidelines.xhtml

subsets of codes for head injury that are likely to be sensitive to changes in admission practice related to increased diagnostic precision of TBI from increased CT imaging and inpatient admissions of patients awaiting an exclusion of TBI due to the 4-hour target. Our ICD10 code selection for TBI was intended to encompass radiologically detected injuries as this outcome would be sensitive to increased diagnoses of TBI by CT imaging.

Weaknesses:

Ideally the effects of guideline implementation and policy interventions would be assessed using randomised control trials. However, interrupted time series analysis (a rigorous quasi-experimental study design) is becoming increasingly popular particularly for the evaluation of health care practice, programmes and policy because it allows causal inferences when interventions are introduced at specific time points. Discontinuities in outcomes, observed at or shortly after the time of intervention, constitute persuasive evidence of an effect with high internal and external validity.[14 15 25 26] The method has limitations notably the potential for observed discontinuities to result from co-interventions instead of the health policies under investigation. We cannot find other policies or sudden changes to the population of Scotland that could account for the observed changes in admissions for head injury in Scotland at the time of the either the introduction of the SIGN guidelines or the 4-hour ED target.

This study used routinely collected Scottish Information Services Division (ISD) data, and administrative data should be approached with some caution.[27] There may be inaccuracies in diagnostic codes due to coding errors. However, there were no changes to the cohort of admitted patients that data was collected on during the study period and ISD data has been found to be both sufficiently reliably and comprehensively collected to support its use in research.[28 29] Furthermore, random poor coding, without changes in coding practice, are unlikely to result in sharp discontinuities at the specific time points of the policies considered here. ICD coding changed in 1996, so we only used ISD data from 1998 to give time for adjustment. This limited the number of data points before the 1st SIGN guideline, so we may have lacked the power to detect some changes associated with the 1st guideline as statistically significant (Table 3 and Fig. 1).

For peer review only - http://bmjopen.bmj.com/site/about/guidelines.xhtml

We could not stratify all the analysis by age group as the small number of some outcomes prevented release of aggregated data. A sensitivity analysis using ICD10 injury subtypes was also not possible due to the aggregated nature of the available data. A more sensitive outcome measure for changes in admission practice due to either the SIGN guidelines or 4-hour target may be the proportion of attendances to the ED following head injury that result in inpatient hospital admission. TBI has become more common in the elderly and if analysis of the proportion of ED attendances that resulted in inpatient admission was stratified by age this would account for age group differences in incidence of injuries .[30] We were unable to differentiate Clinical Decision Unit admissions from other types of inpatient admissions in our data. However, the extent to which Clinical Decision Unit admissions in the UK represent materially different and more cost-effective care compared to other types of hospital admissions and should be treated differently is debatable.[31]Furthermore, only 6 hospitals in Scotland had Clinical Decision Units during the time period of our study and were not established at the same time as the policies considered here.[32]

There is evidence that deaths from severe TBI fell following the introduction of the NICE guidelines.[33] We may have missed this effect due to the undifferentiated cohort of TBI patients used to assess deaths and the use of all-cause mortality. We also could not adjust mortality by age or injury severity.

Estimates of the impact of guidelines will depend on the extent to which the guidelines have been implemented. There are no national audit data on this, though one local audit conducted in 2001 indicated less than half of patients the SIGN guidelines deemed were safe for discharge from the ED were actually discharged.[34] Our study may therefore underestimate the potential impact of full implementation.

Comparison to previous literature:

A study that assessed the NICE head injury guideline's impact using English data (although not using an interrupted time series) found that increased CT imaging led to an increase in hospital admissions, contrary to expectations.[13] Increased admissions following the introduction of the NICE guidelines could be due to increased diagnosis of TBI due to more CT imaging or the effect of the 4-hour ED target.[13] We found evidence that both factors increased hospital admissions in Scotland.

For peer review only - http://bmjopen.bmj.com/site/about/guidelines.xhtml

Analysis of English HES data from 2000-2011 found yearly paediatric hospital admissions for head injury increased from 34 to 37 inpatient admissions per 10 000 children.[35] We found Scottish paediatric admissions fell from 56 per 10 000 children in 1999 to 32 per 10 000 children in 2015. There is evidence that clinicians are less likely to implement head injury guidelines in children.[35-37] A lack of implementation may explain why the SIGN guidelines were not associated with a reduction in paediatric hospital admissions.

Implications:

The overall effect of the SIGN guidelines was to reduce inpatient hospital admissions for head injured patients and this supports previous research indicating early CT imaging may represent a cost-effective management strategy.[9] However, the guidelines were associated with increased inpatient admissions for patients with TBI, possibly resulting from increased imaging identifying more TBI of lower clinical severity. Research better characterising the risk associated with TBI identified by CT imaging could help identify a sub-set of low-risk patients who could be safely discharged from the ED. This could help mitigate the increase in inpatient hospital admissions in this group associated with the SIGN and other similar guidelines.

We found evidence that the 4-hour ED target acted to reverse reductions in hospital admissions in the 16-64 age group associated with the SIGN guidelines. As has been previously argued, performance targets need to be carefully considered before implementation to ensure that they do not have unintended consequences, in this case undermining the benefits of evidence-based clinical guidelines.[38] A more granular approach to the 4-hour ED target that reflects condition specific clinical circumstances, such as the time frame of CT imaging in head injury, could help to prevent such costly unintended consequences.

Given the limitations in the mortality analysis undertaken it is hard to draw conclusions about how effective the SIGN guidelines were at reducing deaths from TBI. Future analysis should attempt to adjust for age and severity of injury and this will require patient level data.

Conclusion:

For peer review only - http://bmjopen.bmj.com/site/about/guidelines.xhtml

Increased early CT imaging of head injured patients may reduce hospital admissions. However, this effect may be offset by an increase in the diagnosis of TBI of lower severity and the 4-hour ED target. Future research should aim to better risk stratify patients with TBI identified by CT imaging to help reduce hospital admissions related to increased CT imaging. Care should also be taken when introducing arbitrary performance targets, such as the 4-hour target, to ensure they do not undermine the beneficial effect of clinical guidelines.

Acknowledgements:

Sir Graham Teasdale commented on the manuscript and provided invaluable historical context and knowledge regarding the use of administrative data sets in traumatic brain injury research. The Hull and East Yorkshire NHS Trust Trans-Humber Consumer Research Panel and Hull branch of the Headway charity helped develop the research questions addressed in this study.

Competing interests:

The authors declare that they have no competing interests.

Funding:

Carl Marincowitz is funded by a National Institute for Health Research Doctoral Fellowship (DRF-2016-09-086). This study presents independent research funded by the National Institute for Health Research (NIHR). The views expressed are those of the author(s) and not necessarily those of the NHS, the NIHR or the Department of Health.

Authors' contributions:

This idea for the study was conceived by Carl Marincowitz with help from Trevor Sheldon, Fiona Lecky and Victoria Allgar. The analysis was completed by Carl Marincowitz and Eleanor Morris with specialist advice regarding interrupted time series analysis from Trevor Sheldon and Victoria Allgar. Fiona Lecky provided specialist advice regarding the clinical context and interpretation of the results. All authors read and approved the final manuscript.

Data Sharing:

For peer review only

For peer review only - http://bmjopen.bmj.com/site/about/guidelines.xhtml

The data used for this analysis was purchased for a fee from Information Service Division Scotland. The corresponding author is happy to share the exact details of the data extract and how to acquire it from ISD Scotland..

Figures:

Figure 1: The Impact of the 4-hour target and SIGN guidelines on head injury admissions

Figure 2: The impact of the SIGN guidelines and 4-Hour Target on admissions for Traumatic Brain Injury

References:

1. NICE. National Clinical Guidance Centre. (2014). CG 176 Head Injury Triage, assessment, investigation and early management of head injury in children, young people and adults. National Institute for Health and Care Excellence. In: NICE, ed. UK: DOH, 2014.
2. SIGN Guideline 110: Early management of a patient with a head injury: a national clinical guideline. 2009.
3. Miller JD. Minor, moderate and severe head injury. *Neurosurg Rev* 1986;9(1-2):135-9.
4. Kim J, Kemp S, Kullas K, et al. Injury patterns in patients who "talk and die". *J Clin Neurosci* 2013;20(12):1697-701. doi: 10.1016/j.jocn.2013.02.021
5. af Geijerstam JL, Oredsson S, Britton M, et al. Medical outcome after immediate computed tomography or admission for observation in patients with mild head injury: randomised controlled trial. *BMJ* 2006;333(7566):465. doi: 10.1136/bmj.38918.669317.4F
6. Stiell IG, Wells GA, Vandemheen K, et al. The Canadian CT Head Rule for patients with minor head injury. *Lancet* 2001;357(9266):1391-6.
7. SIGN Guideline 46: Early Management of Patients with a Head Injury. 2000
8. Shivaji T, Lee A, Dougall N, et al. The epidemiology of hospital treated traumatic brain injury in Scotland. *BMC Neurology* 2014;14(1):2. doi: 10.1186/1471-2377-14-2
9. Pandor A, Goodacre S, Harnan S, et al. Diagnostic management strategies for adults and children with minor head injury: a systematic review and an economic evaluation. *Health Technol Assess* 2011;15(27):1-202. doi: 10.3310/hta15270
10. Dunning J, Daly JP, Malhotra R, et al. The implications of NICE guidelines on the management of children presenting with head injury. *Arch Dis Child* 2004;89(8):763-7. doi: 10.1136/adc.2003.042523
11. Hassan Z, Smith M, Littlewood S, et al. Head injuries: a study evaluating the impact of the NICE head injury guidelines. *Emerg Med J* 2005;22(12):845-9. doi: 10.1136/emj.2004.021717
12. GENERAL DoHCAA. Improving Emergency Care in England. *National Audit Office* 2004;HC 1075 Session 2003-2004
13. Goodacre S. Hospital admissions with head injury following publication of NICE guidance. *Emerg Med J* 2008;25(9):556-7. doi: 10.1136/emj.2007.055723
14. Wagner AK, Soumerai SB, Zhang F, et al. Segmented regression analysis of interrupted time series studies in medication use research. *J Clin Pharm Ther* 2002;27(4):299-309.
15. Lopez Bernal J, Cummins S, Gasparrini A. Interrupted time series regression for the evaluation of public health interventions: a tutorial. *Int J Epidemiol* 2016 doi: 10.1093/ije/dyw098 [published Online First: 2016/06/11]
16. http://apps.who.int/classifications/icd10/browse/2010/en [

For peer review only - http://bmjopen.bmj.com/site/about/guidelines.xhtml

17. National Clinical Coding Standards - OPCS-4 (2017) Reference Book.
18. http://www.isdscotland.org/Products-and-Services/Terminology-Services/Coding-and-Terminology-Systems/ [
19. Penfold RB, Zhang F. Use of interrupted time series analysis in evaluating health care quality improvements. *Acad Pediatr* 2013;13(6 Suppl):S38-44. doi: 10.1016/j.acap.2013.08.002 [published Online First: 2013/12/07]
20. Linden A. Conducting interrupted time-series analysis for single- and multiple-group comparisons. *The Stata Journal* 2015;15(2):480-500.
21. Hamilton I, Lloyd C, Hewitt C, et al. Effect of reclassification of cannabis on hospital admissions for cannabis psychosis: a time series analysis. *Int J Drug Policy* 2014;25(1):151-6. doi: 10.1016/j.drugpo.2013.05.016 [published Online First: 2013/07/23]
22. Lawrence T, Helmy A, Bouamra O, et al. Traumatic brain injury in England and Wales: prospective audit of epidemiology, complications and standardised mortality. *BMJ Open* 2016;6(11):e012197. doi: 10.1136/bmjopen-2016-012197
23. Rock DJ, Judd K, Hallmayer JF. The seasonal relationship between assault and homicide in England and Wales. *Injury* 2008;39(9):1047-53. doi: https://doi.org/10.1016/j.injury.2008.03.025
24. Chen AY, Colantonio A. Defining neurotrauma in administrative data using the International Classification of Diseases Tenth Revision. *Emerg Themes Epidemiol* 2011;8(1):4. doi: 10.1186/1742-7622-8-4 [published Online First: 2011/05/17]
25. Kontopantelis E, Doran T, Springate DA, et al. Regression based quasi-experimental approach when randomisation is not an option: interrupted time series analysis. *BMJ* 2015;350:h2750. doi: 10.1136/bmj.h2750
26. Barnighausen T, Tugwell P, Rottingen JA, et al. Quasi-experimental study designs series-paper 4: uses and value. *J Clin Epidemiol* 2017;89:21-29. doi: 10.1016/j.jclinepi.2017.03.012 [published Online First: 2017/04/04]
27. Hand DJ. Statistical challenges of administrative and transaction data. *J R Statist Soc A* 2018 (pre-publication presented at The Royal Statistical Society);181(Part 3):1-24.
28. Burns EM, Rigby E, Mamidanna R, et al. Systematic review of discharge coding accuracy. *Journal of Public Health* 2012;34(1):138-48. doi: 10.1093/pubmed/fdr054
29. Harley K, Jones C. Quality of Scottish Morbidity Record (SMR) data. *Health Bull (Edinb)* 1996;54(5):410-7. [published Online First: 1996/09/01]
30. Woodford et al. MAJOR TRAUMA IN OLDER PEOPLE. *The Trauma and Audit Research Network Report* 2017
31. Cooke MW, Higgins J, Kidd P. Use of emergency observation and assessment wards: a systematic literature review. *Emerg Med J* 2003;20(2):138-42. [published Online First: 2003/03/19]
32. Office SNA. www.audit-scotland.gov.uk/docs/health/2010/nr_100812_emergency_departments.rtf 2010 [
33. Fuller G, Bouamra O, Woodford M, et al. Temporal trends in head injury outcomes from 2003 to 2009 in England and Wales. *Br J Neurosurg* 2011;25(3):414-21. doi: 10.3109/02688697.2011.570882
34. Kerr J, Smith R, Gray S, et al. An audit of clinical practice in the management of head injured patients following the introduction of the Scottish Intercollegiate Guidelines Network (SIGN) recommendations. *Emerg Med J* 2005;22(12):850-4. doi: 10.1136/emj.2004.022673 [published Online First: 2005/11/22]
35. Marlow R, Mytton J, Maconochie IK, et al. Trends in admission and death rates due to paediatric head injury in England, 2000-2011. *Arch Dis Child* 2015;100(12):1136-40. doi: 10.1136/archdischild-2015-308615
36. Plumb J. 'My 15-month-old child fell over and bumped his head doctor. He's fine now but he's been sick 3 times since it happened'. "Oh dear, that's not so NICE". *Br J Neurosurg*

For peer review only

2009;23(6):574; author reply 75. doi: 10.3109/02688690903419115 [published Online First: 2009/11/20]

37. Mooney JS, Yates A, Sellar L, et al. Emergency head injury imaging: implementing NICE 2007 in a tertiary neurosciences centre and a busy district general hospital. *Emerg Med J* 2011;28(9):778-82. doi: 10.1136/emj.2009.083360

38. Sheldon T. Promoting health care quality: what role performance indicators? *Qual Health Care* 1998;7 Suppl:S45-50. [published Online First: 1999/05/29]

For peer review only

For peer review only - http://bmjopen.bmj.com/site/about/guidelines.xhtml

Figure 1:

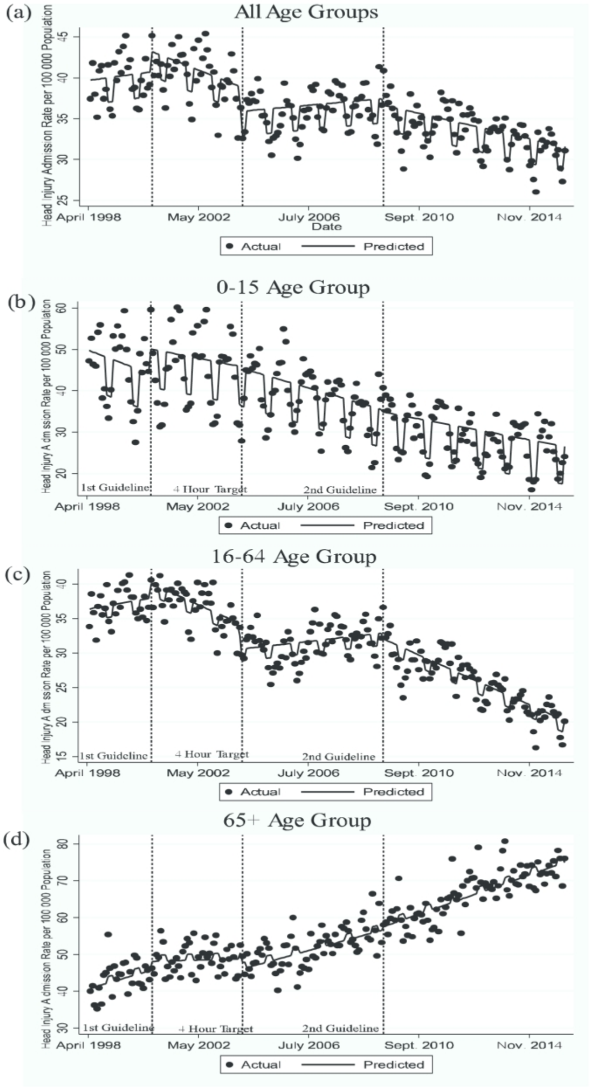

Figure 1: The Impact of the 4-hour target and SIGN guidelines on head injury admissions

210x246mm (300 x 300 DPI)

Figure 2:

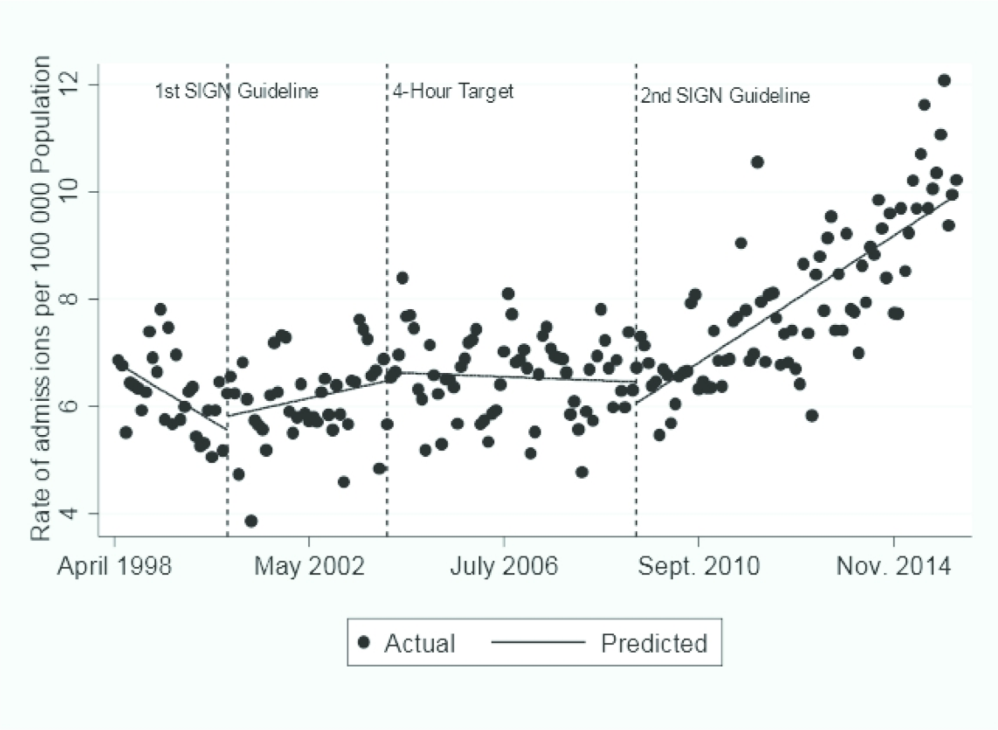

Figure 2: The impact of the SIGN guidelines and 4-Hour Target on admissions for Traumatic Brain Injury

206x159mm (300 x 300 DPI)

Supplementary Material 1: Summary of Health policies assessed

Policy	Time of Introduction	Key Features
1st SIGN Head Injury Guideline	August 2000	Indication for CT imaging (referenced directly from 2000 SIGN guideline):<br>(1) GCS<13<br>(2) A deteriorating level of consciousness or progressive focal neurological signs<br>(3) Confusion or drowsiness (CGS 13 or 14/15) followed by failure to improve within at most four hours of clinical observation<br>(4) Radiological/clinical evidence of a fracture, whatever the level of consciousness<br>(5) New focal neurological signs which are not getting worse<br>(6) Full consciousness (GCS 15/15) with no fracture but other features, including:<br>– severe and persistent headache<br>– nausea and vomiting<br>– irritability or altered behaviour<br>– a seizure.<br><br>Skull films should be carried out if any of the following apply and if CT is not being performed:<br>If the patient is alert and orientated and obeying commands (GCS 15/15) but:<br>– the mechanism of injury has not been trivial; or<br>– consciousness has been lost; or<br>– the patient has loss of memory or has vomited; or<br>– the scalp has a full thickness laceration or a boggy haematoma; or<br>– the history is inadequate.
4-Hour ED performance target	2004	98% of patients attending the ED to be assessed, treated and either discharged or admitted to hospital within 4 hours of arrival. Financial incentives associated with meeting the target.
2nd Sign Head Injury Guideline	May 2009	Referenced directly from 2nd SIGN Guidelines<br><br>Indications Immediate CT scanning (adult):<br>- GCS<13/15<br>- GCS 13/15 or 14/15 followed by failure to improve within one hour of clinical observation<br>- base of skull or depressed skull fracture and/or suspected penetrating injuries<br>- a deteriorating level of consciousness or new focal neurological signs<br>- GCS 15/15 with severe and persistent headache or two episodes of vomiting<br>- coagulopathy (eg warfarin use) and loss of consciousness or amnesia<br>Indications CT scanning within eight (adult):<br>- age>65 (with loss of consciousness or amnesia)

For peer review only

		- clinical evidence of a skull fracture but no clinical features indicative of an immediate CT scan<br>- any seizure activity<br>- significant retrograde amnesia (>30 minutes)<br>- dangerous mechanism of injury<br><br>Indications Immediate CT scanning (<16 years):<br>- GCS≤13 on assessment in ED<br>- witnessed loss of consciousness >5 minutes<br>- suspicion of open or depressed skull injury or tense fontanelle<br>- focal neurological deficit<br>- any sign of basal skull fracture.<br>Indications CT scanning within 8 hours (<16 years):<br>- presence of any bruise/swelling/laceration >5 cm on the head<br>- post-traumatic seizure<br>- amnesia (anterograde or retrograde) lasting >5 minutes<br>- clinical suspicion of non-accidental head injury<br>- a significant fall<br>- age under one year: GCS<15 in emergency department<br>- three or more discrete episodes of vomiting<br>- abnormal drowsiness.

For peer review only

Supplementary Material 2: Changes to Scotland's population structure

i) Mid-year Estimate Scottish Population 0-15

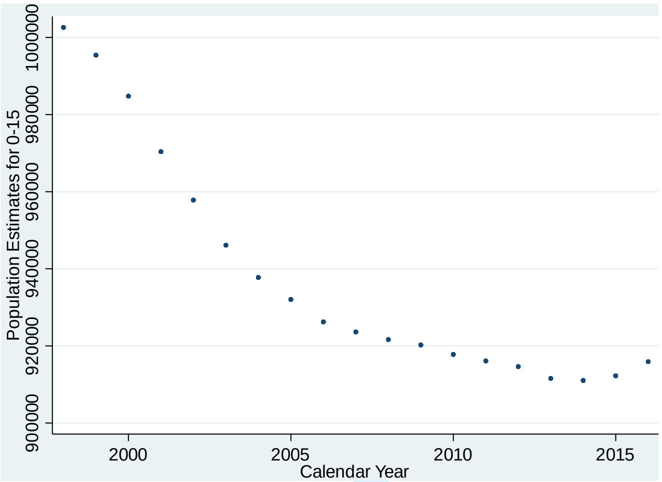

ii) Mid-year Estimate Scottish population 16-64

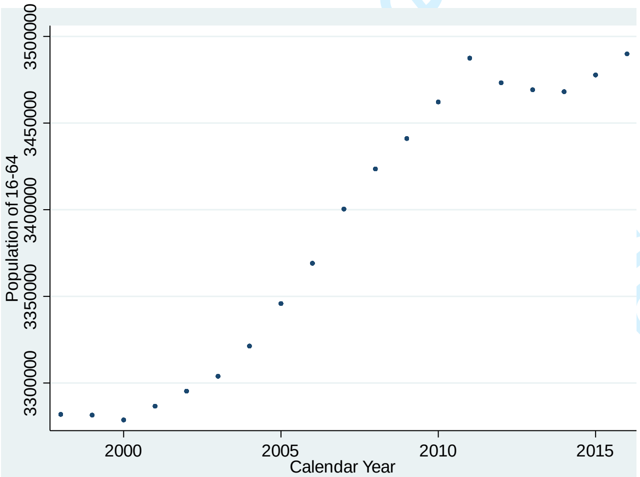

iii) Mid-year Estimate Scottish population 65+

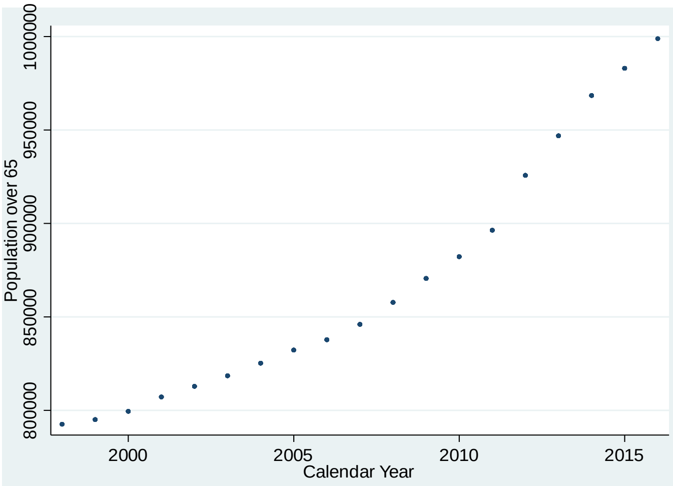

Supplementary Material 3: Increase in admissions related to the introduction of the 4-hour target in the 16-64 age group

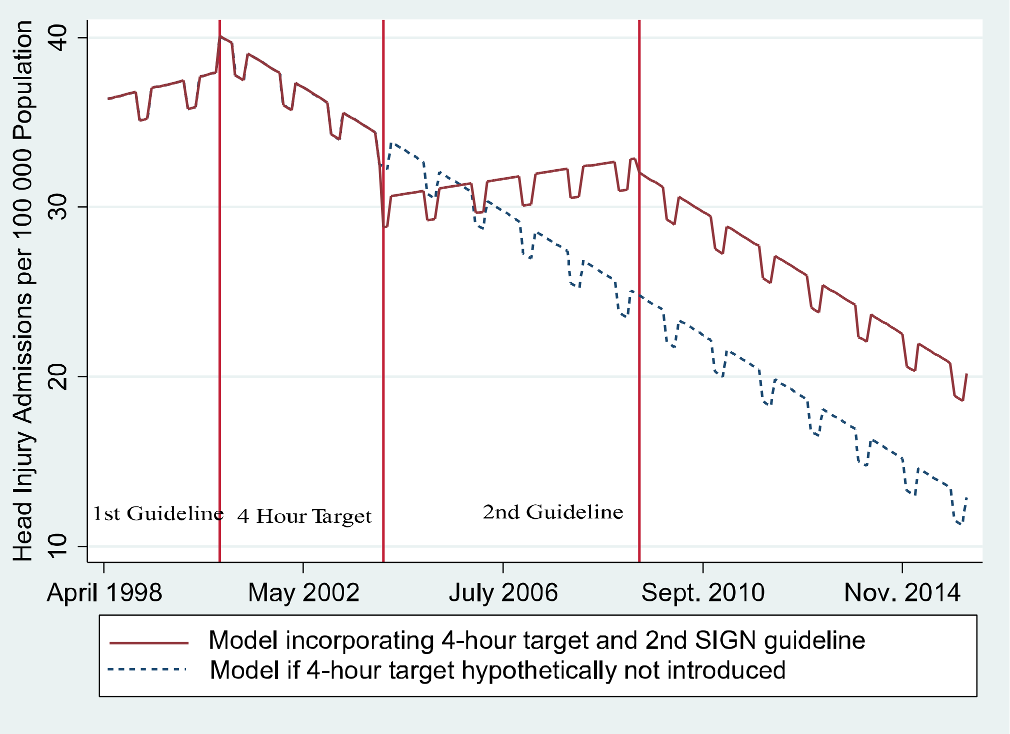

For peer review only

Supplementary Material 4: Estimate of Impact of the SIGN guidelines and introduction of 4-Hour Target admissions for head injury with 12-month time lag

**Age Band**	**Winter**	**Initial Trend**	**1st SIGN Guideline**	**4-hour Target Introduced**	**2nd SIGN Guideline**	**Durbin-Watson Statistic**
**All ages**	-3.00 (95% CI: -3.75 to -2.26) P<0.01	0.04 (95% CI: -0.07 to 0.14) P=0.48	**Change level:** 2.66 (95% CI:0.27 to 5.04) P=0.03	**Change level:** -2.73 (95% CI:-4.85 to -0.61) P<0.01	**Change level:** -2.07 (95% CI:-3.74 to -0.40) P=0.02	Untransformed 1.77 Prais-Winsten 2.00
			**Change trend:** -0.23 (95% CI:-0.38 to -0.08) P<0.01	**Change trend:** 0.25 (95% CI: 0.14 to 0.36) P<0.01	**Change trend:** -0.13 (95% CI: -0.18 to -0.08) P<0.01	
**0-15**	-9.31 (95% CI: -11.05 to -7.59) P<0.01	-0.18 (95% CI:-4.89 to 0.13) P=0.26	**Change level:** 8.53 (95% CI:1.93 to 15.13) P=0.01	**Change level:** 1.61 (95% CI:-4.39 to 7.61) P=0.60	**Change level:** -1.71 (95% CI:-6.61 to 3.20) P=0.49	Untransformed 1.37 Prais-Winsten 1.85
			**Change trend:** -0.17 (95% CI:-0.61 to 0.28) P=0.47	**Change trend:** 0.15 (95% CI: -0.17 to 0.48) P=0.36	**Change trend:** 0.10 (95% CI:-0.50 to 0.26) P=0.18	
**16-64**	-1.80 (95% CI:-2.56 to -1.04) P<0.01	0.06 (95% CI:-0.06 to 0.17) P=0.34	**Change level:** 1.29 (95% CI:-1.28 to 3.85) P=0.32	**Change level:** -4.19 (95% CI:-6.48 to -1.89) P<0.01	**Change level:** -3.06 (95% CI:-4.88 to -1.24) P<0.01	Untransformed 1.57 Prais-Winsten 2.05
			**Change trend:** -0.25 (95% CI:-0.41 to -0.09) P<0.01	**Change trend:** 0.29 (95% CI: -0.17 to 0.40) P<0.01	**Change trend:** -0.25 (95% CI:-0.30 to -0.19) P<0.01	
**65+**	1.69 (95% CI: 0.22 to 3.17) P=0.03	0.17 (95% CI:-0.04 to 0.38) P= 0.10	**Change level:** 3.64 (95% CI:-1.08 to 8.36) P= 0.13	**Change level:** -1.64 (95% CI:-5.83 to 2.55) P=0.44	**Change level:** 2.30 (95% CI:-1.00 to 5.60) P=0.17	Untransformed 1.78 Prais-Winsten 2.00
			**Change trend:** -0.22 (95% CI:-0.51 to 0.07) P=0.14	**Change trend** 0.26 (95% CI: 0.04 to 0.48) P=0.02	**Change trend:** 0.003 (95% CI:-0.10 to 0.10) P=0.95	

For peer review only

Supplementary Material 5: Increase in admissions related to the introduction of the 2nd SIGN guideline

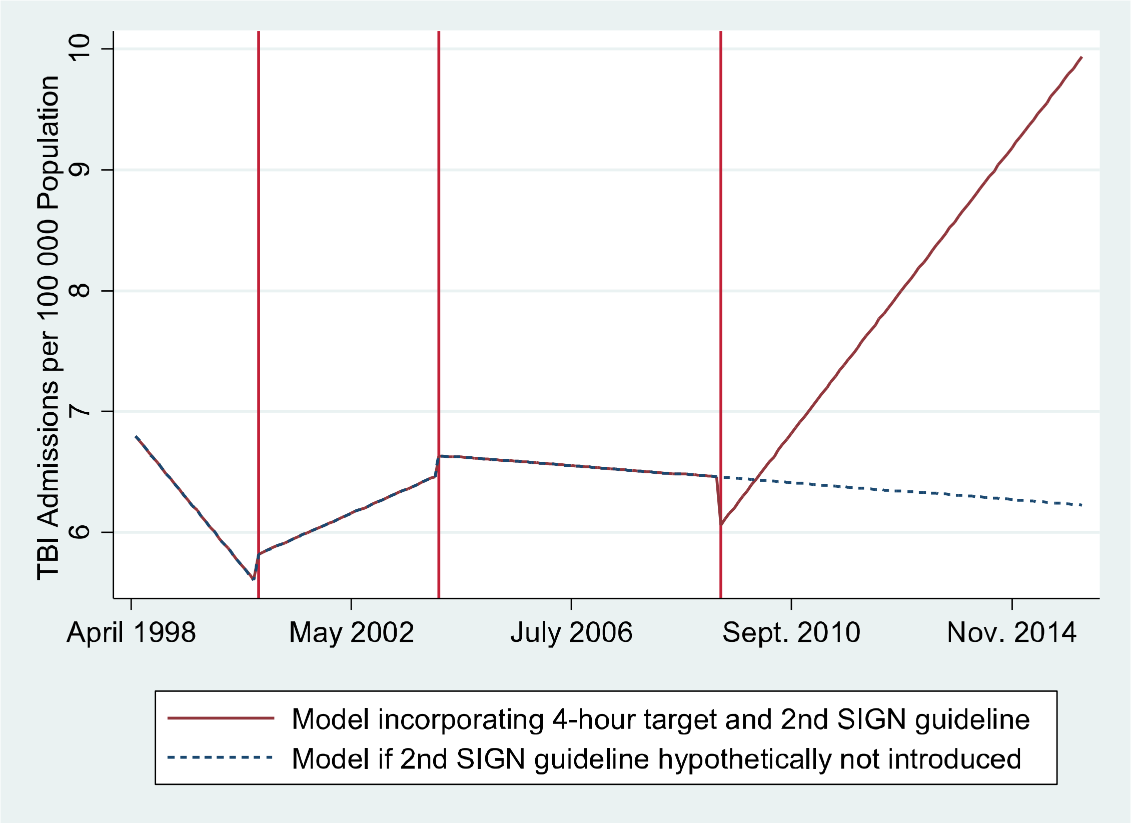

Supplementary Material 6: The impact of the SIGN guidelines on the percentage of admissions for TBI that resulted in neurosurgery

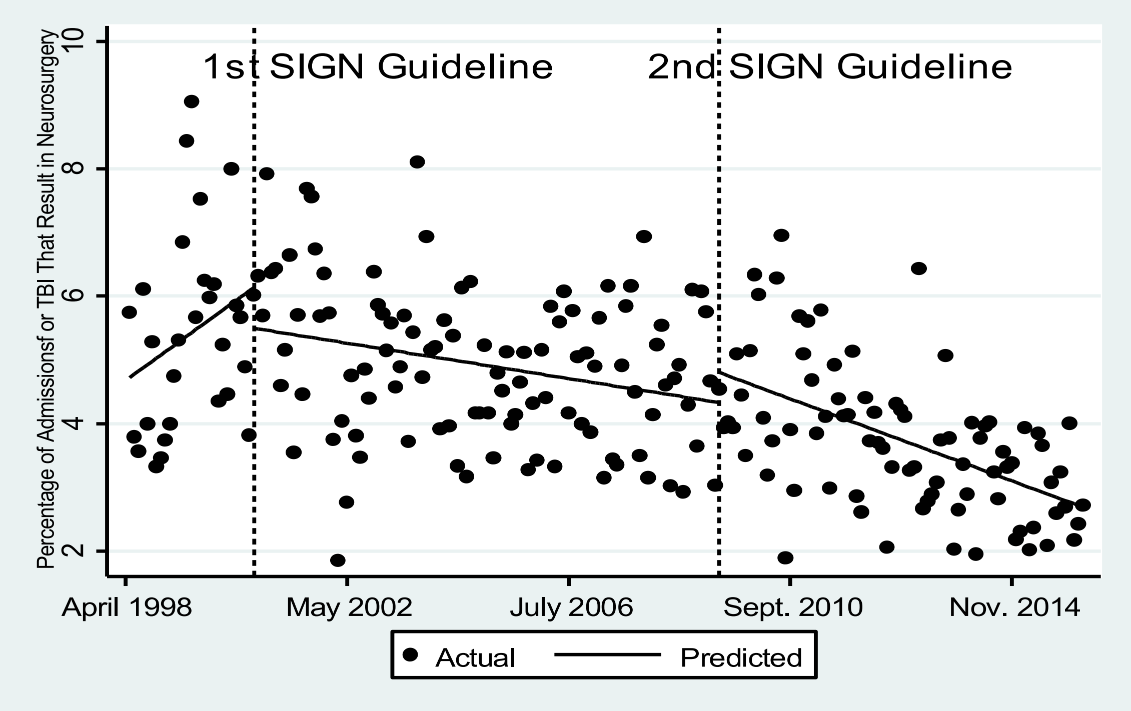

Supplementary Material 7: Estimate of impact of the SIGN guidelines and 4-Hour Target on admissions for Traumatic Brain Injury with 12-month time lag

**Outcome**	**Initial Trend**	**1st SIGN Guideline**	**4-hour Target Introduced**	**2nd SIGN Guideline**	**Durbin-Watson Statistic**
**Admissions for TBI/ 100 000**	-0.05 (95% CI: -0.10 to 0.003) P=0.07	**Change level:** 0.75 (95% CI:-0.34 to 1.84) P=0.18	**Change level:** 0.35 (95% CI:-0.63 to 1.33) P=0.48	**Change level:** -0.16 (95% CI: -0.92 to 0.60) P=0.69	Untransformed 1.44 Prais-Winsten 2.03
		**Change trend:** 0.05 (95% CI:-0.02 to 0.12) P=0.16	**Change trend:** -0.01 (95% CI:-0.06 to 0.04) P=0.66	**Change trend:** 0.05 (95% CI:3 to 0.08) P<0.01	
**Percentage TBI admissions neurosurgical**	0.05 (95% CI: -0.01 to 0.11) P=0.10	**Change level:** -0.96 (95% CI:-2.19 to 0.28) P=0.13		**Change level:** 0.17 (95% CI:-0.59 to 0.93) P=0.66	Untransformed 1.85
		**Change trend:** -0.06 (95% CI:-0.12 to 0.003) P=0.06		**Change trend:** -0.02 (95% CI:-0.04 to-0.004) P=0.01	

For peer review only

Supplementary Material 8: The impact of the SIGN guidelines on deaths per 100 000 population within 30 days of admission with traumatic brain injury

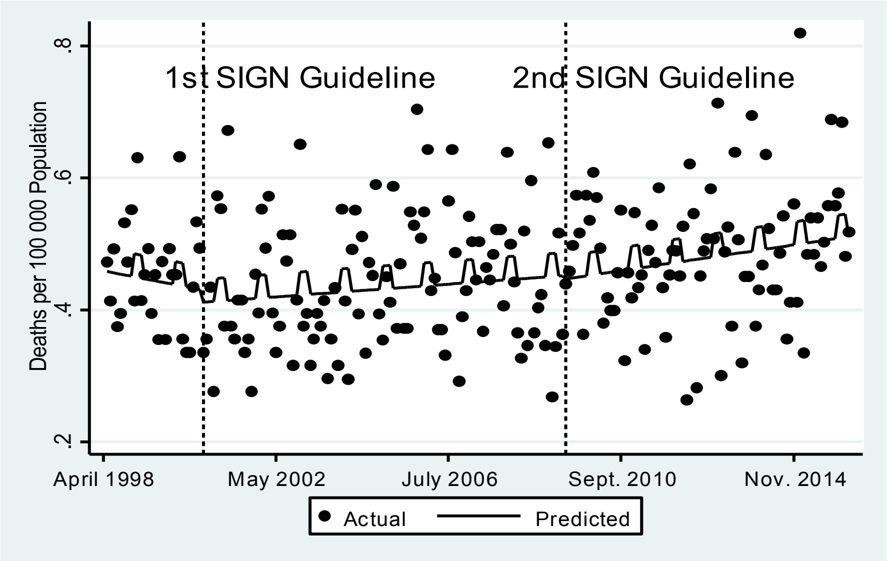

Supplementary Material 9: The impact of the SIGN guidelines on the percentage of patients admitted with TBI who died within 30 days of admission

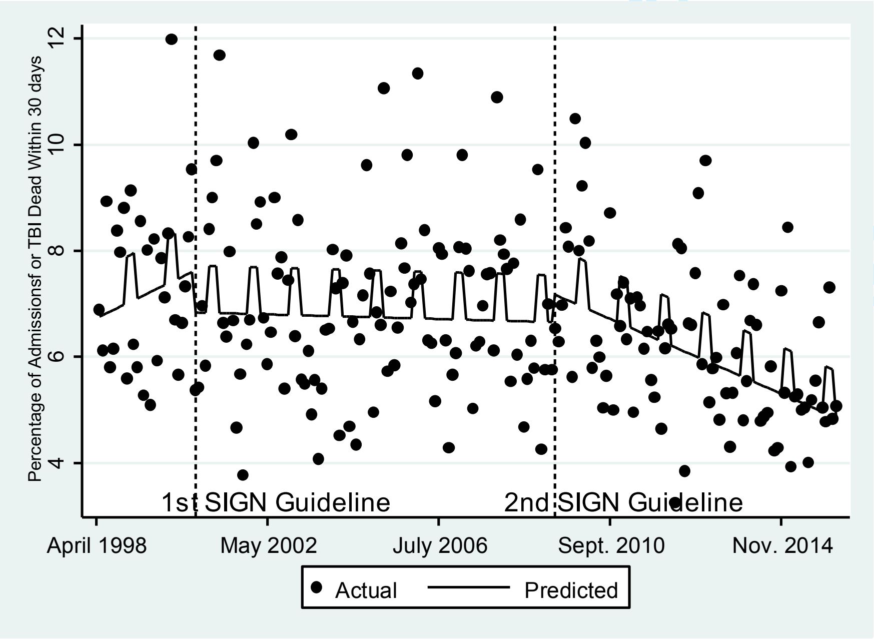

Supplementary Material 10: Estimate of impact of the SIGN guidelines on deaths following admission for Traumatic Brain Injury with 12 month time lag

Outcome	Winter	Initial Trend	1st SIGN Guideline	2nd SIGN Guideline	Durbin-Watson Statistic
Deaths/100 000	0.03 (95% CI:-0.01 to 0.06) P=0.15	-0.001 (95% CI:-0.004 to 0.003) P=0.59	Change level: -0.02 (95% CI:- 0.09 to 0.06) P=0.66	Change level: -0.03 (95% CI:-0.08 to 0.03) P=0.32	Untransformed 2.29
			Change trend: 0.001 (95% CI:-0.002 to 0.005) P=0.44	Change trend: 0.001 (95% CI:-0.0002 to 0.002) P=0.10	
Percentage TBI admissions death	0.74 (95% CI: 0.18 to 1.30) P=0.10	0.03 (95% CI:-0.03 to 0.10) P=0.34	Change level: -1.06 (95% CI: -2.40 to 0.29) P=0.12	Change level: -0.37 (95% CI:-1.20 to 0.47) P=0.39	Untransformed 2.17
			Change trend: -0.03 (95% CI:-0.10 to 0.04) P=0.38	Change trend: -0.02 (95% CI:-0.04 to -0.004) P=0.02	

For peer review only

STROBE Statement—checklist of items that should be included in reports of observational studies

	Item No	Recommendation
**Title and abstract**	1	(a) Indicate the study's design with a commonly used term in the title or the abstract **Page 1**
		(*b*) Provide in the abstract an informative and balanced summary of what was done and what was found **Page 2**
**Introduction**		
Background/rationale	2	Explain the scientific background and rationale for the investigation being reported **Page 4**
Objectives	3	State specific objectives, including any prespecified hypotheses **Page 4**
**Methods**		
Study design	4	Present key elements of study design early in the paper **Pages 5,6**
Setting	5	Describe the setting, locations, and relevant dates, including periods of recruitment, exposure, follow-up, and data collection **Pages 5,6**
Participants	6	(*a*) *Cohort study*—Give the eligibility criteria, and the sources and methods of selection of participants. Describe methods of follow-up **Pages 5,6**
		(*b*) *Cohort study*—For matched studies, give matching criteria and number of exposed and unexposed **Pages 5,6**
Variables	7	Clearly define all outcomes, exposures, predictors, potential confounders, and effect modifiers. Give diagnostic criteria, if applicable **Pages 6,9**
Data sources/ measurement	8*	For each variable of interest, give sources of data and details of methods of assessment (measurement). Describe comparability of assessment methods if there is more than one group **Pages 5,6,9**
Bias	9	Describe any efforts to address potential sources of bias **Pages 9,10**
Study size	10	Explain how the study size was arrived at **N/A**
Quantitative variables	11	Explain how quantitative variables were handled in the analyses. If applicable, describe which groupings were chosen and why **Pages 6,9**
Statistical methods	12	(*a*) Describe all statistical methods, including those used to control for confounding **Pages 9,10**
		(*b*) Describe any methods used to examine subgroups and interactions **Pages 9,10**
		(*c*) Explain how missing data were addressed
		(*d*) *Cohort study*—If applicable, explain how loss to follow-up was addressed *Case-control study*—If applicable, explain how matching of cases and controls was

For peer review only

		addressed<br>*Cross-sectional study*—If applicable, describe analytical methods taking account of sampling strategy
		(*e*) Describe any sensitivity analyses<br>**Page 10**
**Results**		
Participants	13*	(a) Report numbers of individuals at each stage of study—eg numbers potentially eligible, examined for eligibility, confirmed eligible, included in the study, completing follow-up, and analysed<br>**Page 7**
		(b) Give reasons for non-participation at each stage
		(c) Consider use of a flow diagram
Descriptive data	14*	(a) Give characteristics of study participants (eg demographic, clinical, social) and information on exposures and potential confounders<br>**Page 7**
		(b) Indicate number of participants with missing data for each variable of interest
		(c) *Cohort study*—Summarise follow-up time (eg, average and total amount)
Outcome data	15*	*Cohort study*—Report numbers of outcome events or summary measures over time
		Case-control study—Report numbers in each exposure category, or summary measures of exposure
		Cross-sectional study—Report numbers of outcome events or summary measures
Main results	16	(*a*) Give unadjusted estimates and, if applicable, confounder-adjusted estimates and their precision (eg, 95% confidence interval). Make clear which confounders were adjusted for and why they were included<br>**Pages 7, 10, 11, 12, 13**
		(*b*) Report category boundaries when continuous variables were categorized
		(*c*) If relevant, consider translating estimates of relative risk into absolute risk for a meaningful time period
Other analyses	17	Report other analyses done—eg analyses of subgroups and interactions, and sensitivity analyses
**Discussion**		
Key results	18	Summarise key results with reference to study objectives<br>**Pages 13, 14**
Limitations	19	Discuss limitations of the study, taking into account sources of potential bias or imprecision. Discuss both direction and magnitude of any potential bias<br>**Pages 14, 15, 16**
Interpretation	20	Give a cautious overall interpretation of results considering objectives, limitations, multiplicity of analyses, results from similar studies, and other relevant evidence<br>**Pages 16, 17**
Generalisability	21	Discuss the generalisability (external validity) of the study results<br>**Pages 16, 17**
**Other information**		
Funding	22	Give the source of funding and the role of the funders for the present study and, if applicable, for the original study on which the present article is based<br><br>**Page 18**

For peer review only - http://bmjopen.bmj.com/site/about/guidelines.xhtml

*Give information separately for cases and controls in case-control studies and, if applicable, for exposed and unexposed groups in cohort and cross-sectional studies.

Note: An Explanation and Elaboration article discusses each checklist item and gives methodological background and published examples of transparent reporting. The STROBE checklist is best used in conjunction with this article (freely available on the Web sites of PLoS Medicine at http://www.plosmedicine.org/, Annals of Internal Medicine at http://www.annals.org/, and Epidemiology at http://www.epidem.com/). Information on the STROBE Initiative is available at www.strobe-statement.org.

For peer review only
